# Integration of the D‐Genome Reshapes Gene Transcription, Chromatin Architecture, and Metabolome of Allohexaploid Wheat, Leading to Enhanced Environmental Adaptability

**DOI:** 10.1002/advs.77894

**Published:** 2026-09-27

**Authors:** Yanyan Liu, Tao Zhu, Ziru Zhou, Wei Chen, Chao He, Xinkai Zhou, Xin Wang, Chuanye Chen, Bo Yang, Jiaqi Wei, Caixia Lan, Mengmeng Liu, Handong Su, Qiang Li, Xin Hu, Siteng Bi, Weizhi Ouyang, Xingwang Li, Hailiang Mao, Masahiro Kishi, Kerstin Kaufmann, Alisdair R. Fernie, Dijun Chen, Wenhao Yan

**Affiliations:** ^1^ National Key Laboratory of Crop Genetic Improvement, Hubei Hongshan Laboratory Huazhong Agricultural University Wuhan China; ^2^ State Key Laboratory of Pharmaceutical Biotechnology, School of Life Sciences Nanjing University Nanjing China; ^3^ International Maize and Wheat Improvement Center (CIMMYT) Mexico City Mexico; ^4^ Department For Plant Cell and Molecular Biology, Institute For Biology Humboldt‐Universität Zu Berlin Berlin Germany; ^5^ Max Planck Institute of Molecular Plant Physiology Potsdam Germany; ^6^ Chemistry and Biomedicine Innovation Center Nanjing University Nanjing China

**Keywords:** 3D interactome, adaptability, epigenome, metabolome, polyploidizaiton, transcriptional regulation, wheat

## Abstract

The hybridization‐led integration of the D‐genome into domesticated tetraploid wheat gave rise to allohexaploid wheat, the most cultivated wheat globally grown across diverse environmental conditions. However, the regulatory mechanisms by which this genomic integration confers increased environmental adaptability in allohexaploid remains largely unexplored. Here, we investigated the change of transcriptome, chromatin interactome and metabolome in three independent polyploidization events. Our findings reveal that polyploidization triggers the activation of defense‐related genes through a comprehensive reorganization of the epigenome and 3D chromatin architecture. Furthermore, polyploidization significantly enriches secondary metabolites—specifically alkaloids and flavonoids—which are pivotal for environmental adaptation. We further found that B‐subgenome genes *TaRLK‐1B* and *TaRLK‐3B* are activated via *de novo* chromatin interactions to enhance alkali stress tolerance, whereas *TaGT‐2D* boosts UV‐B resistance through the accumulation of glycosylated flavonoids. These results shed light on the mechanistic basis of enhanced adaptability in hexaploid wheat, especially to salt, alkali, and UV‐B stress, and highlight the indispensable contribution of the D‐genome to the evolutionary success of the allohexaploid lineage.

## Graphical abstract

Integration of the D‐genome reshapes gene transcription, chromatin architecture, and the metabolome of hexaploid wheat, leading to enhanced adaptability to salt, UV‐B, and alkali stress.

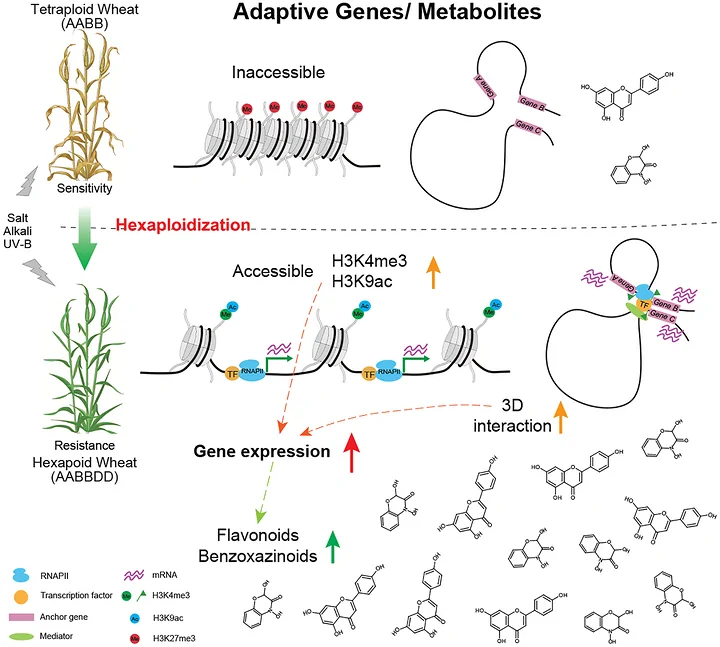

## 1 Introduction

Polyploidy, resulting from whole‐genome duplication (WGD) or interspecific hybridization, is a ubiquitous evolutionary phenomenon in eukaryotes [1], particularly among flowering plants [2, 3]. This process leads to an increased genome size and amplified genetic diversity, endowing polyploids with enhanced adaptive plasticity to fluctuating or extreme environments compared with their diploid counterparts. Consequently, polyploidization provided the genomic foundation for the successful domestication of numerous global crops [2, 4, 5]. Polyploidization remains, moreover, a driving force for plant evolution, speciation and biodiversity [3, 6, 7]. It is widely posited that the response to environmental stress plays a pivotal, if not determinative, role in the prosperity of polyploids [8].

Bread wheat (*Triticum aestivum* ssp. *aestivum*, AABBDD) is an allohexaploid with the largest global cultivation area among all crops. Its cultivation area expanded from the Fertile Crescent to almost all terrestrial areas of the globe, encompassing diverse climatic and environmental conditions within just a few millennia following polyploidization [9, 10, 11]. The evolutionary journey of bread wheat involved two successive natural hybridization events [12]. Initially, *T. urartu* (AA genome) intercrossed with a grass believed to be related to *Aegilops speltoides* (SS genome), resulting in the allotetraploid *T. turgidum* (AABB) following genome doubling [13, 14, 15]. Subsequently, another occasional interspecific hybridization amalgamated the D‐genome from diploid *Ae. tauschii* with the genome of *T. turgidum*, giving rise to the ancestral allohexaploid wheat, *T. aestivum* (AABBDD) [12, 16, 17], which was ultimately domesticated to form bread wheat. *Ae. tauschii* comprises three distinct lineages: lineage 1 (L1), lineage 2 (L2), and lineage 3 (L3). L2 is recognized as the main origin of the wheat D subgenome, whereas L3 is considered to be the other predominant donor of D subgenome in addition to L2. By contrast, L1 has negligible impact on bread wheat evolution [16, 17, 18].

Allohexaploid wheat shows enhanced tolerance to salt stress, zinc or nitrogen deficiency compared to tetraploid or diploid wheat [19, 20, 21]. Recent analyses have revealed that the introgression of genes from wild relatives played a pivotal role in improved environmental adaptation and agronomic traits during domestication [10, 17, 22, 23, 24, 25]. Studies using extracted or newly synthesized wheats have identified rapid genomic alterations, including sequence elimination, expression shifts, and dynamic DNA methylation changes, as key factors in wheat evolution [26, 27, 28, 29, 30, 31, 32]. Despite these insights, how the restructured genome and metabolome collectively facilitate broad adaptability remains poorly understood. Specifically, the mechanisms governing the response of hexaploid wheat to abiotic stresses, such as alkalinity and excessive UV‐B radiation, remain largely unexplored. Metabolites are not only pivotal determinants of crop quality but also active mediators of stress responses. For instance, flavonoids are known to play a critical role in abiotic stress responses [33, 34, 35, 36, 37], while terpenoids contribute to environmental adaptability and fruit quality [38, 39]. To date, however, a direct mechanistic link between chromatin‐mediated gene regulatory networks, metabolic outputs, and enhanced adaptability has not been established.

Epigenetic remodeling is significantly correlated with the homoeologous gene expression patterns in polyploids [40]. Recent advances in 3D genomics—utilizing Hi‐C, Hi‐ChIP, and OCEAN‐C to profile histone modifications and chromatin architecture — have underscored the role of regulatory elements and chromatin signatures in governing asymmetric gene transcription in wheat [41, 42, 43, 44, 45, 46, 47]. Nevertheless, the degree to which the epigenetic landscape and 3D chromatin interactome were reorganized upon the integration of the D‐genome remains unclear. Furthermore, the downstream impact of these changes on metabolic flux and environmental resilience is unknown.

To simulate the hexaploidization process, synthetic hexaploid wheat (SHW) can be generated through the crossing of tetraploid wheat *T. turgidum* with its diploid relative *Ae. tauschii*, followed by artificial chromosome doubling of the triploid F_1_ plants [48, 49, 50]. In this study, we utilized a cohort of SHWs and their parental tetraploid wheat and diploid *Ae. tauschii* to systematically decode how the D‐genome integration enhanced wheat adaptability by reshaping the transcriptome, epigenome, 3D chromatin interactome, and metabolome. We focused on the activation of genes and metabolic pathways linked to environmental fitness, with a specific emphasis on the mechanisms underlying alkaline, salt, and UV‐B resistance during polyploidization.

## 2 Results

### 2.1 Validation of SHW Materials and Phenotypic Evaluation

Hexaploid wheat is extensively cultivated across various global regions characterized by diverse environmental conditions (Figure S1), which underscores its remarkable adaptation and versatility. To elucidate the regulatory impact of D‐genome integration on environmental adaptability, we conducted stress (salt, alkaline, and UV‐B light) tolerance analysis using a cohort of genotypes, which included three synthetic hexaploid wheats (SHW3, SHW4 and SHW5), their common tetraploid parent (*T. turgidum* ssp. *durum*, AABB#7) and their independent diploid parents (*Ae. tauschii*, DD#10, DD#11, and DD#12) (Table S1). Notably, these diploid parents represent two distinct evolutionary lineages: DD#10 and DD#11 come from L2 (the primary D‐genome donor for modern wheat), while DD#12 belongs to L1 (a lineage with minimal evolutionary contribution to modern bread wheat) [16, 17] (Figure S2a). Fluorescent in situ hybridization (FISH) and genomic in situ hybridization (GISH) analyses unequivocally confirmed that all SHWs used in our study possessed the expected 42 chromosomes (2n = 6× = 42), while the tetraploid and diploid ancestors retained 28 (2n = 4× = 28) and 14 (2n = 14) chromosomes, respectively (Figure S2b–h).

**FIGURE 1:**
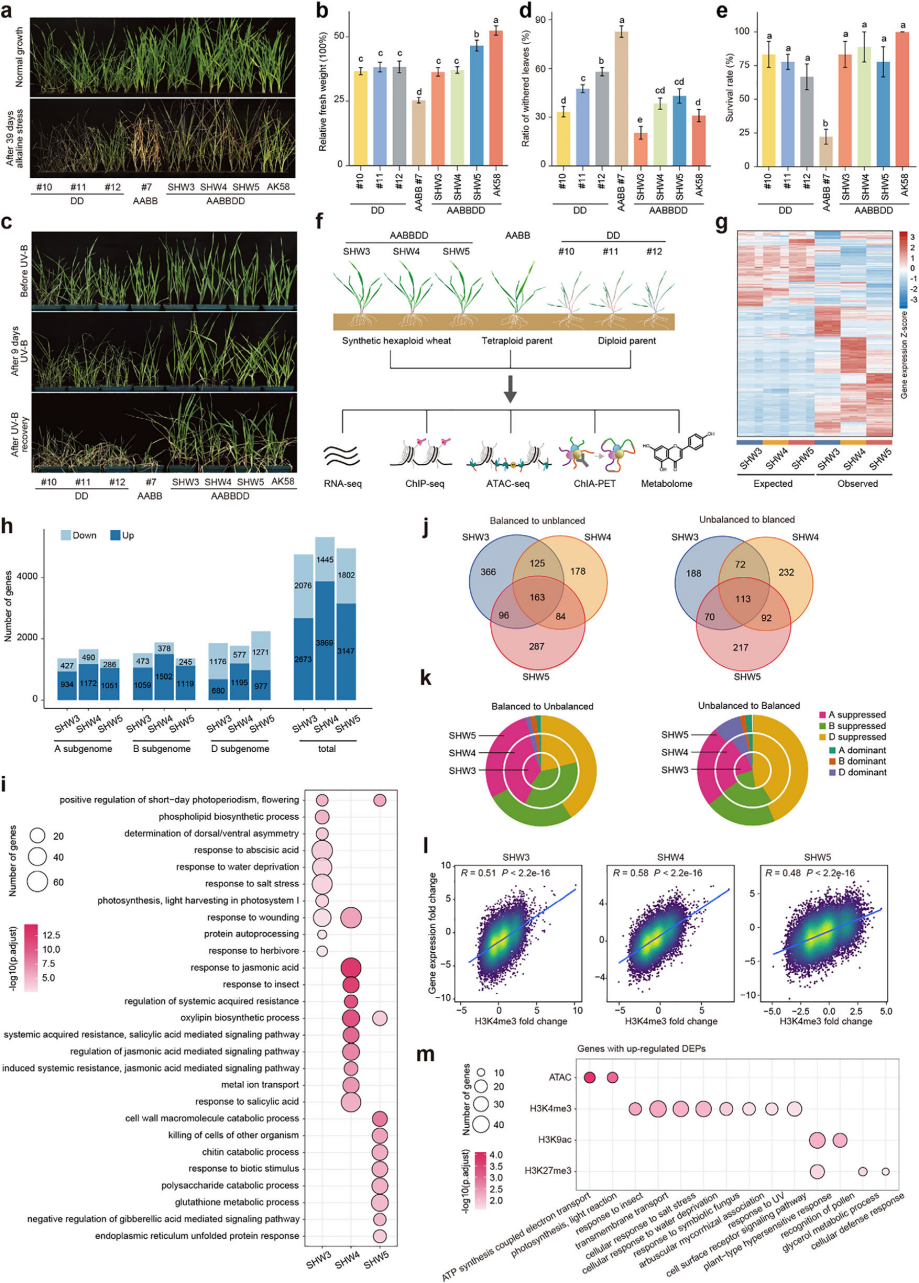
Reshaping of transcriptome and epigenome enhanced the abiotic stress tolerance of hexaploid wheat. (a) Phenotypic analysis of hexaploid wheat under alkaline stress. 18‐days seedlings were grown in soil without (normal condition) or with 100 mM mixed alkaline. Photographs were taken after 39 days of alkaline stress. (b) Statistical analysis of the relative fresh weight of seedlings in (a). Different lowercase letters above bars indicate significant differences among materials at *p* < 0.05 according to Student‐Newman‐Keuls test for multiple comparisons. (c) Phenotypic analysis of hexaploid wheat under UV‐B light stress. 18‐days seedlings were transferred to UV‐B chamber for 9 days treatment, then transferred back to normal light for 14 days recovery. (d,e) Statistical analysis of the relative withered leaves after 9‐days UV‐B radiation (d) and the survival rates after 14‐days recovery (e) in (c). Different lowercase letters above bars indicate significant differences among materials at *p* < 0.05 according to Student‐Newman‐Keuls test for multiple comparisons. (f) Schematic outlining the experimental design of multi‐omics analysis for D‐genome integration mediated hexaploidization events. The fully expanded leaves from 21‐days wheat seedlings were collected for RNA‐seq, ChIP‐seq, ATAC‐seq, ChIA‐PET and metabolome analysis. (g) Heatmap showing identified differentially expressed genes (DEGs) during wheat hexaploidization events. (h) Number of DEGs affected by the hexaploidization in different subgenomes of SHWs. Activated genes and suppressed genes were indicated respectively by dark blue and light blue bars. (i) Top ten GO terms enriched in up‐regulated DEGs. Dot size represents the gene number enriched in GO terms and color gradation represents significance of enrichment calculated as –log10 (FDR). (j) Numbers and overlapping of triads that changed asymmetric expression patterns (left, balanced to unbalanced; right, unbalanced to balanced) during the hexaploidization events. (k) Proportion of categories that altered their balance status during hexaploidization. (l) The relationship between gene expression fold change and peak fold change of H3K4me3 modifications in its promoter and intragenic regions. (m) Bubble plot showing the top enriched GO terms of genes with up‐regulated chromatin accessibility and histone modifications of H3K4me3 or H3K9ac during wheat hexaploidizations. The size of dots indicates number of genes in the terms, and the gradation of colors represents the significance as –log10 (FDR).

We took the widely grown hexaploid wheat variety Aikang 58 (AK58) [51] to serve as control for the abiotic tolerance analysis. When irrigated with 250 mM NaCl solution, hexaploids consistently outperformed their tetraploid and diploid progenitors (Figure S3a), maintaining substantially higher biomass (11.7% to 15.5% relative fresh weight in hexaploids versus 9.5% in tetraploids and 1.5% to 5.3% in diploids) after 43 days of treatment (Figure S3b), consistent with previous reports [20]. Under 100 mM alkaline stress, tetraploid wheat showed obvious growth inhibition with more withered leaves than observed in the hexaploidy (including the three SHWs and AK58) and diploidy after 39 days of alkaline irrigation (Figure 1a). The relative fresh weight of alkaline‐treated tetraploid plants is only 25.4% of the corresponding plants grown under normal conditions, significantly lower than alkaline‐treated hexaploid and diploid wheat (36.4% to 52.4% and 36.6% to 38.3%, respectively) (Figure 1b). When subjected to UV‐B light, 82.7% of the leaves of tetraploid wheat withered after 9 days, and only 22.2% of the plants survived after returning to normal conditions for 14 days. By contrast, hexaploid and diploid plants showed a lower proportion of withered leaves (20.4% to 43.1% in hexaploidy and 33.4% to 58.1% in diploidy) after UV‐B exposure, and 77.8% to 100% and 66.7% to 83.3% of the hexaploid and diploid plants survived, respectively (Figure 1c–e). These results confirmed the enhanced adaptability of hexaploid wheat to salt, alkali, and UV‐B stress after hexaploidization compared to that of the tetraploid parents.

### 2.2 Massive Activation of Adaptability Genes During D‐Genome Integration

To elucidate the regulatory mechanisms underlying the enhanced adaptability of hexaploids, we conducted a comprehensive multi‐omics analysis using the three SHWs (SHW3, SHW4, and SHW5), their shared tetraploid parents, and independent diploid parents (Figure 1f and Table S1). Transcriptome profiling (Figure S4 and Table S2) revealed that the total number of expressed genes (TPM > 0.5) was comparable across subgenomes (Figure S5a). To delineate specific transcriptomic shifts following polyploidization, we constructed a theoretical “expected” profile by aggregating parental expression data to simulate an in silico hexaploid [52]. This approach assumes an absence of inter‐subgenomic interaction, providing a baseline to evaluate the actual “observed” gene expression in real SHWs.

A comparative analysis between the “observed” and “expected” datasets identified 9830 polyploidization‐responsive differentially expressed genes (DEGs) in at least one of the SHWs (Figure 1g). Among these, up‐regulated DEGs (2673–3869) significantly outnumbered down‐regulated genes (1445–2076) across the three SHWs (Figure 1h and Tables S3–S5). Notably, while down‐regulation was more pronounced in the D subgenome (577 to 1271 genes), up‐regulation occurred at a similar magnitude across the A, B, and D subgenomes (Figure 1h). A significant overlap of DEGs was observed across different SHWs for both up‐regulated and down‐regulated genes (Figure S5b,c). Functional enrichment analysis revealed that these up‐regulated DEGs were predominantly associated with environmental adaptation, specifically terms related to “response to multiple stimuli” (Figure 1i and Figure S6a). In contrast, down‐regulated genes were primarily linked to basal “translation” processes, particularly within the A and B subgenomes (Figures S5d and S6b). To validate the evolutionary persistence of these polyploidization‐responsive gene expression patterns, we compared the identified DEGs with published transcription data from the elite variety AK58 and other globally cultivated hexaploids (e.g., TAM107, Manitou, Chinese Spring) [53, 54]. Remarkably, the majority of up‐regulated adaptation genes identified in SHWs maintained high expression levels in cultivated varieties and were enriched for adaptation‐related biological functions (Figure S7a–d), suggesting that these activation events were fixed during domestication to bolster broad environmental fitness.

We next looked into the asymmetric expression patterns after D‐genome integration. The homoeolog triad were categorized into seven expression patterns, ranging from “balanced” to “A‐ /B‐ /D‐ suppressed” (lower abundance of transcripts from one homoeolog than those from the other two homoeologs) or “A‐ /B‐ /D‐ dominant” (higher expression level of one homoeolog with respect to the other two homoeologs) [52]. The expressed triads (the sum TPM of A, B, D subgenome homoeologs > 0.5) were analyzed and we observed high correlation among genes within the same category across the three SHWs (Figure S8a). Homoeologous genes with changed triads expression pattern between “expected” and “observed” datasets were classified into two types, “balanced‐to‐unbalanced” and “unbalanced‐to‐balanced”. The former represents triads that changed from symmetrical (balanced) to asymmetrical (unbalanced, homoeolog‐suppressed or homoeolog‐dominant) expression patterns after hexaploidization (Tables S6–S8), while the latter indicates triads that shifted from unbalanced to balanced expression in SHWs (Tables S9–S11). Among the expressed triads in SHWs, 550–750 and 443–509 triads showed altered patterns from balanced to unbalanced and from unbalanced to balanced types, respectively (Figure 1j). Interestingly, most of the balanced‐to‐unbalanced triads tended to be constitutively expressed across various tissue types (Table S12), whereas the unbalanced‐to‐balanced triads exhibited both tissue‐specific and constitutive expression patterns (Figure S8b,c). Furthermore, almost all balanced‐to‐unbalanced triads were homoeolog‐suppressed, and only 0.9%–2.9% of triads became homoeolog‐dominant in SHWs (Figure 1k, Figure S9a–c and Tables S6–S8). Conversely, for the unbalanced‐to‐balanced type, a significant proportion of unbalanced categories that became balanced after D‐genome integration were homoeolog‐suppressed, while only 0.9% to 2.0% of triads for A‐ or B‐ dominant and 4.4% to 8.1% for D‐dominant patterns changed to balanced state following hexaploidization (Figure 1k; Figure S9d‐f and Tables S9‐S11). Collectively, these results demonstrate that hexaploidization orchestrates a genome‐wide transcriptomic reprogramming that activates critical adaptation pathways across all three subgenomes.

### 2.3 Altered Histone Modifications and Chromatin Accessibility During Wheat Hexaploidization Correlate with Transcriptional Changes of Genes Linked to Environmental Adaptability

Gene transcription is highly related to chromatin status, including histone modifications and chromatin accessibility. To study how hexaploidization reshapes the epigenome landscape, we mapped the genome‐wide distribution of H3K4me3, H3K9ac, and H3K27me3 modifications, alongside chromatin accessibility (by ATAC‐seq) in the SHWs and their tetraploid and diploid parents (Table S13). Correlation analysis revealed a significant positive relationship between changes in gene expression and chromatin accessibility and H3K4me3 or H3K9ac enrichment, while a consistently negative correlation was observed between H3K27me3 and gene transcription (Figure 1l and Figure S10). Recapitulating these trends, an integrated analysis of the previously identified up‐regulated and down‐regulated DEGs further substantiated the positive regulatory roles of H3K4me3, H3K9ac, and open chromatin states, while confirming the repressive impact of H3K27me3 on transcriptional output (Figure S11).

Following the same “expected” versus “observed” strategy applied in transcriptome analysis, we compared the “expected” and “observed” histone modification and chromatin accessibility status and observed a significant decrease in chromatin accessibility in the D subgenome after hexaploidization in all three SHWs (Figure S12). Additionally, we identified differentially enriched peaks (DEPs) derived from ATAC‐seq (corresponding to accessible genomic regions) and ChIP‐seq following polyploidization (Tables S14–S17). Genes with up‐regulated DEPs of ATAC‐seq and H3K4me3, H3K9ac modifications in their promoters or intragenic regions exhibited higher expression level after hexaploidization, and genes with down‐regulated DEPs were less expressed in SHWs. By contrast, genes with H3K27me3 DEPs exhibited the opposite trends (Figure S13). GO analysis revealed that genes with up‐regulated DEPs were enriched with terms related to environmental/external stimulus response, such as “cellular response to salt stress”, “response to UV” and “plant‐type hypersensitive response” (Figure 1m). These environmental response‐related genes showed higher expression level in SHWs (Figure S14a). While genes with down‐regulated DEPs were predominantly associated with “photosynthesis” or “translation” (Figure S14b). These results underscore the pivotal role of epigenetic reprogramming in coordinating the transcriptional activation of the wheat “adaptability toolkit” upon D‐genome integration.

### 2.4 Reorganization of 3D Chromatin Architecture May Serve as the Underlying Driving Force to Reshape Gene Transcription During Hexaploidization

**FIGURE 2:**
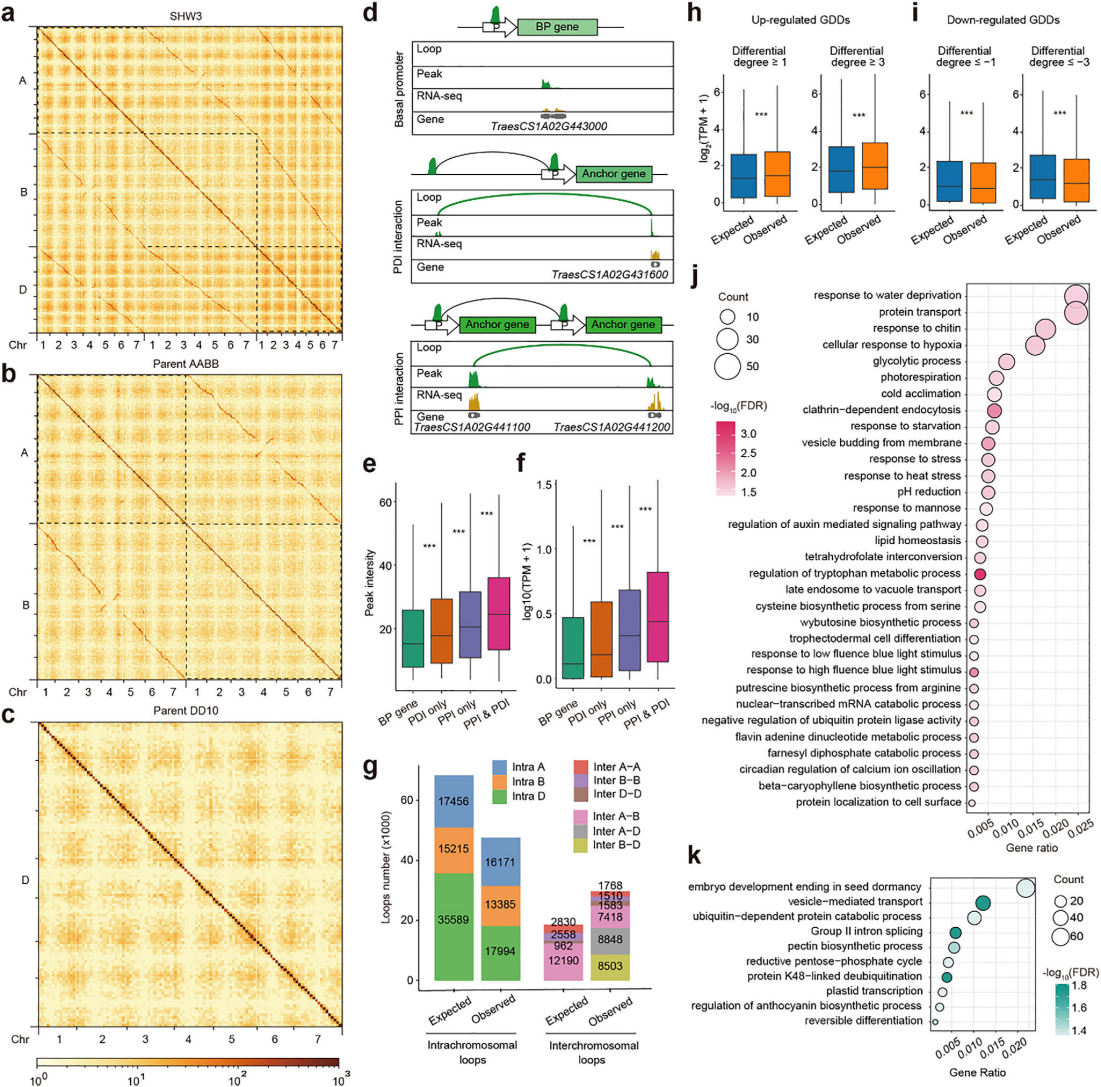
Dynamics of H3K4me3‐associated chromatin architecture following hexaploidization event. (a–c) Heatmaps showing H3K4me3‐associated chromatin interactions in SHW3 (a) and its tetraploid (b), diploid (c) parents. (d) Schematic models of H3K4me3‐associated chromatin interactions. BP model, genes with H3K4me3 modification that are not involved in chromatin interactions. PPI model, anchor genes involved in H3K4me3‐associated proximal interactions. PDI model, anchor genes involved in interactions between H3K4me3‐associated proximal and distal modification region. (e,f) Boxplot showing the intensity of H3K4me3 modification in different chromatin models (e) and the expression level of BP genes and genes involved in PDI, PPI interactions (f) in SHW3. The gene expression levels are shown as log10(TPM + 1). ***p < 0.001, two‐sided *t*‐test. (g) Comparison of the intrachromosomal and interchromosomal loops in SHW3 before and after hexaploidization. (h,i) Boxplots showing the expression level of up‐regulated GDDs (h) and down‐regulated GDDs (i) in expected and observed dataset of SHW3 when |differential interaction degree| ≥1 or ≥3. ***p < 0.001, two‐sided t‐test. (j,k) Bubble diagram showing enriched GO terms of up‐regulated GDDs (j) and down‐regulated GDDs (k) in SHW3. The dot size represents the number of genes in the corresponding GO term, and the gradation of color indicates the significance of terms as –log10 (FDR).

Collective evidence suggests that long‐range chromatin interactions have a significant impact on gene transcription [55, 56, 57, 58, 59]. Given the close association of H3K4me3 with gene transcription and the enrichment of H3K4me3‐modified genes with various GO terms (Figure 1l,m and Figure S14), H3K4me3 modification can be used as an indicative mark of gene transcription to explore the impact of D‐genome integration‐mediated hexaploidization. Most of these H3K4me3 DEPs showed similar alteration trends during the different hexaploidization processes of SHW3, SHW4, and SHW5, including different adaptive genes (Figure S15). To examine the changes of H3K4me3‐associated chromatin interactions during wheat hexaploidization, we performed long‐read ChIA‐PET associated with H3K4me3 in SHW3 and its tetraploid and diploid parents (Figure 2a–c and Table S18), considering that its D‐genome donor DD#10 belongs to the L2 lineage, the primary D‐genome source for modern wheat. The highest number of H3K4me3‐associated chromatin interactions was detected within the D subgenome, followed by the A subgenome, with the fewest interactions occurring within the B subgenome (Figure 2a and Figure S16a). This observation is consistent with published Hi‐C data [44, 45]. In the tetraploid progenitor, chromatin interactions within the A subgenome occur at higher frequency than within the B subgenome (Figure 2b and Figure S16b). Besides, we detected more loops among homeologs than non‐homeologs, with the highest interaction frequency observed in chromosome group 2 (2A, 2B, and 2D) and group 5, and the lowest interaction frequency in chromosome group 4 (Figure S16c,d).

Genes with chromatin loops and H3K4me3 enrichment in their promoters are H3K4me3‐associated anchor genes, while genes with H3K4me3 modification but without chromatin loops are defined as BP genes (genes with a basal promoter). Depending on whether the chromatin‐loop involved peaks were located in the proximal (3.5 kb upstream of transcription start site [TSS] to transcription end site [TES]) or distal genic region (genomic regions out of proximal region), chromatin interactions were divided into proximal‐proximal interactions (PPIs) and proximal‐distal interactions (PDIs) (Figure 2d). Compared with BP genes, anchor genes exhibited significantly higher H3K4me3 levels in their promoters and showed higher expression levels. Indeed, anchor genes with both PPI and PDI chromatin interactions displayed the highest H3K4me3 modification intensity and expression levels among all wheat genes (Figure 2e,f). Anchor genes with higher interaction degree possessed a higher median H3K4me3 peak intensity and displayed a higher expression level (Figure S17a,b).

Similar to gene expression analysis, we next generated a virtual “expected” chromatin interaction map based on the chromatin interactions in tetraploid and diploid parents and then compared it with the chromatin interactions in SHW3 (designated as the “observed” dataset) to investigate the impact of altered chromatin interactions on gene transcription during D‐genome integration. In total, we observed 77180 interaction loops connecting 46445 anchor genes in SHW3, compared with 86800 interactions and 50605 anchor genes in the virtual “expected” dataset (Figure S17c,d). The anchored up‐regulated DEGs showed a higher chromatin interaction degree in the observed data, whereas the anchored down‐regulated DEGs interacted with less chromatin regions after hexaploidization (Figure S17e,f). Notably, 47901 loops were exclusively found before hexaploidization, while 38281 loops were specifically detected following the D‐genome integration (Figure S17c; Tables S19 and S20). It is remarkable that more interchromosomal interactions were produced following the D‐genome integration. This is likely due to the combinatorial effects from the reduction of intrachromosomal interactions in the D subgenome and the interchromosomal interactions between A and B subgenomes and the increase of newly established interactions between A–D and B–D subgenomes (Figure 2g).

Next, we compared the “observed” interaction degrees of all anchor genes with “expected” dataset in order to identify genes with different degrees (GDDs). A total of 17356 genes exhibited increased degrees (up‐regulated GDDs), including 3892 genes that changed from BP genes to anchor genes. In contrast, 24902 genes displayed decreased degree of interactions (down‐regulated GDDs), including 8052 genes that shifted from anchor genes to BP genes following hexaploidization (Figure S17d and Table S21). While up‐regulated GDDs were distributed equally across the A and B subgenomes but were less abundant in the D subgenome, more down‐regulated GDDs were found in the D subgenome than in the A and B subgenomes (Figure S17g,h). These up‐regulated GDDs showed a significantly higher expression level, whereas down‐regulated GDDs showed a significantly lower expression level in the “observed” dataset (Figure 2h,i). Both of the up‐regulated GDDs and down‐regulated GDDs with higher differential degree also showed a higher interaction degree (Figure S17i,j). We next conducted GO analysis using GDDs with |differential interaction degree| ≥ 3, and a total of 3397 up‐regulated GDDs and 4971 down‐regulated GDDs were used (Table S21). In addition to the genes for basic physiological processes such as protein transport and glycolytic process, genes for response to environmental stimuli such as water deprivation, chitin, cold acclimation, pH reduction and heat stress were significantly enriched in up‐regulated GDDs but not in down‐regulated GDDs (Figure 2j,k and Table S22).

We also compared the chromatin interactions of the cultivated variety AK58 with expected data generated in this study and similar results were obtained. The reduced intrachromosomal interactions in D subgenome and interchromosomal interactions between A and B subgenomes, and the newly established interactions between A–D and B–D subgenomes were also detected (Figure S18a). We identified 49457 GDDs including 23675 up‐regulated GDDs and 25782 down‐regulated GDDs (Table S23), with less up‐regulated GDDs and more down‐regulated GDDs located in the D subgenome than that in the A or B subgenomes (Figure S18b,c). Up‐regulated GDDs were also enriched with a variety of environmental adaptability or signal transduction‐related GO terms, such as response to water deprivation, protein autophosphorylation, and response to chitin. By contrast, only two GO terms were enriched in these down‐regulated GDDs, namely “RNA modification” and “protein processing” (Figure S18d,e and Table S24).

### 2.5 Activation of Adaptive Genes Through Enhanced Chromatin Interaction Facilitates Environmental Adaptability Following Hexaploidization

**FIGURE 3:**
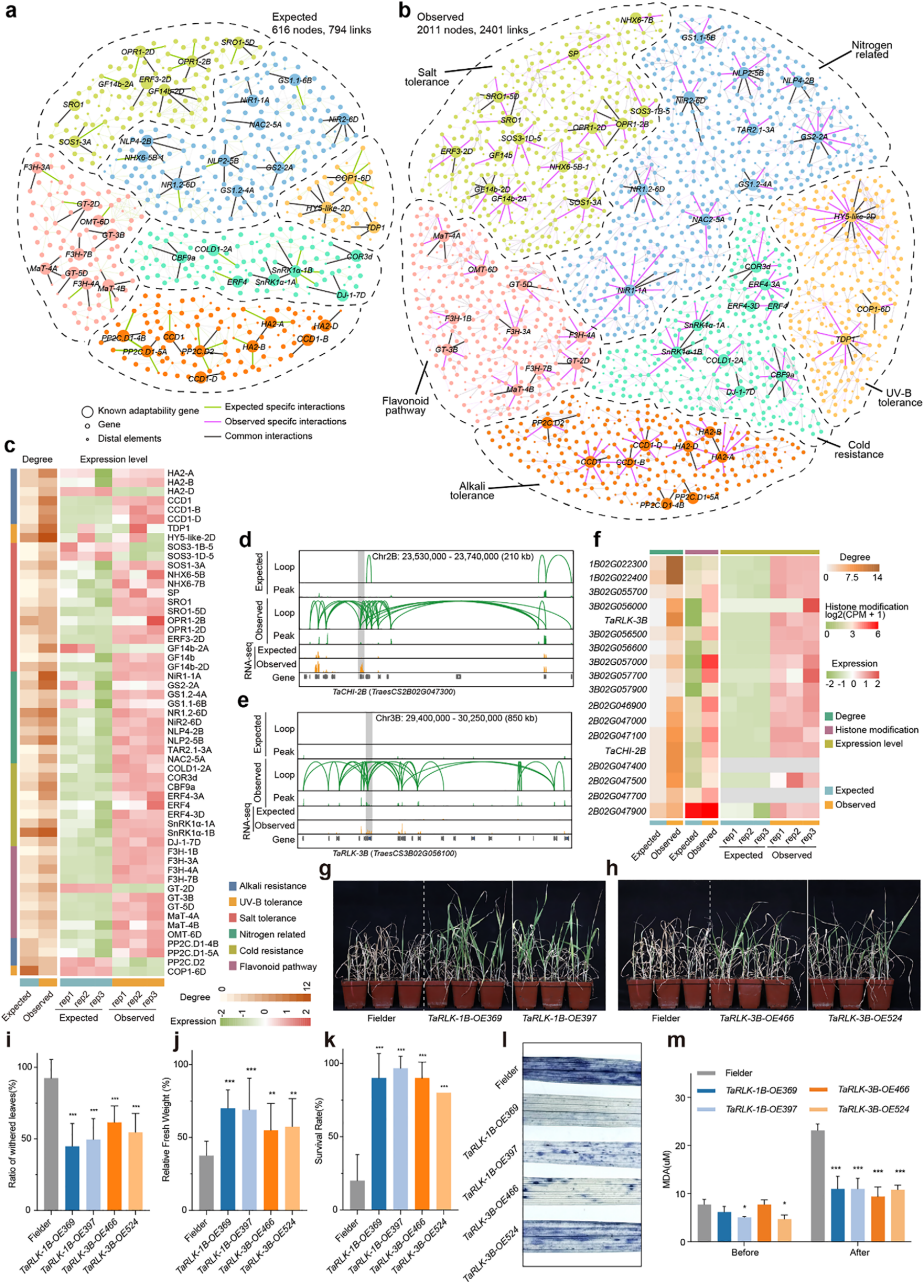
Activation of adaptive genes through enhanced H3K4me3‐associated chromatin interactions after hexaploidization. (a,b) Chromatin interaction networks built using adaptive genes before (a) and after (b) hexaploidization. Different dot colors represent nodes from different networks of UV‐B tolerance, alkali tolerance, salt tolerance, cold resistance, nitrogen‐related genes, and flavonoid pathway. The light green lines represent specific chromatin interactions before hexaploidization (expected dataset). The light purple lines indicate newly formed interactions after D‐genome integration (observed dataset). The black lines indicate common interactions both in expected and observed networks. (c) Heatmap showing increased interaction frequency (degree) and enhanced expression level of the adaptation related genes. (d,e) A browser screenshot showing the H3K4me3‐associated chromatin interactions, modification peaks, and RNA‐seq data in the actively transcribed region containing TaCHI‐2B (d) or TaRLK‐3B (e) in expected and observed datasets. (f) Heatmap showing increased chromatin interaction degree, enhanced histone modification, and activated transcription of the genes following hexaploidization. (g,h) The TaRLK‐1B‐OE (g) and TaRLK‐3B‐OE (h) transgenic plants showed enhanced alkaline stress tolerance. Fielder, wild‐type plants. (i–k) TaRLK‐1B‐OE and TaRLK‐3B‐OE transgenic plants showed a significantly decreased ratio of withered leaves (i), increased relative fresh weight (j), and enhanced survival rate (k) after alkaline stress compared with wild‐type plants (Fielder). Asterisks indicate significant differences between wild‐type and transgenic plants at *p < 0.05, **p < 0.01, ***p < 0.001 by two‐sided *t*‐test. (l) NBT staining analysis showing less H_2_O_2_ were accumulated in the leaves of TaRLK‐1B‐OE and TaRLK‐3B‐OE transgenic plants. (m) The MDA content significantly decreased in TaRLK‐1B‐OE and TaRLK‐3B‐OE transgenic plants after alkaline treatment. Asterisks indicate significant difference between wild‐type and transgenic plants at *p < 0.05, **p < 0.01, ***p < 0.001 by two‐sided *t*‐test.

A large number of DEGs undergo epigenetic remodeling or chromatin interaction changes during hexaploidization (Figure S19a,b), and these upregulated DEGs are significantly enriched in adaptive‐related GO terms (Figure S19c). To further reveal the impact of changes in chromatin modifications and interactions by D‐genome integration, gene networks were constructed using 1545 core DEGs that showed altered H3K4me3 modification or with |differential interaction degree| ≥ 3. Interestingly, most of these genes were not connected with each other in the parental AABB or DD genomes (Figure S20a, b), but after hexaploidization, they were linked by chromatin loops to form a large regulatory module following D‐genome integration (Figure S20c). To get a full picture of chromatin interaction in enhanced adaptability after polyploidization, we collected adaptability‐related genes from published studies and identified their wheat orthologues (Table S25). It revealed that numerous genes exhibited increased chromatin interaction frequency and elevated expression levels following hexaploidization, including genes related to UV‐B tolerance, alkaline tolerance, salt tolerance, cold resistance, nitrogen assimilation, as well as the flavonoid biosynthesis pathway (Figure 3a–c and Figure S20d,e). For example, *TaCCD1* known as positive regulators in wheat alkali stress tolerance [60], and *TaERF4* identified in Tibetan semi‐wild wheat for their adaptation to cold acclimation in high‐altitude environments [61], showed enhanced interaction degrees in “observed” networks accompanied by increased transcription (Figure 3a–c).

We noticed that a wheat chitinase encoding gene, *TaCHI‐2B* (*TraesCS2B02G047300*), which is involved in the plant defense response by hydrolyzing chitin [62, 63], formed chromatin loops with eight other genomic regions in the “observed” dataset compared with only one loop in the “expected” data and was accompanied by increased levels of histone modification and transcriptional activation following hexaploidization (Figure 3d,f). In addition, a group of receptor‐like kinases (RLKs) encoding genes also displayed increased interaction degrees, enhanced H3K4me3 modification, and elevated expression following D‐genome integration (Figure 3e,f). RLKs have been reported to play important roles in sensing diverse environmental changes and are involved in biological process related to biotic and abiotic stress [64, 65, 66]. We verified the enhanced enrichment of H3K4me3 modification in *TaCHI‐2B* and *TaRLK‐3B* genomic regions, and the newly built chromatin interactions of *TaRLK‐3B*, through ChIP‐qPCR and quantitative chromosome conformation capture (3C‐qPCR) strategies (Figure S21a,b). To determine the function of RLK genes that showed largely increased chromatin interaction frequency after hexaploidization, we constitutively expressed *TaRLK‐1B* (*TraesCS1B02G022400*) and *TaRLK‐3B* (*TraesCS3B02G056100*), respectively (Figure S21c). After alkaline stress, both the positive *TaRLK‐1B*‐OE and *TaRLK‐3B*‐OE plants showed increased alkaline tolerance than wild‐type plants (Figure 3g,h). The ratios of withered leaves were significantly lower in *TaRLK‐1B*‐OE (26.7%–30%) and *TaRLK‐3B*‐OE lines (10%‐13.3%) than those in wild‐type plants (78.6%) (Figure 3i). As a result, the relative fresh weight and survival rate of *TaRLK‐1B*‐OE and *TaRLK‐3B*‐OE lines were significantly higher than those of wild‐type plants (Figure 3j,k). The result of nitrotetrazolium blue chloride (NBT) staining to detect H_2_O_2_ content in the leaves showed less superoxide accumulation in these transgenic plants (Figure 3l), and the malondialdehyde (MDA) content was also reduced in *TaRLK‐1B*‐OE and *TaRLK‐3B*‐OE plants (Figure 3m), suggesting that *TaRLK‐1B* and *TaRLK‐3B* play a positive role in alkaline stress resistance. These results showed that the integration of D‐genome reshapes 3D chromatin interaction network of adaptive genes to regulate the gene expression atlas and enhance the alkaline adaptability of hexaploid wheat.

### 2.6 D‐Genome Integration Mediated Hexaploidization Led to Comprehensive Alterations in Metabolome

Domestication has been demonstrated to profoundly alter metabolite levels in various species, including tetraploid wheat, potato, and radish [67, 68, 69]. Our results revealed that one of the most significant features during D‐genome integration is the enhanced capacity and expression of genes related to environmental adaptability (Figures 1i,m, 2j, and 3). However, the link between adaptability and metabolites, especially secondary metabolites which are crucial in plant responses to environmental stimuli [39, 70], thereby influencing adaptability during hexaploidization, remains unclear. To explore metabolome changes following the D‐genome integration, we conducted liquid chromatography‐tandem mass spectrometry (LC‐MS/MS) analysis on the allohexaploid SHWs and their tetraploid and diploid parents [71]. A total of 839 metabolites were detected, and 477 metabolites were putatively annotated, including 84 alkaloids, 76 amino acids and peptides, 64 carbohydrates, 84 fatty acids, 151 phenylpropanoids and 18 terpenoids (Table S26).

**FIGURE 4:**
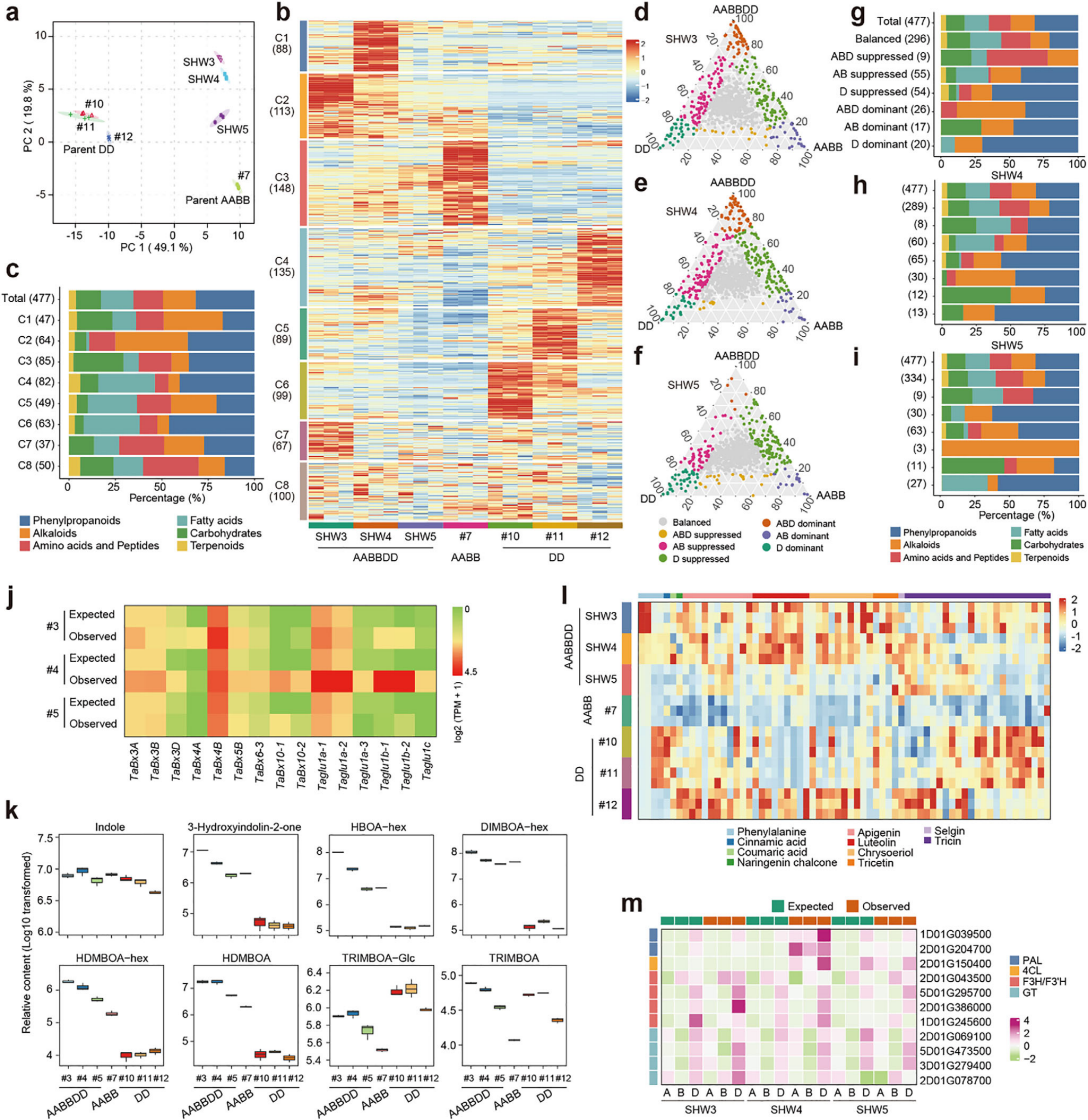
Metabolome analysis of hexaploid SHWs and the tetraploid, diploid parents. (a) Principal component analysis (PCA) of metabolome data generated from SHWs and its parents. Different colors and shapes represent different samples, n = 3. (b) Heatmap showing eight major clusters grouped based on the contents of a total of 839 metabolites in hexaploid SHWs, tetraploid and diploid parents. The number of metabolites in each cluster is given in parentheses. (c) Proportions of metabolite classes with annotated structures within the eight clusters. Different colors indicate the six different metabolite classes. The proportions of all the 472 metabolites in the six classes with known structures is shown. (d–f) Ternary plots showing diversity of metabolites between SHW (AABBDD) and its tetraploid (AABB), diploid (DD) parents of SHW3 (d), SHW4 (e), SHW5 (f). The balanced type of metabolites that showed similar content in the three materials are shown as grey dots, and the unbalanced type are shown as colored dots. (g–i) Proportions of metabolite classes with annotated structures within the seven categories shown in (d–f) of SHW3 (g), SHW4 (h), SHW5 (i). (j) Heatmap showing the activation of core BX genes in SHWs after D‐genome integration. Expected, expression level before hexaploidization; observed, expression level after hexaploidization. Expression levels are shown as log2 (TPM + 1)‐transformed values. (k) Relative contents of core metabolites in BX pathway of hexaploid SHWs and its hexaploidization parents. Metabolite contents are shown as log10‐transformed values. (l) Heatmap showing flavonoid pathway metabolites with high contents in hexaploid SHWs (AABBDD) compared with its tetraploid parent (AABB). Different colors on the top of the heatmap indicate core flavonoid metabolites. (m) Putative flavonoid pathway triad genes whose D homoeolog showed highest expression level compared with A and B homoeologs.

Principal component analysis (PCA) using these 839 detected metabolites clearly distinguished the allohexaploid SHWs from their tetraploid and diploid parents. Notable differences were observed in DD#12 from DD#10 and DD#11, and SHW5 from SHW3 and SHW4, respectively (Figure 4a), in line with the fact that DD#12 belongs to L1 (Figure S2a). Metabolites were further categorized into eight clusters based on their relative contents across different genotypes. Hexaploid SHWs exhibited the highest content of metabolites in clusters 1 and 2, whereas metabolites in cluster 3 showed the highest content in tetraploid wheat, and metabolites in clusters 4–6 were highly enriched in *Ae. tauschii* (genomes DD) than any polyploids (Figure 4b). Alkaloids were more prevalent in clusters 1 and 2, while carbohydrates were prominent in cluster 3, and fatty acids were enriched in clusters 4–6 (Figure 4c). All the above results indicate extensive changes in metabolite levels during hexaploidization. Metabolites were also classified into seven categories, analogous to the asymmetric gene expression analysis to study their dynamic changes during polyploidization (Figure 4d–f). Approximately 64.8% to 71.3% of metabolites exhibited a balanced pattern among SHWs and their parents. Notably, alkaloids and phenylpropanoids showed the most significant changes (Figure S22a–c). In addition, alkaloids are enriched in the ABD dominant categories in SHWs, and carbohydrates are prominent in AB dominant categories (Figure 4g–i). These results suggest that D‐genome integration mediated polyploidization led to reduced levels of carbohydrates and increased levels of secondary metabolites, particularly alkaloids, in allohexaploid SHWs.

Benzoxazinoids (BXs) are a class of indole‐derived plant metabolites that belong to alkaloids and most specially present in Poaceae plants and some dicots, such as wheat, maize and rye, and showed function in plant biotic, abiotic resistance and allelopathic phenomenon [72, 73, 74, 75]. The BX biosynthesis pathway starts from indole‐3‐glycerol phosphate, which is hydrolyzed to indole by an indole‐glycerol phosphate aldolase (Bx1). Indole was then further converted by four cytochrome P450 enzymes (Bx2‐Bx5) [76] and one UDP‐glucosyltransferase (UGT, Bx8, Bx9) to form HBOA (2‐hydroxy‐3,4‐dihydro‐2H‐1,4‐benzoxazin‐3‐one), DIBOA [2,4‐Dihydroxy‐2H‐1,4‐benzoxazin‐3(4H)‐one] and DIBOA‐Glc [76, 77], which were finally converted to other derivatives by Bx6, Bx7, Bx10 [73, 78, 79] and other unknown enzymes (Figure S23a). BXs are stored as inactive and stable glycosides in the vacuole where they wait their release by conversion to aglucones by β‐glucosidases [80, 81]. We have identified 18 metabolites belonging to the Bx pathway (Figure S23a). It is remarkable that many benzoxazinoids (BXs) belong to the ABD‐dominant category, in which metabolites showed higher content over tetraploid and diploid parents in ternary plot analysis (Figure 4d–f and Table S26). We noticed that most of the wheat BX biosynthesis genes showed increased expression levels following hexaploidization, especially *TaBx3*, *TaBx4*, *TaBx5*, and *Taglu* (Figure 4j), which resulted in obviously higher content of almost all metabolites in the BX pathway in SHWs compared with either their tetraploid or diploid parents (Figure 4k and Figure S23b). The higher expression levels of B homoeologs of *TaBx3*, *TaBx4*, *TaBx5* and *Taglu* (*Taglu1a*, *Taglu1b*) compared to A and D homoeologs were also demonstrated here (Figure 4j), which is in line with the observation that B subgenome homoeologs of wheat BX genes mainly contributed to BXs synthesis [76, 77]. In contrast, BX pathway genes from the D genome may contribute little to BX biosynthesis in hexaploid wheat, considering the low or even lack of expression of D homoeologs. There may be unknown activators that are introduced from the D‐genome integration event, which increase transcriptional efficiency and enhance their expression level, resulting in an overdominance phenomenon of BX biosynthesis in the hexaploid wheat.

Flavonoids are known for their roles in response to various stresses such as oxidative and drought stress, UV light and pathogen infections [33, 34, 35, 36, 37]. In plants, flavonoids are usually stored as glycosides in vacuoles, with the activities of glycosyl transferases being a key step in their accumulation [82, 83]. We noticed that several glycosyl derivatives of flavonoid synthesis were present at very low levels in AABB genomes but at high levels in both DD and AABBDD genomes (Figure 4l and Figure S24), suggesting that the increased content of flavonoids in SHWs results from the D‐genome integration. Strikingly, the expression levels of the D‐genome orthologs of rice flavonoid biosynthesis genes were significantly higher than those of A and B members both before and after the hexaploidization (Figure 4m). This higher expression level was attributed to the synergistic effect between active and repressive epigenetic markers (Figure S25a–c). Additionally, we observed the activation of several putative flavonoid biosynthesis pathway genes following D‐genome integration (Figure S25d). These findings demonstrate that the activation of the flavonoid pathway in allohexaploid wheat is attributed to combined effects of the introduction of novel functional genes via the D‐genome integration and the upregulation of genes in the A and B subgenomes.

Besides these rate‐limited enzyme encoding genes, we also conducted correlation analysis to identify genes that the expression level significantly (*p* value < 0.01) positive or negative correlated with BXs or flavonoids content in SHWs and its parents (Tables S27 and S28). The expression level of these positive‐related genes showed significant increase in SHWs than that in their parent plants (Figure S26a,b), corresponding to higher chromatin accessibility and chromatin interaction degree, also enriched H3K4me3 and H3K9ac modifications, but decreased H3K27me3 enrichment in their promoter or intragenic regions. By contrast, negative‐related genes showed lower expression level, lower chromatin accessibility, chromatin interaction, and H3K4me3, H3K9ac modification, and higher H3K27me3 enrichment (Figures S27 and S28). These results suggested that the activation of benzoxazinoid and flavonoid pathways is the result of coordinated regulation of epigenome, chromatin interactome, and chromatin accessibility.

### 2.7 *TaGT‐2D* Plays a Positive Role in UV‐B Tolerance of Hexaploid Wheat Through Glycosylated Flavonoids Accumulation

**FIGURE 5:**
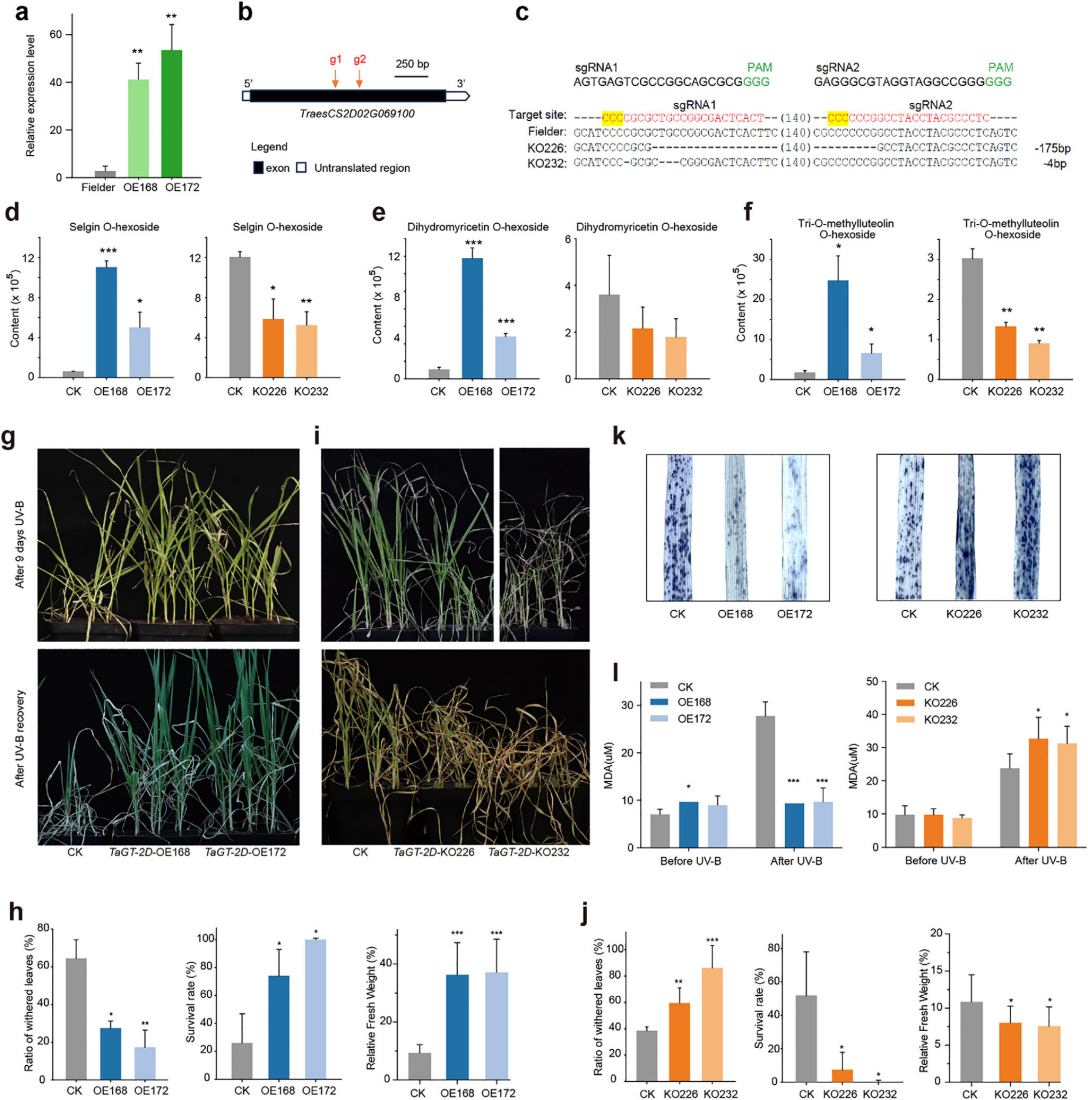
Functional identification of TaGT‐2D in flavonoids accumulation and UV‐B radiation tolerance. (a) The expression level of TaGT‐2D in the overexpression transgenic lines. Asterisks indicate a significant difference between wild‐type and transgenic plants at **p < 0.01 by two‐sided *t*‐test. (b) Scheme showing the structure of TaGT‐2D gene and the target sites of designed sgRNAs. Black rectangle represents the exon region of TaGT‐2D and white rectangle indicates the untranslated regions. g1, sgRNA1; g2, sgRNA2. (c) The genotype identification of different TaGT‐2D‐KO lines. The green or highlight letters represent the PAM site in sgRNA1 and sgRNA2. The number ‐175 and ‐4 indicate the deleted bases in TaGT‐2D‐KO226 and TaGT‐2D‐KO232 respectively. (d–f) Metabolome data showed that TaGT‐2D plays a positive role in the accumulation of glycosylated flavonoids Selgin O‐hexoside (d), Dihydromyricetin O‐hexoside (e), and Tri‐O‐methylluteolin O‐hexoside (f). Asterisks indicate significant differences between CK and transgenic plants at *p < 0.05, **p < 0.01, ***p < 0.001 by two‐sided *t*‐test. (g) The overexpression of TaGT‐2D enhanced the UV‐B tolerance of hexaploid wheat. (h) Statistical analysis of the relative withered leaves after 9 days of UV‐B, and the survival rates and relative fresh weights after 14 days of recovery of TaGT‐2D‐OE seedlings. (i) The loss‐of‐function mutation of TaGT‐2D enhanced the UV‐B sensitivity of hexaploid wheat. (j) Statistical analysis of the relative withered leaves after 9 days of UV‐B, and the survival rates and relative fresh weights after 14‐day recovery of TaGT‐2D‐KO seedlings. (k) NBT staining analyses showing the reduced H_2_O_2_ accumulation in leaves of TaGT‐2D‐OE lines (left), and increased H_2_O_2_ production in leaves of TaGT‐2D‐KO lines (right). (l) TaGT‐2D‐OE leaves demonstrated a significant decreased MDA content after UV‐B radiation (left), and TaGT‐2D‐KO plants accumulated more MDA in their leaves significantly. Asterisks indicate significant differences between CK and transgenic plants at *p < 0.05, **p < 0.01, ***p < 0.001 by two‐sided *t*‐test.

Considering the predominance of glycosylated flavonoids in SHWs, we generated overexpression transgenic lines and knockout mutant lines of D homoeolog gene *TraesCS2D02G069100* (namely *TaGT‐2D*), which encodes an *O*‐glucosyltransferase. The positive overexpression lines *TaGT‐2D*‐OE168 and *TaGT‐2D*‐OE172 showed 41‐ and 53‐fold (*p* < 0.01) higher expression levels of *TaGT‐2D* than wild‐type plants (Figure 5a). While the two independent knockout lines *TaGT‐2D*‐KO226 and *TaGT‐2D*‐KO232 carried 175‐bp and 4‐bp deletions in the coding region of *TaGT‐2D*, respectively (Figures 5b,c). The metabolome data of transgenic plants suggested that the contents of glycosylated flavonoids, Selgin *O‐*hexoside, Dihydromyricetin *O‐*hexoside, and Tri‐*O*‐methylluteolin *O‐*hexoside, significantly increased in *TaGT‐2D*‐OE lines and decreased in different *TaGT‐2D*‐KO lines (Figure 5d–f and Figure S29), demonstrating the *in vivo O*‐glucosyltransferase activity of TaGT‐2D in wheat plants.

To further verify the important role of flavonoids in plant UV‐B resistance, we assessed the responses of *TaGT‐2D*‐OE and *TaGT‐2D*‐KO plants to UV‐B radiation. After 9 days' treatment, the *TaGT‐2D*‐OE plants showed a better growth status than negative plants (Figure 5g), with 27.5% and 17.3% leaves being withered in *TaGT‐2D*‐OE plants, significantly less than 64.5% in negative plants. After recovery under normal light conditions, 74.1% and 100% *TaGT‐2D*‐OE plants survived, significantly higher than 25.9% that in negative plants. The relative fresh weight is 36.3% and 37.1% in *TaGT‐2D*‐OE plants compared with normal light‐grown plants, significantly higher than 9.4% in negative plants (Figure 5h). By contrast, both of the two independent *TaGT‐2D*‐KO lines (59.6% and 86.2% leaves withered in *TaGT‐2D*‐KO226 and *TaGT‐2D*‐KO232 respectively) showed higher sensitivity to UV‐B stress than wild‐type plants (38.5% leaves withered), and only 0% to 7.4% plants survived after recovery, with 8.0% and 7.5% relative fresh weight compared to normal growth plants (Figure 5i,j). The *TaGT‐2D*‐OE leaves produced less H_2_O_2_, but *TaGT‐2D*‐KO plants accumulated more than wild‐type plants, as demonstrated by the NBT staining analysis (Figure 5k). MDA content was significantly lower in *TaGT‐2D*‐OE plants but higher in *TaGT‐2D*‐KO plants after UV‐B treatment (Figure 5l). These results indicated the *O*‐glucosyltransferase activity of TaGT‐2D in hexaploid wheat plants and demonstrated its positive role in UV‐B tolerance, suggesting that *TaGT‐2D* integrated from D‐genome to hexaploid wheat, increasing the content of glycosylated flavonoids and thereby enhancing UV‐B stress tolerance of hexaploid wheat.

## 3 Discussion

Allopolyploidization is caused by the merging of two or more genomes from divergent parental species in a common nucleus. This sudden perturbation results in broad genetic, epigenetic, and gene‐expression alterations [84, 85, 86]. Bread wheat is a typical allohexaploid plant that experienced two rounds of polyploidization events during its evolution [11, 12]. Integration of the D‐genome represents the second polyploidization event and played a vital role in facilitating the adaptation of wheat to diverse environmental conditions and cultivation areas [9, 87]. Here, we comprehensively reveal the impact of D‐genome integration from various aspects, including gene expression, epigenome organization, chromatin interaction and metabolites, correlating with enhanced adaptability of hexaploid wheat to salt, alkali, and UV‐B stress.

Hexaploid wheat rapidly surpassed its tetraploid ancestor species within a short period of time and became the most widely planted crop globally, accounting for more than 95% of all wheat‐growing areas, one likely reason is the improved environmental adaptability in hexaploid wheat [9]. Both our current study and other reports showed that hexaploid wheat is more tolerant to UV‐B light, alkaline stress, salt stress, nitrogen deficiency and zinc deficiency than tetraploid or diploid materials (Figure 1a–e and Figure S3) [19, 20, 21]. However, the mechanism of how adaptability increased in hexaploidy is yet to be clarified. Our studies revealed that the enhanced adaptability of hexaploid wheat is the result of a coordinated action at various aspects in wheat genome. The transcriptome analysis demonstrated the upregulation of many environmental adaptation or stress response genes during hexaploidization, both in the A, B and D subgenome (Figure 1i and Figure S6a). Epigenomic results suggested the enrichment of active histone modifications on different stress response genes (Figure 1m). Notably, gene transcription is governed by a multi‐layered network beyond epigenetics, and this biological complexity leads to the fact that a single or a few epigenetic features cannot achieve high correlation with gene expression (Figure 1l and Figure S10). Moreover, 3D interactome analysis revealed that lots of environmental adaptation genes were connected by more interaction loops following hexaploidization (Figures 2j and 3). Genes with higher differential interaction degree exhibit significantly higher expression than those with lower differential degree (Figure 2h,i), consistent with their higher absolute chromatin contact frequency (Figure S17b,i,j). While we did not observe a strict threshold effect and empirically adopted |differential degree| ≥ 3 for subsequent analyses, as this cutoff enriched for stress‐response pathways (Figure 2j). Furthermore, substantial enrichment of secondary metabolites related to stress responses have been observed in metabolome analysis (Figure 4). Genes correlated with increased secondary metabolites were also showed enhanced active histone modification and chromatin interactions, and increased transcription following D‐genome integration (Figures S26–S28). Nevertheless, we acknowledge that connection between a specific chromatin loop and a particular metabolite change remains phenomenological at this stage, and the establishment of a direct causal link would require targeted locus‐specific perturbations. We speculated that a tight connection of these adaptive genes in the wheat genome may facilitate their high‐efficiency co‐transcription, similar to the previously published “shepherd” model [53]. We indeed detected significant more loops connected to *TaRLK‐1B*, *TaRLK‐3B* or *TaCHI‐2B* genome region after hexaploidization, as well as genes associated with tolerance to different stresses such as high alkalinity, high salinity, UV‐B exposure, cold, low nitrogen, and flavonoid pathway genes, potentially contributing to transcription activation of these tightly connected genes (Figure 3a–f and Figure S20d,e). Functional validation demonstrated that *TaRLK‐1B* and *TaRLK‐3B* play positive roles in alkaline resistance of hexaploid wheat (Figure 3g–m). Therefore, hexaploidization may activate the transcription of these adaptation genes, accompanied by extensive remodeling of chromatin architecture, thereby enhancing the adaptive capacity of hexaploid wheat under environment stress. This association raises the possibility that chromatin loops contribute to the regulation of these genes, although establishing causality will require locus‐specific perturbation approaches, such as dCas9‐based loop engineering.

Hexaploidization event integrated novel genes to wheat genome from the DD progenitor [11], which facilitated the adaptability evolution of hexaploid wheat. We found the flavonoid pathway, which plays important roles in oxidation and UV resistance [35], was activated in the SHWs compared to their tetraploid parent (Figure 4l). This phenomenon possibly resulted from the addition of flavonoid synthesis genes located on the D subgenome (Figure 4m). Indeed, the putative function of one flavonoid pathway's glucosyltransferase encoded by D subgenome gene *TaGT‐2D* were confirmed in our present study (Figure 5). The content of three flavonoid glycosides increased when *TaGT‐2D* was overexpressed but decreased when *TaGT‐2D* lost its function, and dramatically changed the adaptive fitness of hexaploid wheat under UV‐B radiation (Figure 5g–l). This is correlated with the significant enhanced UV‐B tolerance of hexaploidy than its tetraploid parents (Figure 1c–e). SHWs also showed significant enhanced alkaline resistance than tetraploid parent (Figure 1b). These phenotypic data collectively indicate the importance of D genome resources in the environmental adaptability of hexaploid wheat during evolution and domestication. However, the integration of these elite genes from D‐genome into hexaploid wheat is unlikely to be the only reason, since hexaploid wheat exhibits slightly higher UV‐B tolerance than its DD parents, manifesting as less withered leaves in SHWs after UV‐B treatment (Figure 1d). The activation of other putative flavonoid pathway genes was also detected after D‐genome integration (Figure S25d), which may also enhance tolerance of hexaploids to UV‐B light. A significant higher salt tolerance of hexaploids compared to their DD parents and a slightly higher tolerance to its tetraploid parent was also demonstrated in this study (Figure S3b), indicating the interaction and activation of subgenome genes during D‐genome integration may be the main driving force of the enhanced salt tolerance. The L1 material, DD12, differed significantly from the two L2 materials (DD10 and DD11) in the principal component analysis of metabolome data (Figure 4a). There are also less ABD dominant metabolites detected in the ternary plot analysis of SHW5 compared to SHW3 and SHW4 (Figure 4d–f), implying the weaker activation at the metabolomic level during DD12‐genome integration process. In addition, DD12 was slightly more sensitive to salt stress and UV‐B radiation than DD10 and DD11 (Figure 1d and Figure S3b). Moreover, differences in resistance to UV‐B and salt stress between DD10 and DD11, both belonged to L2, were also detected in this study (Figure 1d and Figure S3b). The diverse performance of different D genomes at the metabolome level or under environmental stress highlights the potential for wheat adaptability breeding.

It has been reported that a large number of genes from the newly introduced D‐genome were suppressed during wheat polyploidization [32], which is in line with the observation here (Figure 1h). Interestingly, the reshaping occurred not only in the newly introduced D subgenome, but also affected the original A and B subgenomes spanning multiple levels (Figures 2g, 4j, S6 and S25d). The activation of B subgenome‐located alkaline tolerance gene *TaRLK‐1B* and *TaRLK‐3B*, associated with altered chromatin status and 3D interaction, confirmed the transcription regulation of original AB genome during D‐genome integration (Figure 3d–m). In addition, extensive interactions occurred among subgenomes during D‐genome integration were demonstrated. There are indeed massive newly established A–D and B–D interchromosomal interactions detected (Figure 2g), which enhanced the interactions and connections between subgenomes and hypothesized to substantially improve the genomic robustness, diversity and plasticity of gene transcription regulation in hexaploid wheat. Correspondingly, in our previous study, we have demonstrated the 3D chromatin interaction of *VRN1‐A*, *VRN1‐B*, and *VRN1‐D* locus when response to vernalization [53]. In this study, we found an enhanced transcription of BX biosynthesis genes, especially the B homoeologs, accompanied with activation of entire BX biosynthesis pathway in hexaploid wheat after hexaploidization (Figure 4j,k and Figure S23). BXs are widely reported to function in environmental adaptability [72, 73, 74, 88]. Considering the extremely low or lack of D homoeolog expression of key BX genes, we speculate a putative transcription element or activator in the D genome been introduced to hexaploid wheat and that this promotes the transcription of A, B subgenome located BX genes through subgenome interactions, resulting an over‐parental expression level of genes involved in BX pathway. However, whether both contribute and if so, what is the molecular hierarchy of these events requires further clarification in future studies.

The computationally constructed expected state should be interpreted as a parental‐derived reference rather than an exact quantitative reconstruction of the hexaploid molecular state. Although parental dosage‐based expectation are well established for transcriptomic analyses, chromatin‐associated measurements have distinct quantitative properties. Accordingly, the 1:1:1 representation of the A, B, and D subgenomes in the ChIP‐seq and ATAC‐seq pseudo‐datasets was used to establish a dosage‐matched parental reference without assuming strict biological additivity of chromatin enrichment or accessibility. For ChIA‐PET, parental datasets were processed independently and chromatin loops were identified separately before the AABB‐ and DD‐derived loop sets were integrated to construct the expected interaction repertoire. Thus, raw interaction frequencies were not directly added across parental datasets. Nevertheless, this computational framwork cannot capture *de novo* inter‐subgenomic interactions or higher‐order nuclear reorganization that emerges after the A, B, and D subgenomes coexist within the same nucleus. The concordant patterns observed in the cultivated hexaploid AK58 provide independent biological support for the major regulatory changes detected in SHW3 (Figure S18). Future analyses combining experimentally determined Hi‐C maps with in silico parental mixtures may further evaluate the robustness and boundaries of this parental‐reference framework.

**FIGURE 6:**
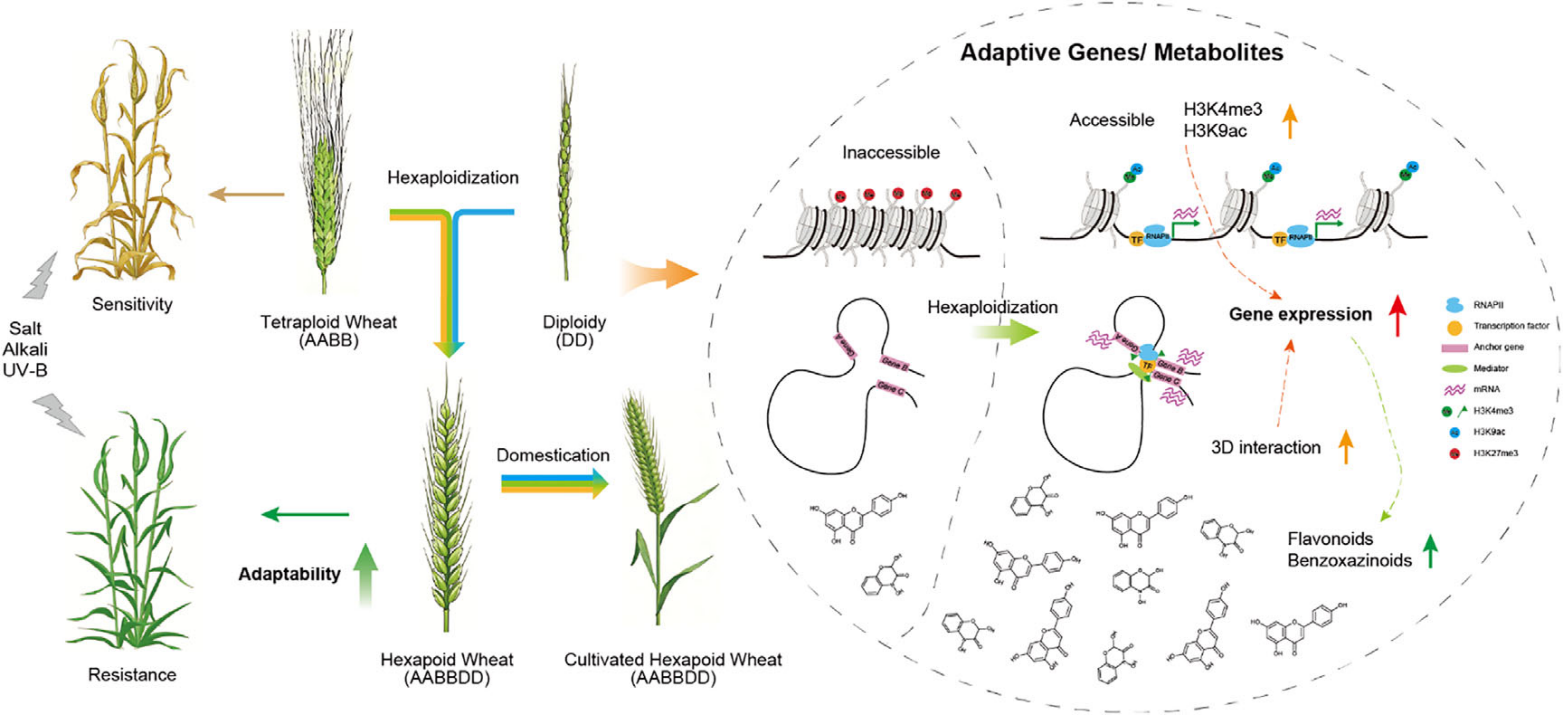
A schematic diagram showing the integration of D‐genome reshapes gene transcription, chromatin architecture and metabolome of hexaploid wheat leading enhanced adaptability to salt, UV‐B, and alkali stress.

In conclusion, we provide a comprehensive and systematic multi‐omics landscape resource, including the transcriptome, epigenome, 3D chromatin interactome, and metabolome, to understand the influence of D‐genome integration‐based hexaploidization events. Through integrated analysis of these atlases, together with genetic functional verification, we revealed that hexaploidization‐mediated dynamics in gene transcription, chromatin status, and metabolites increased the adaptive plasticity of allohexaploid wheat to salt, alkali, and UV‐B stress. In particular, genes and metabolites involved in environmental adaptability are significantly upregulated and enriched, associating with the coordinated regulation of the epigenome, chromatin accessibility, and chromatin interactome (Figure 6). However, the phenotypic evidence presented here is limited to salt, alkali, and UV‐B stress, the activation of other stress‐responsive genes represents promising candidates for future investigations. Our results shed light on the mechanistic basis of hexaploid wheat enhanced adaptability, which firstly results from functional genes integrated from D‐genome integration events (such as *TaGT‐2D*), and also derives from chromatin status regulation to activate transcription of adaptability genes located in the original A or B subgenomes (such as *TaRLK‐1B* and *TaRLK‐3B*). This coordinated interplay among subgenomes and the upregulation of protective small molecules likely explains the wide adaptation of allohexaploid wheat, which facilitated its worldwide spread and subsequently rendered it an indispensable global crop within a relatively short timeframe. Whilst our study provides considerable insight into this aspect of wheat evolution, we believe that the abundant multi‐omics data harvested here provides a key resource for polyploid crop adaptability breeding and offers candidate targets for improving resilience to climate change.

## 4 Experimental Section

### 4.1 Plant Materials, Growth Conditions and Stress Treatments

Three synthetic hexaploid wheat (SHW3, SHW4, SHW5; AABBDD), their related tetraploid durum wheat (AABB) and diploid *Ae. tauschii* (DD), and the hexaploid bread wheat Aikang 58 were used in this study. Detail information of these materials is provided in Table S1.

Materials used for stress treatments were soil‐grown in plastic pots under normal conditions for 18 days (three‐leaf stage) with 16 h light and 8 h dark photoperiod at 21°C ± 1°C in greenhouse. Each pot contained nine seedlings and served as a biological replicate. For alkaline stress, 100 mM mixed alkali (NaHCO_3_:Na_2_CO_3_ with a molar ratio of 5:1, 83.3 mM for NaHCO_3_ and 16.7 mM for Na_2_CO_3,_ respectively; pH 9.5) was used to treat the 18‐day‐old seedlings for 39 days. For salt treatment, 250 mM NaCl was used to irrigate these seedlings for 43 days. Another group of these materials continued to grow under normal conditions irrigating with water for control. For UV‐B stress, 18‐day‐old seedlings grown under normal conditions were transferred to a UV‐B chamber (TL8W/302 nm narrowband UV‐B tube) without white light. The UV‐B intensity was 12.8 µW cm^−2^ measured using a UV radiometer. After 9 days UV‐B stress, all the materials were transferred back to normal light conditions for recovery. One or Two weeks later, the growth and survival status of these plants were recorded.

For multi‐omics analysis, materials including SHWs, and its tetraploid and diploid parents were soil grown in greenhouse with 12 h light and 12 h dark cycle at constant temperature (16°C ± 1°C). The top fully expanded leaves of 21‐day‐old seedlings were harvested without crosslink and stored at −80°C for RNA sequencing (RNA‐seq) and metabolome analysis. Leaf samples for ChIP‐seq and ChIA‐PET experiments were cut into pieces for crosslinking immediately after being harvested. Samples for ATAC‐seq experiments were used fresh leaves without crosslinking.

### 4.2 RNA‐Seq Library Preparation

Total RNA were isolated from wheat leaves using TRIzol reagent (Invitrogen) according to protocol [89]. RNA libraries were constructed using MGIEasy RNA Library Prep Kit. RNA sequencing was performed using an MGI DNBseq‐T7 (paired‐end 150 bp reads) system. Three biological replicates of RNA‐seq data were obtained from each material.

### 4.3 ChIP‐Seq Library Preparation

ChIP‐seq experiments were performed according to published protocols [90, 91] with modifications. Wheat leaves were cut into pieces then cross‐linked with 1% formaldehyde in MC buffer [90] for 20 min and quenched using 1.25 M glycine (final concentration was 0.125 M). About 0.2 g cross‐linked tissues were ground into fine power in liquid nitrogen for each ChIP assay. Then, 300 µL Buffer S were added to lyse for 30 min at 4°C and 700 µL Buffer F for 15 min with rotation at 4°C. Chromatin were fragmented to 200–700 bp by sonication using Covaris S220 sonicator (peak power 150, percentage 6%, cycles per burst 200) for 10 min. After two rounds of centrifugation at maximum speed for 10 min at 4°C, supernatant containing sonicated chromatin were transferred to new tube for pre‐clearing rotation with 20 µL Dynabeads protein A (Invitrogen, 10002D) at 4°C for 1 h. Then another 40 µL Dynabeads protein A and 5 µL antibodies respectively against H3K4me3 (ABclonal, A2357), H3K9ac (Cell Signaling Technology, 9649S), H3K27me3 (Cell Signaling Technology, 9733S) were added to the chromatin supernatant for immunoprecipitation (IP) with rotation at 4°C overnight. After washed with low‐salt ChIP buffer, high‐salt ChIP buffer, ChIP wash/LiCl buffer and TE buffer in order, the protein‐DNA complexes were eluted from Dynabeads protein A using ChIP elution buffer by two rounds at 65°C for 15 min with agitation at 900 rpm. 15 µL 5 M NaCl and 13 µL proteinase K (20 mg/mL) were added to the eluate for reverse crosslinking at 65°C for 8 h. ChIPed DNA were precipitated by pre‐cooled ethanol and purified using QIAquick PCR Purification Kit (QIAGEN, 28106), then resolved with 25 µL Buffer EB. ChIP‐seq libraries were prepared using ThruPLEX DNA‐seq 48S Kit (Takara, R400427) following the manufacturer and sequenced using Illumina NovaSeq 6000 system (paired‐end 150 bp reads). The ChIP‐qPCR were conducted using ChIPed DNA as template with primers listed in Table S29, and the input DNA was used as negative control.

### 4.4 ATAC‐Seq Library Preparation

To construct ATAC‐seq libraries, fresh tissues were cut into pieces and chopped into homogenate in pre‐chilled D‐Sorbitol buffer (0.6 M D‐sorbitol, 20 mM MES, 20 mM KCl, 10 mM MgCl_2_, 1 × PMSF, 0.2% Triton X‐100, 1 mM β‐mercaptoethanol, 0.1 mg/mL BSA, 1 × protease inhibitor) [92]. Then, the homogenate was filtered through 30 µm cell strainer twice. After stained with 4',6‐diamidino‐2‐phenylindole (DAPI), crude nuclei were loaded into a flow cytometer (BD FacsAria II SORP). A total of 50,000 nuclei were sorted and collected in pre‐chilled D‐Sorbitol buffer, then the nuclei were spun down at 1000 g for 10 min at 4°C. After supernatant removed carefully, nuclei were mixed with 1 × TD buffer (10 mM Tris‐HCl, 5 mM MgCl_2_, 10% DMF) and 1.5 µL TTE Mix (Vazyme, TD501) and incubated at 37°C for 30 min with mixing gently every 5 min. Reactions were stopped using Buffer PB, and DNA were purified by MinElute PCR Purification Kit (QIAGEN, 28006). ATAC‐seq libraries were prepared with NEBNext High‐Fidelity 2X PCR Master Mix (NEB, M0541L) and TruePrep Index Kit V2 for Illumina (Vazyme, TD202). Libraries were sequenced using Illumina NovaSeq 6000 system (paired‐end 150 bp reads).

### 4.5 Long‐Read ChIA‐PET Library Preparation

H3K4me3‐associated long‐read ChIA‐PET library construction were performed according to published protocol with minor modifications [55, 58, 93]. Briefly, fresh wheat leaves were cut into pieces and treated with 1.5 mM Ethylene glycol bis (succinimidylsuccinate) (EGS, Thermo Scientific, 21565) under vacuum for 20 min crosslinking. Then formaldehyde (Thermo Scientific, 28906) was added to 1% final concentration for another 20 min crosslinking. One tenth volume of 1.25 M glycine were added to stop the crosslinking reaction, and washed three times with ddH_2_O. Cross‐linked tissues were stored at ‐80°C until use. About 4.5 g sample was used for each ChIA‐PET experiment. After ground into fine powder in liquid nitrogen, tissues were mixed thoroughly with 6.75 mL 1% SDS FA nuclear lysis buffer and incubated at 4°C for 30 min with rotation and 27 mL SDS‐free FA lysis buffer for another 15 min incubation at 4°C. The solution was sonicated to fragment chromatin into 1–3 kb using a BioRuptor (Diagenode) under high level intensity (30 s ON and 50 sec OFF, 20 cycles). After centrifugation at 12 000 rpm for 10 min at 4°C, the supernatant was transferred to new tube for next immunoprecipitation. About 800 µL Dynabeads protein A were mixed with 100 µL H3K4me3 antibody (ABclonal, A2357) and incubated at 4°C for 8 h to generate antibody‐coated beads. Then, the fragmented chromatin was incubated with antibody‐coated beads at 4°C overnight with rotation. The ChIPed beads were orderly washed with 5 mL 0.1% SDS FA cell lysis buffer three times, 5 mL High salt ChIP buffer twice, 5 mL ChIP wash buffer once and 5 mL TE buffer once. After end‐repairing and A‐tailing, biotinylated bridge linker was used to perform proximity ligation of ChIP DNA on the beads. The proximity ligated ChIP DNA were reverse cross‐linked using proteinase K and extracted by phenol‐chloroform alcohol. ChIA‐PET libraries were constructed using Tn5 transposase (TruePrep DNA Library Prep Kit V2 for Illumina, Vazyme, TD501) and sequenced using Illumina NovaSeq 6000 system (paired‐end 150 bp reads).

### 4.6 Metabolic Profiling and Metabolite Annotation

Metabolite extraction and detection was performed according to previous research [71]. The freeze‐dried wheat leaves were pulverized by a mixer mill at 30 Hz for 1 min. 100 mg powder was used to extract metabolites for each biological replicates by adding 1 mL pre‐cooled 70% methanol (containing 0.1 mg/L acyclovir as internal standard). After mixing thoroughly, the homogenate was incubated in an ultra‐sonication bath for 15 min at 4°C with vortexing every 3 min or incubated at 4°C with rotation overnight, then followed centrifugation at 12000* g* at 4°C for 10 min. The supernatant was filtered through a 0.22 µm filter membrane (ANPEL, SCAA‐104). The final filtrates were analyzed using liquid chromatography‐electrospray ionization‐ tandem mass spectrometry (LC‐ESI‐MS/MS) system. The MS2T library was established using a stepwise multiple ion monitoring‐enhanced product ion according to previous published protocols [71]. To facilitate annotation of metabolites, the detailed information contained in metabolite signals were compared with metabolite databases and available commercial standards. The scheduled multiple reaction monitoring (MRM) method was used to quantified the relative contents of the 839 metabolites identified in this study as previously described [94]. The scheduled MRM algorithm was used with an MRM detection window of 90 sec and a target scan time of 1 sec using Analyst 1.5 software. Metabolite data are reported following set reporting standards [95, 96].

### 4.7 RNA‐Seq Data Analysis

RNA‐seq reads were aligned to the Chinese Spring RefSeqv1.0 reference genome [11] using STAR [97] (version 2.7.1a). Materials of different ploidy were individually mapped to the corresponding genomes, the tetraploid datasets of AABB were only aligned to A and B subgenome transcripts and the diploid of DD was only aligned to D subgenome transcripts, and the hexaploidy of AABBDD was aligned to the A, B, and D subgenome transcripts. The expression of annotated genes was measured by RSEM [98] (version 1.2.22) and normalized with transcripts per million (TPM). We considered the gene with TPM > 0.5 expressed. For gene quantification comparison, we referred to published research [52] and combined the TPM matrices of AABB and DD in a 2:1 ratio and then normalized them to 10^6^ as SHW to generate “expected” dataset. We consider a gene as differentially expressed gene (DEG) when: 1. TPM in expected and observed >0.5, fold change >2 and FDR <0.05; or 2. TPM in expected = 0 and observed >0.5, or expected >0.5 and observed = 0; or 3. TPM in 0< expected < 0.5 and observed > 1, or expected > 1 and 0< observed < 0.5. For expression breadth analysis, we downloaded the transcription data of 23 different tissues from published research [52] (Table S12, 3 replicates were included), and genes with TPM > 1.5 were considered as expressed in this tissue.

### 4.8 Homoeolog Expression Bias Analysis

We first downloaded gene triads sets with A: B: D = 1:1:1 correspondence in three homologous subgenomes [52]. We extracted these gene TPM values from all genes’ TPM matrix generated above and normalized the TPM for each gene within the triad according to the published method [52]. The TPM sum of the gene triad below 0.5 was excluded from the downstream analysis. Then we calculated the relative contributions of these genes in the respective triad and calculated the Euclidean distance to the ideal normalized expression bias with the rdist function from rdist R package (v0.0.5) [52]. We group each triad into the same category as the one with the shortest distance from the seven ideal categories.

### 4.9 GO Analysis

The gene ontology (GO) enrichment analyses were performed on the Triticeae‐Gene Tribe database [99] (http://wheat.cau.edu.cn/TGT/). The GO term with a Bonferroni corrected *p* value less than 0.05 we considered as significant.

### 4.10 ATAC‐Seq Data Analysis

For the pre‐processing of ATAC‐seq data, we follow the workflow of ChIP‐Hub and cisDynet [100, 101]. The raw reads were first trimmed by Trimmomatic [102] (version 0.36) to remove sequencing adapters. The trimmed reads were aligned to the Chinese Spring RefSeqv1.0 reference genome [11] using Bowtie2 [103] with the following parameters “‐q—no‐unal—threads 8—sensitive”. All mapped to mitochondrial and chloroplast DNA reads were removed. After sorting mapped reads with SAMtools [104] (version 0.1.19), we only used properly paired reads with high mapping quality (MAPQ score > 30) for the subsequent analysis. The PCR duplicates were removed using the MarkDuplicates function from Picard tools (version 2.60; http://broadinstitute.github.io/picard/). MACS2 [105] (version 2.1.0) was used to call peaks. Using the “callpeak” function in MACS2 with the following parameters: “–call‐summits ‐f BAM –nomodel –mfold 2 20 –qvalue 0.01”. The “‐shift” used in the model was determined by the analysis of cross‐correlation scores using the phantompeakqualtools package (https://code.google.com/archive/p/phantompeakqualtools/). The “–gsize” was set to the corresponding subgenome size. The final peaks were reproducible across pseudo‐replicates and true replicates with an IDR (Irreproducibility Discovery Rate) < 0.05. For quantification, the Tn5 insertion positions were first determined as the start sites of reads adjusted by the rule of “forward strand +4 bp, negative strand −5 bp” [106]. The peak sets were merged by the merge function from BEDTools [107] (version 2.30.0), the number of Tn5 cuts in each peak was counted, and the sum is normalized to 10^6^ by dividing by the length of the peaks.

### 4.11 ChIP‐Seq Data Analysis

We used the ChIP‐Hub [100] analysis workflow to process the ChIP‐seq data. The sequencing data trimming, reads alignment, filtering, etc. were all processed in the same manner as the ATAC‐seq. The peak calling was also similar to that of ATAC‐seq, except that the input sample was added here as a control.

### 4.12 Differential Peak identification

We merged the peaks of H3K9ac, H3K27me3, H3K4me3, and ATAiC‐seq separately. For the histone modification data, we counted the number of reads in the corresponding peak and divide it by the length of the peak, and finally normalized it to 10^6^ (Counts Per Million, CPM). For ATAC‐seq, we counted the insertion position of Tn5 as described above. Then we used the Shannon entropy method to screen differential peaks. We calculated the entropy score for each peak using the philentropy (https://github.com/drostlab/philentropy) R package and considered the entropy value less than 1.6 as differential peaks. Finally, we performed Z‐score transformation by row for visualization. For the target genes of these differential peaks, we assigned these peaks to the nearest TSSs according to their summit positions using BEDTools [107] closest function to identify target genes. Based on the region of the peak summit on the genome, we divide them into intragenic, promoter, and distal regions. The GO enrichment of the target gene was performed as described above.

### 4.13 Pseudo‐Bam Generation and Genome Tracks Visualization

To construct a genome‐dosage‐matched parental reference for comparison with the hexaploid genome, we generated pseudo‐BAM files in which reads derived from the A, B, and D subgenomes were represented at a 1:1:1 ratio, corresponding to their relative genomic dosage in AABBDD wheat. Then we used the bamCoverage function from deepTools [108] (version 3.5.1) package with the following parameters “–normalizeUsing RPKM –binSize 5” to generate bigWig file. We used the WashU Epigenome Browser [109] to visualize signal tracks with bigWig files. This computational reference was used to represent the parental‐derived expectation and does not assume strict biological additivity of chromatin enrichment or accessibility among subgenomes.

### 4.14 ChIA‐PET Data Analysis

The ChIA‐PET data were analyzed using updated ChIA‐PET Tool software package for automatic processing of sequence data [110], including linker filtering, read mapping to reference genome [11] using BWA, redundancy removal, identification of protein binding sites and chromatin interactions. Mapped PETs were classified to self‐ligation, inter‐ligation and other PETs according to genomic span of a PET. Obtained inter‐ligation PETs (genomic span >8 kb) were used to generate raw contact frequency matrix and other future analysis. Considering the large genome size and intergenic regions of wheat, we chose ChIP‐seq peak summit ± 3 kb as the given anchors to call clusters with removal of overlapping with adjacent anchor region. We defined a loop as PPI interaction when the two ChIP‐seq peak summit all located in promoter regions, and as PDI interaction when one peak summit located in promoter regions and another peak summit located in distal regions (regions outside promoter and gene body). We also defined a loop as intrachromosomal loop when the two anchors located in the same chromosome and as interchromosomal loop when the two anchors located in different chromosomes. For construction of the expected hexaploid chromatin‐interaction dataset, ChIA‐PET data from the tetraploid AABB and diploid DD progenitors were first independently mapped to their corresponding reference genomes and processed separately for chromatin‐loop identification. The independently identified AABB‐ and DD‐derived loops were subsequently integrated to generate the expected parental interaction repertoire. Thus, the expected ChIA‐PET dataset was constructed at the loop level rather than by directly mixing raw PETs or linearly summing parental interaction frequencies.

### 4.15 Network Construction

The interaction networks were constructed through two or three hops of interactions (including PPI and PDI) originating from these selected core DEGs or adaptive genes. Nodes, including anchor genes and distal elements, were connected on the basis of chromatin interactions present in the ChIA‐PET libraries from SHW3 (after polyploidization) and its tetraploid, diploid parents (before polyploidization) respectively. The connectivity networks were visualized and modularized using Gephi [111].

### 4.16 Generation of Transgenic Plants

For generation of overexpression transgenic lines, the full‐length cDNA of *TaRLK‐1B* (*TraesCS1B02G022400*) and *TaRLK‐3B* (*TraesCS3B02G056100*) were isolated from allohexaploid wheat variety China Spring (CS), and full‐length cDNA of *TaGT‐2D* (*TraesCS2D02G069100*) was isolated from SHW3. Both of these three genes were recombined into p203159 [112] vector under maize ubiquitin promoter. To generate loss‐of‐function mutants of *TaGT‐2D*, two gRNAs specific targeting the coding region of *TaGT‐2D* were designed (Figure 5b), and CRISPR‐Cas9 vector p203165 [112] was used to express the gRNAs and Cas9. Immature embryos of Fielder were used for wheat transformation with *Agrobacterium tumefaciens* strain EHA105 carrying the constructs [112]. The positive overexpression transgenic plants were identified using vector‐targeted primer and gene‐specific primers. The genotype mutation created by CRISPR‐Csa9 were identified using gene‐specific primers. All the primers used in this study for construction and identification are listed in Table S29.

### 4.17 NBT Staining

The NBT staining analysis was performed following published methods [113]. Briefly, wheat leaves were vacuum‐immersed in a 10 mmol/L phosphate buffer solution (pH 7.6) containing 0.1% nitroblue tetrazolium (Beyotime, Y264343) and 10 mmol/L sodium azide. Following a 24‐hour incubation in the dark at 25°C, chlorophyll was removed by transferring stained samples to 95% ethanol and incubating at 80°C for 15 min.

### 4.18 MDA Content Determination

Quantitative analysis of MDA content in wheat leaves was performed using an MDA detection kit (Beyotime, S0131S). Weigh 0.02 g of wheat leaf samples and homogenize in 200 µL of 10% trichloroacetic acid (TCA). Centrifuge at 12000 rpm for 10 min at room temperature. Mix 100 µL supernatant with 200 µL MDA detection working solution. Heat the mixture at 100°C for 15 min, immediately cool on ice, then centrifuge again (12 000 rpm, 10 min, room temperature). Measure absorbance of the centrifuged supernatant at 532 nm wavelength. Calculate the sample MDA concentration by plotting a standard curve based on the absorbance values of the standard samples.

### 4.19 Chromosome Conformation Capture (3C) Assays

The 3C assay was performed with minor modifications to the previous protocol [114]. Briefly, approximately 3 g of wheat leaf tissue was fixed in 1% formaldehyde, and nuclei were isolated by grinding the fixed tissue to a fine powder in liquid nitrogen. Chromatin within the isolated nuclei was digested with 400 units (U) of the restriction endonuclease HindIII (New England Biolabs, R3104S). Subsequent ligation of the digested DNA fragments was carried out using T4 DNA ligase (New England Biolabs, M0202). The ligated DNA products were purified and used as templates for 3C‐quantitative real‐time PCR (3C‐qPCR) analysis. Randomly ligated (RL) libraries were constructed from genomic DNA extracted from uncrosslinked wheat leaf tissue. A total of 10 µg of the extracted genomic DNA was subjected to identical HindIII digestion and T4 DNA ligase‐mediated ligation steps to generate the RL library. Specific forward primers were designed to target restriction sites within four predicted chromatin interaction loops, as well as endogenous reference detection sites. All 3C‐qPCR reactions were set up with 150 ng of ligated DNA template per reaction system. The calculation of chromatin interaction frequencies was performed as previously reported [114]. The nucleotide sequences of all primers used in this assay are provided in Table S29.

### 4.20 Code Availability

The data analysis code has been stored as Jupyter notebooks in the GitHub repository (https://github.com/tzhu‐bio/Wheat_Polyploidization_Analysis).

## Author Contributions

W.Y. conceived the study with input from D.C. and A.R.F.; Y.L. conducted most of experiments and generated the data with assistance from W.C. X.W., C.C., B.Y., S.B., W.O., Q.L., H.M., J.W., M.L., and X.L.; D.C., T.Z., and Y.L. performed data analysis with support from X.Z., C.H., W.C. X.H., and S.B.; Z.Z. performed the transgenic plants generation. M.K. and C.L. provided the materials. H.S. performed cytology karyotyping analysis. Y.L. and T.Z. drafted the manuscript. W.Y., D.C., A.R.F., and K.K. discussed and revised the manuscript.

## Conflicts of Interest

The authors declare no conflicts of interest.

## Supporting information




**Supporting File 1**: advs77894‐sup‐0001‐SuppMat.pdf.

**Supporting File 2**: advs77894‐sup‐0002‐SuppMat.zip.

## Data Availability

All omics data supporting the findings of this study are openly available. The RNA‐seq, ATAC‐seq, ChIP‐seq and ChIA‐PET data generated in this study were deposited into the NCBI BioProject under the accession number PRJNA939846 (https://www.ncbi.nlm.nih.gov/bioproject/?term=PRJNA939846). Metabolomics data harvested in this study have been deposited in the OMIX, China National Center for Bioinformation */*Beijing Institute of Genomics, Chinese Academy of Sciences under the accession number OMIX015317 (https://ngdc.cncb.ac.cn/omix/release/OMIX015317). The RNA‐seq and ChIA‐PET data of hexaploid variety AK58 were obtained from the Genome Sequence Archive (GSA) at the Beijing Institute of Genomics (BIG) Data Center, Chinese Academy of Sciences, under accession number CRA015030 (https://ngdc.cncb.ac.cn/gsa/search?searchTerm=CRA015030). The transcriptome data of other cultivated hexaploid wheat were downloaded from the WheatOmics database (https://wheatomics.sdau.edu.cn/download.html).
