# Supplementary material for: Integration of the D‐Genome Reshapes Gene Transcription, Chromatin Architecture, and Metabolome of Allohexaploid Wheat, Leading to Enhanced Environmental Adaptability
Source: Adv Sci (Weinh). 2026 Sep 27:e77894. Online ahead of print. doi: 10.1002/advs.77894 (PMC13616400; doi:10.1002/advs.77894)
Supplement: Supplementary file 1 [file ADVS-9999-e77894-s001.pdf]

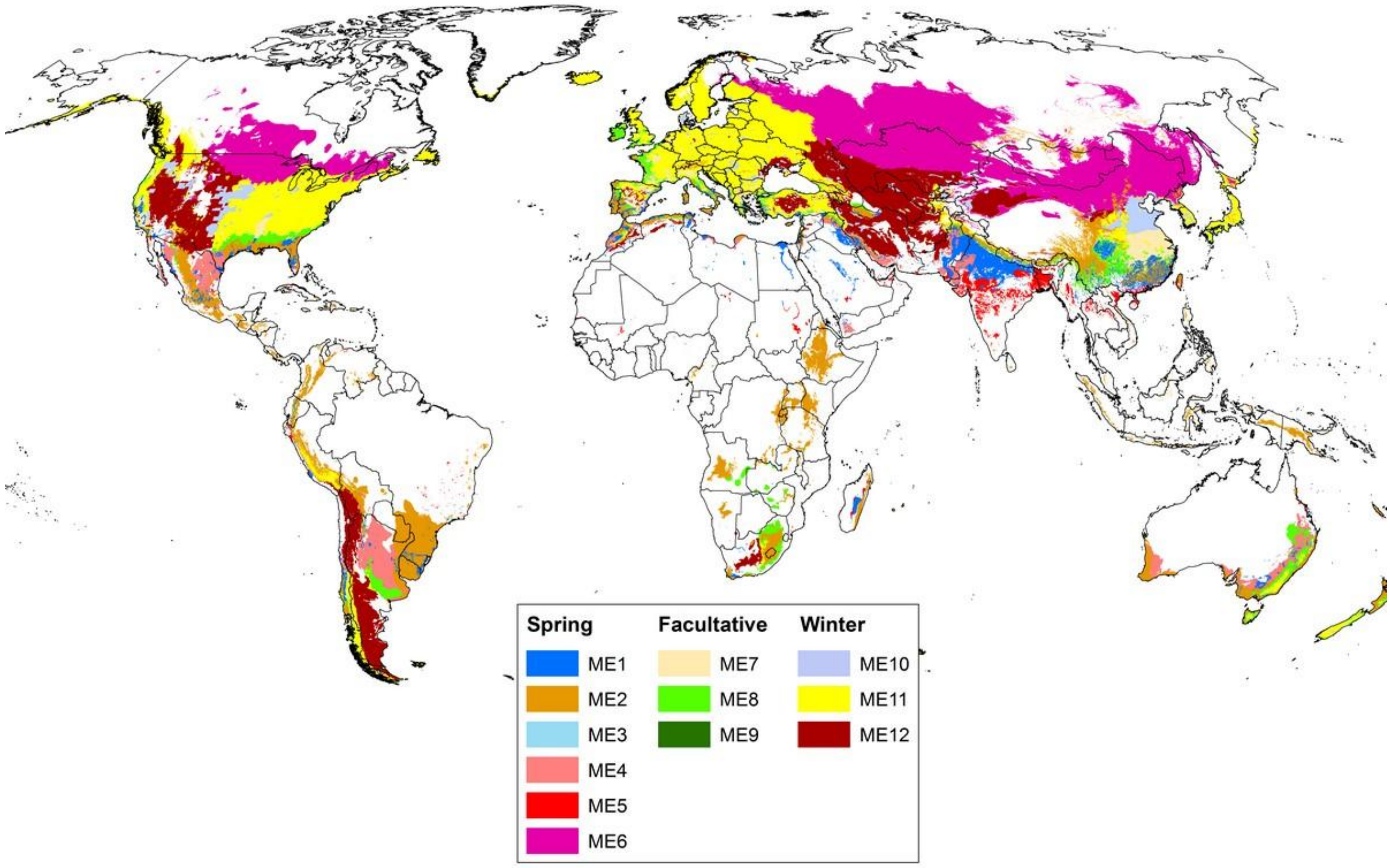

Figure S1. Global Distribution of Wheat Megaenvironments. Different colors represent different mega-environments (ME) that showed different biotic and abiotic stresses and cropping systems. ME1: low rainfall, irrigated, temperature (T) for coolest quarter 11°C > T ≥ 3°C. Major constraints: leaf rust, stem rust, stripe rust, lodging. ME2: high rainfall, temperature for coolest quarter 16°C > T ≥ 3°C. Major constraints: barley yellow dwarf virus, stripe rust, head scab, septoria, tan spot. ME3: high rainfall, temperature for coolest quarter 16°C > T ≥ 3°C, soil pH < 5.2. Major constraints: barley yellow dwarf virus, stripe rust, head scab, septoria, tan spot, acid soil (Aluminum, Manganese toxicity). ME4: low rainfall, temperature for coolest quarter 16°C > T ≥ 3°C. Major constraints: drought, rust, common bunt, septoria, fusarium, tan spot, root diseases. ME5: tropical high rainfall, and/or irrigated, temperature for coolest quarter 16°C > T > 11°C. Major constraints: heat, spot blotch, leaf & stem rust. ME6: high latitude (>45° N or S), temperature for coolest quarter T < -13°C and for warmest quarter T = 9°C. Major constraints: rust, root diseases, tan spot. ME7: irrigated, temperature for coolest quarter 3°C > T ≥ -2°C. Major constraints: cold, stripe rust, mildew. ME8: high rainfall/irrigated, temperature for coolest quarter 6°C > T ≥ 1°C. Major constraints: cold, stripe rust, mildew, septoria, root rots. ME9: low rainfall, temperature for coolest quarter 3°C > T ≥ -2°C. Major constraints: cold, drought, stripe rust, root rots. ME10: irrigated, temperature for coolest quarter -2°C > T ≥ -13°C. Major constraints: winter kill, rust, mildew. ME11: high rainfall/irrigated, temperature for coolest quarter 1°C > T ≥ -13°C. Major constraints: winter kill, rust, septoria, mildew. ME12: low rainfall, temperature for coolest quarter 1°C > T ≥ -13°C. Major constraints: winter kill, drought, stripe rust, bunts, root rots. The data were downloaded from website http://wheatatlas.org/me-global-distribution.

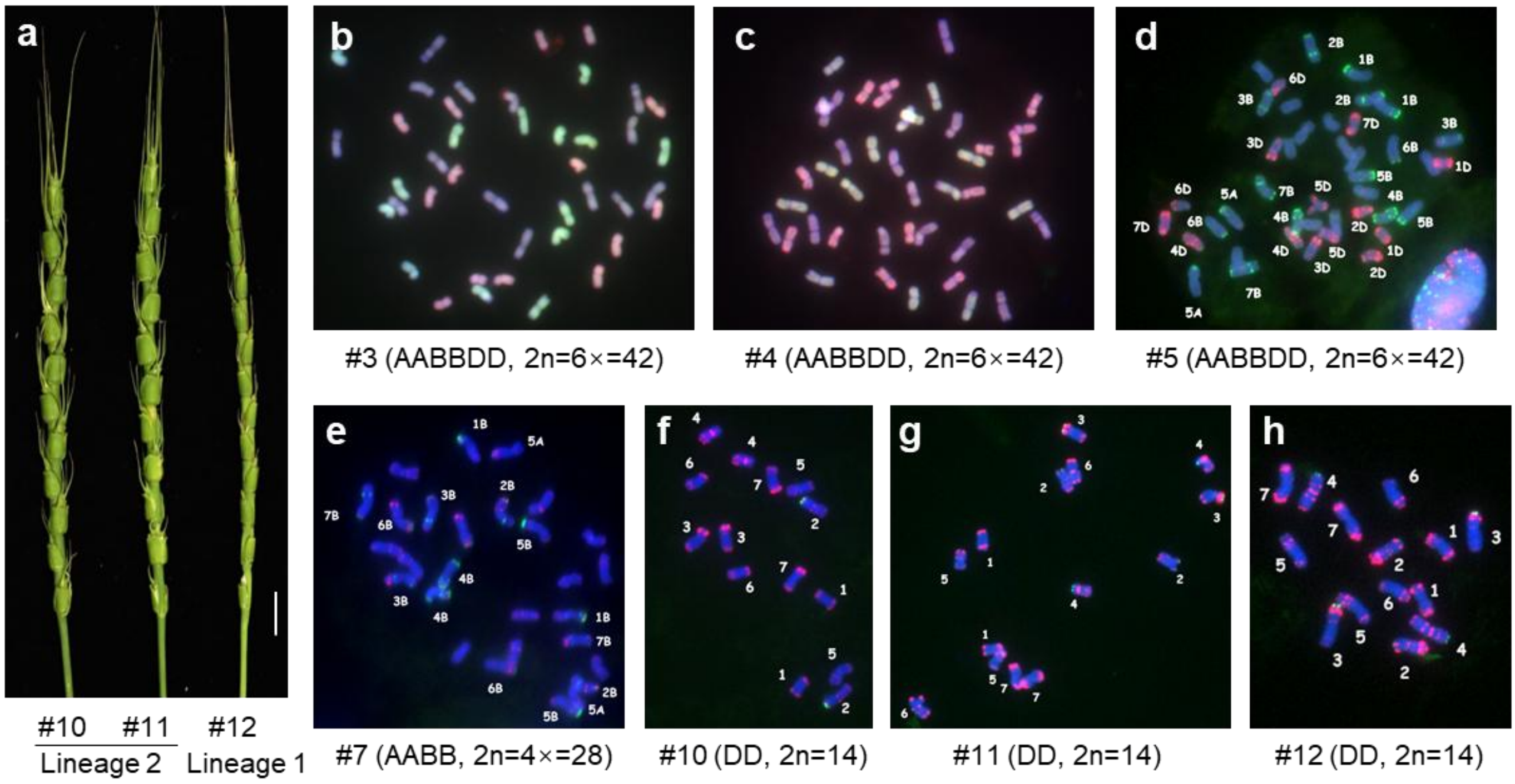

Figure S2. Morphological characteristics of the diploid *Ae. tauschii* and cytology karyotyping analysis of materials used in this study. a, Morphological characteristics of spikes of the diploid parents used in this study. Bar = 1 cm. **b, c,** Genomic in situ hybridization of mitotic cells in the root tip from allohexaploid SHW3 (**b**), SHW4 (**c**) colored by the subgenome A (green), B (blue), D (pink). **d-h,** Fluorescent in situ hybridization using mitotic cells of the root tip from allohexaploid SHW5 (**d**), tetraploid parent *T. turgidum* (**e**) and diploid parents *Ae. tauschii* (**f-h**) using pAS1-1 (red) and 119.2 (green) as probes.

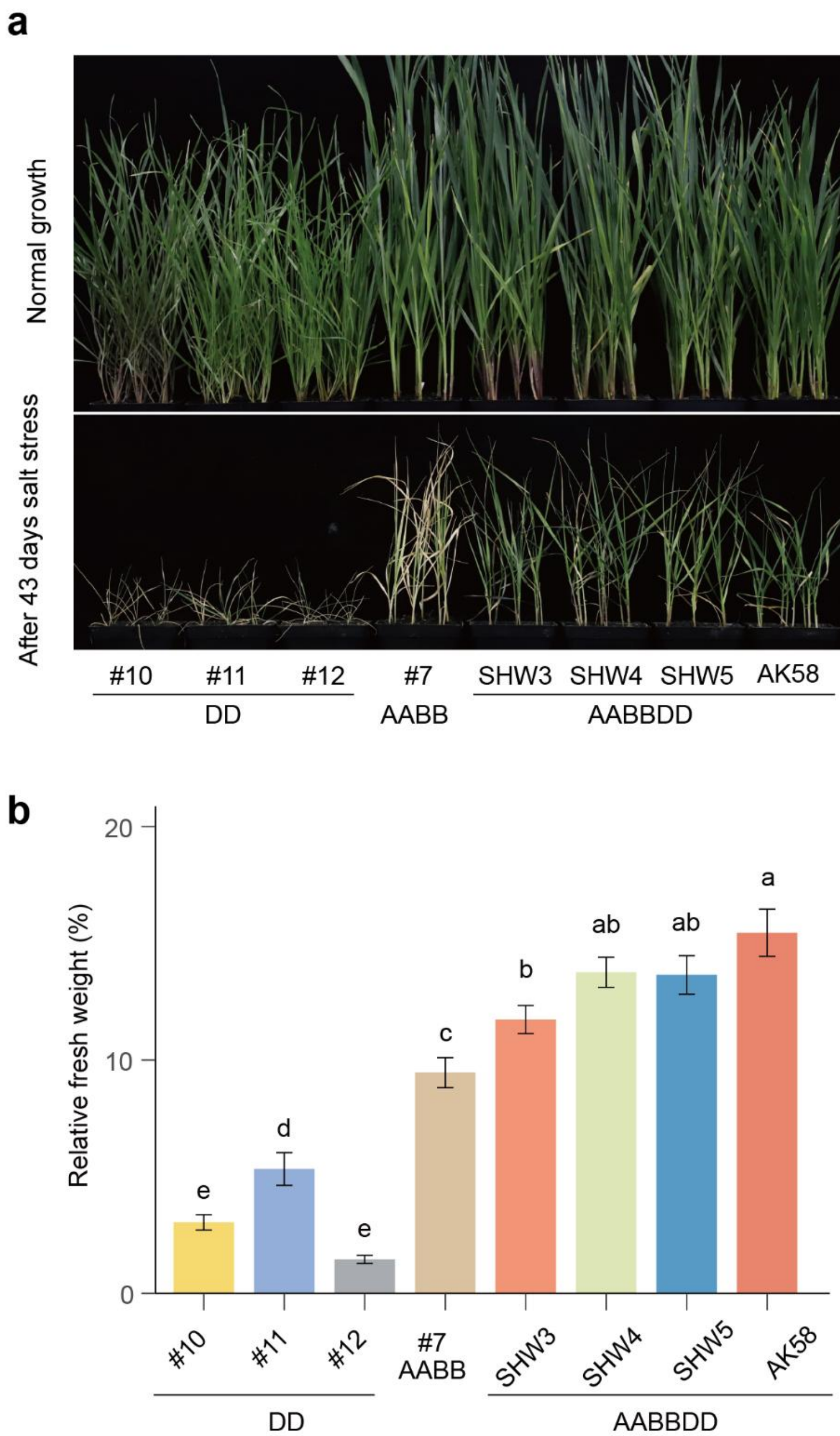

Figure S3. Phenotypes of allohexaploid AK58, SHWs (AABBDD), and tetraploid (AABB) and diploid (DD) parents under salt stress. a, Enhanced tolerance of allohexaploid wheat under salt stress compared with its tetraploid and diploid parents. Photographs were taken after 43-day salt solution treatment. **b**, Statistical analysis of the relative fresh weight of seedlings under 43-day salt stress. Different lowercase letters above bars indicate significant differences among materials at $P < 0.05$ according to Student-Newman-Keuls test for multiple comparisons.

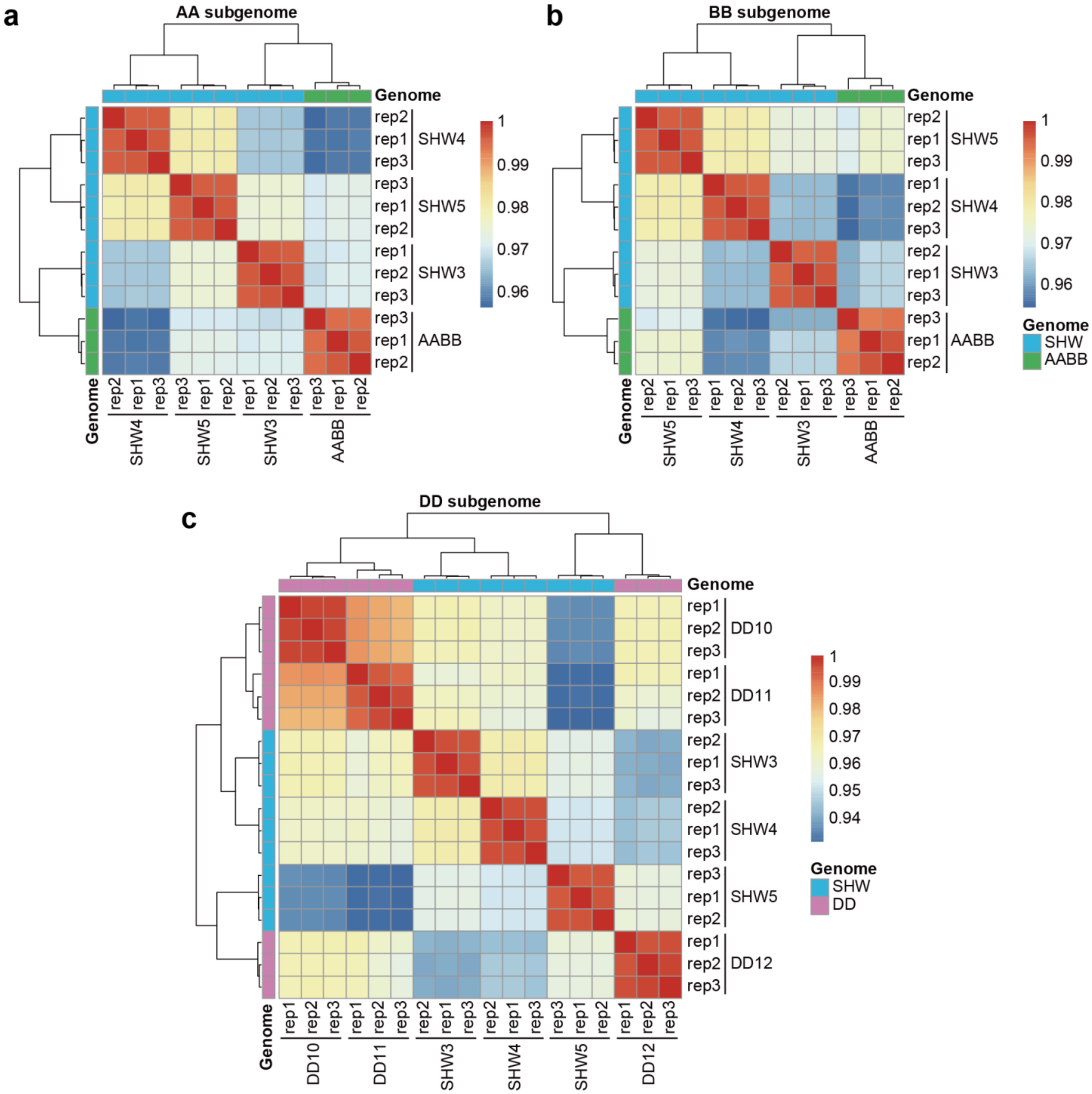

Figure S4. Reproducibility analysis of transcriptome data in this study. a, Correlation coefficients of A subgenome transcriptome data from materials containing A genome. **b,** Correlation coefficients of B subgenome transcriptome data from materials containing B genome. **c,** Correlation coefficients of D subgenome transcriptome data from materials containing D genome.

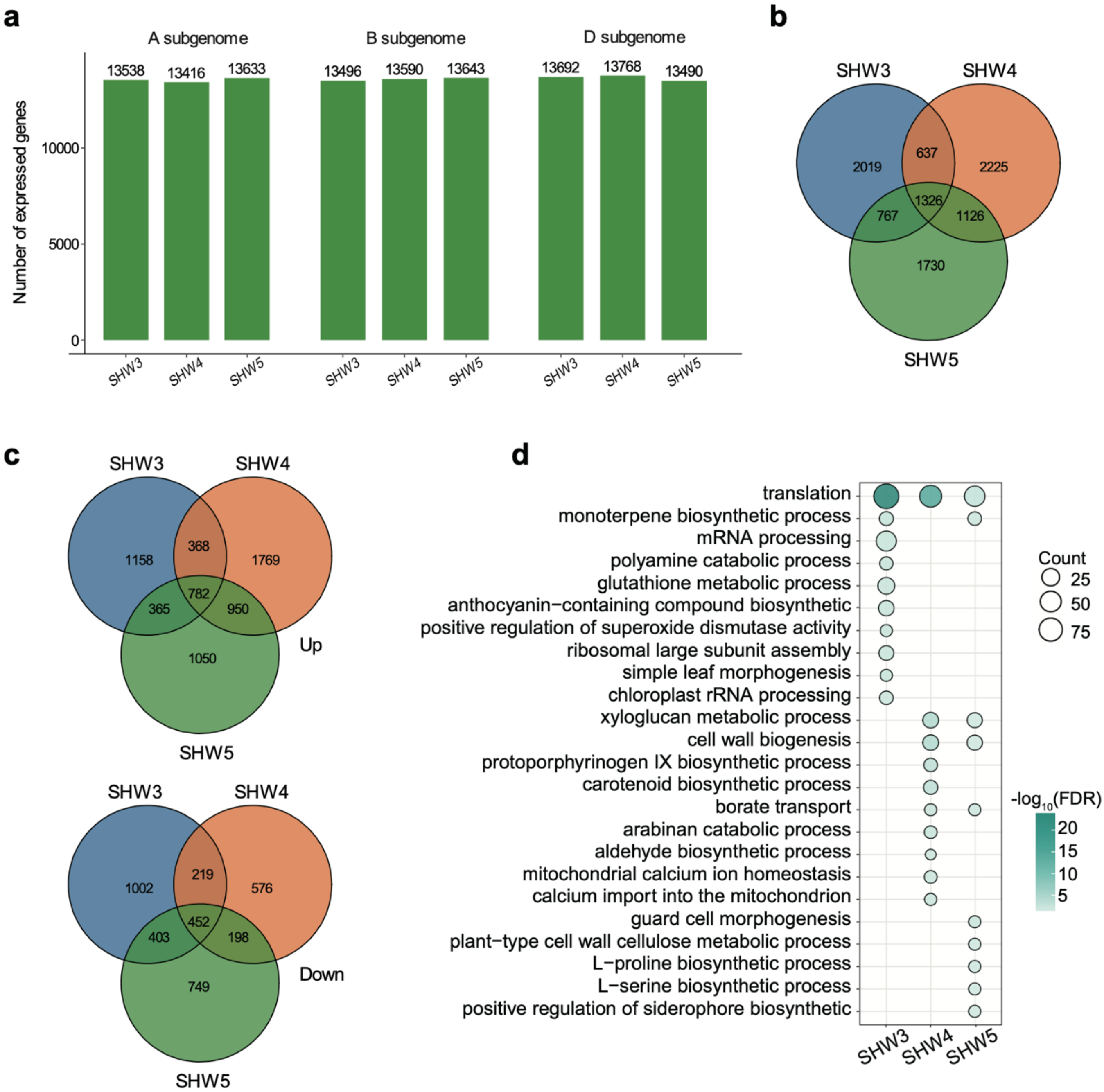

Figure S5. Gene expression analysis. a, The total number of expressed genes in SHW3, SHW4 and SHW5. Genes were considered to be expressed only when their expression level tpm > 0.5. **b,** Venn diagrams showing the overlap of DEGs in SHW3, SHW4 and SHW5. **c,** Venn diagrams showing the overlap of activated genes (top) and suppressed genes (bottom) in SHW3, SHW4 and SHW5. **d,** Top ten GO terms enriched in down-regulated DEGs. Dot size represents the gene number enriched in GO terms and color gradation represents the significance of enrichment as $-\log_{10}$ (FDR).

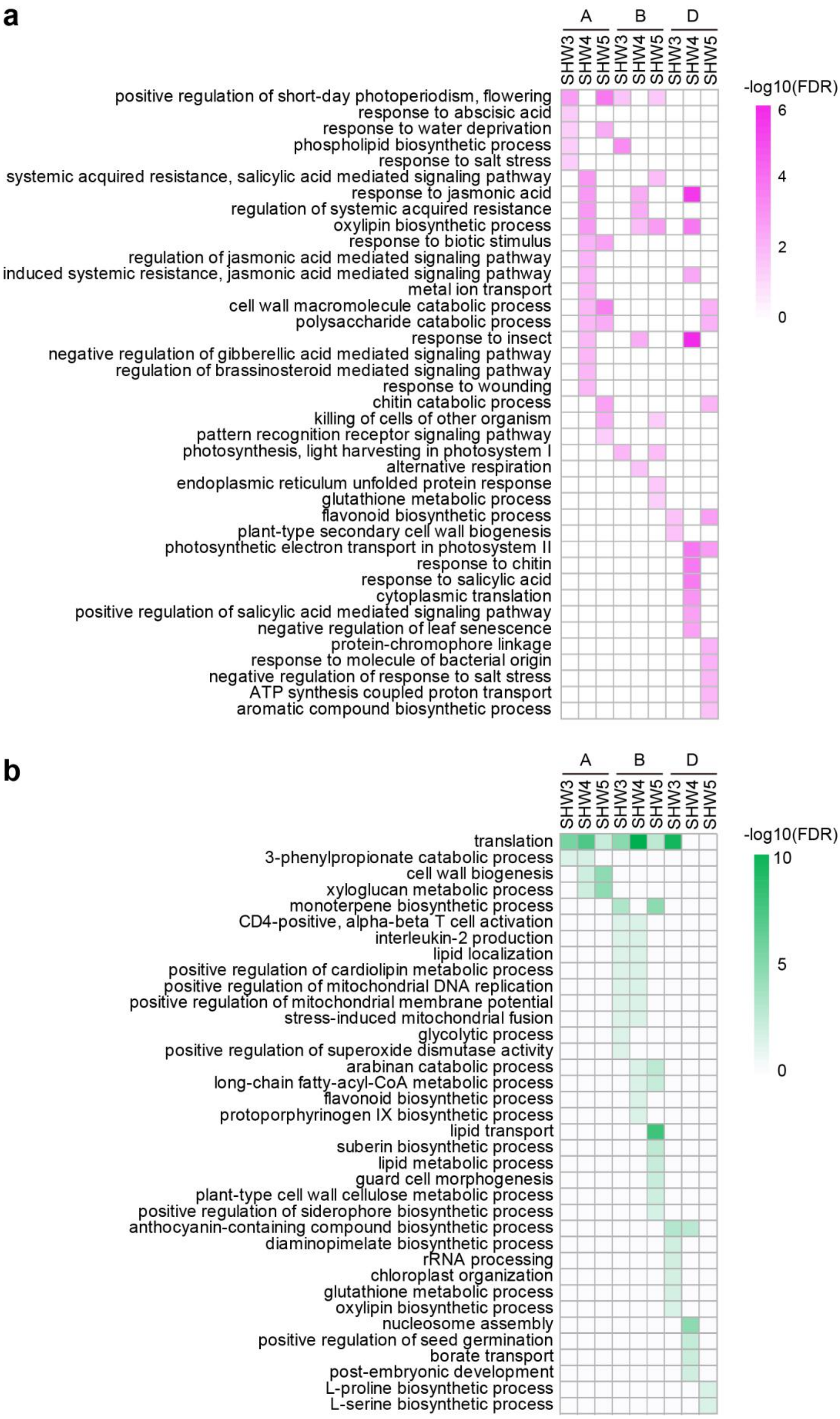

Figure S6. GO enrichment analysis for DEGs in different subgenomes. a, Enriched biological process GO terms for up-regulated DEGs in SHW3, SHW4 and SHW5. **b,** Enriched biological process GO terms for down-regulated DEGs in SHW3, SHW4 and SHW5. The gradation of color represents the significance of GO terms as $-\log_{10}$ (FDR).

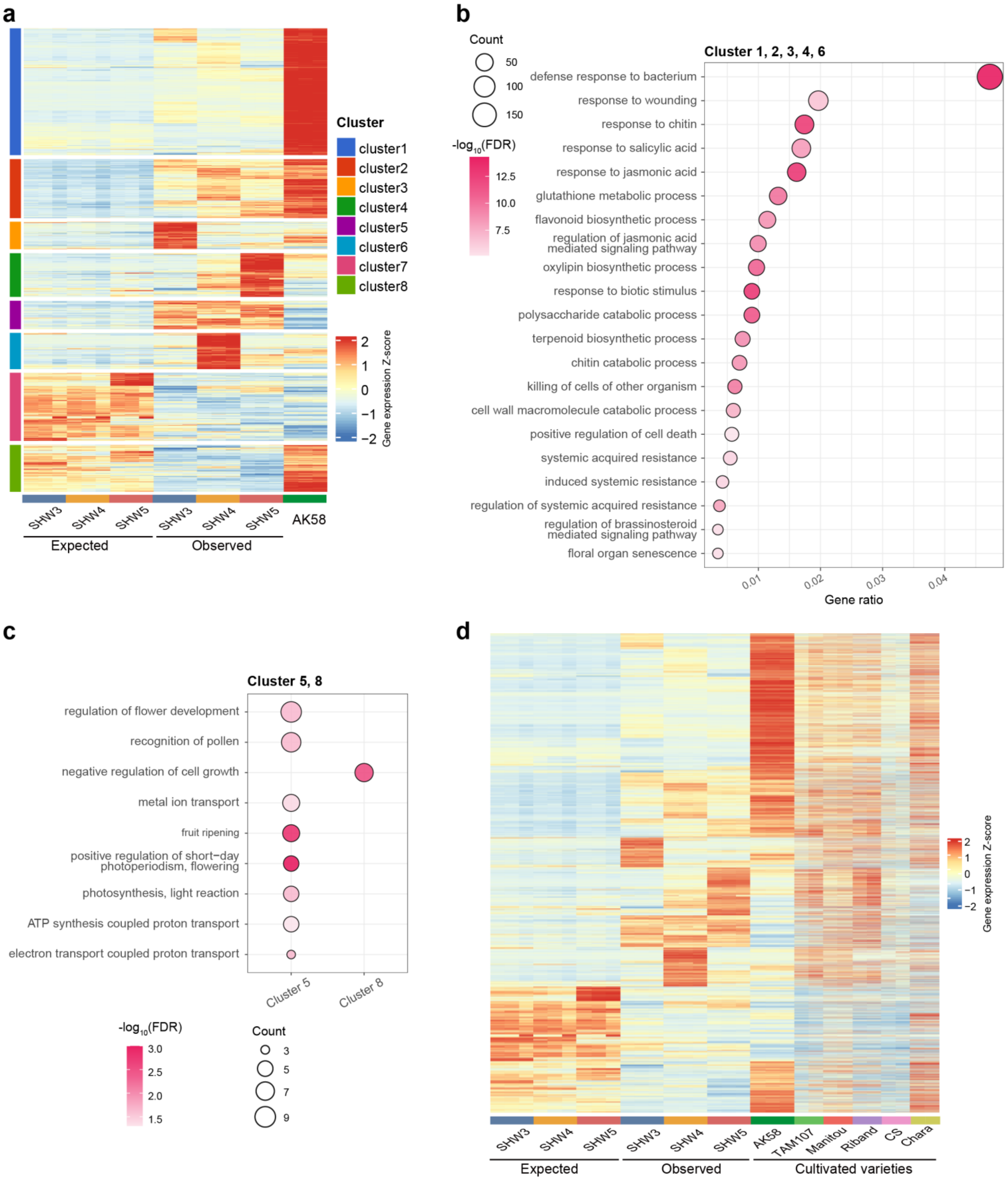

Figure S7. Expression analysis of DEGs identified in Figure 1g in cultivated hexaploid wheat. **a,** Heatmap showing the expression level of these DEGs in AK58 seedlings. **b**, Bubble diagram showing enriched biological process GO terms for genes in clusters 1, 2, 3, 4, 6 demonstrated in **a**. **c,** Bubble diagram showing enriched biological process GO terms for genes in cluster 5 and 8. The size of dots indicates number of genes in the terms, and the gradation of colors represents the significance as $-\log_{10}$ (FDR). **d**, Heatmap showing the expression level of these DEGs in other cultivated hexaploid varieties.

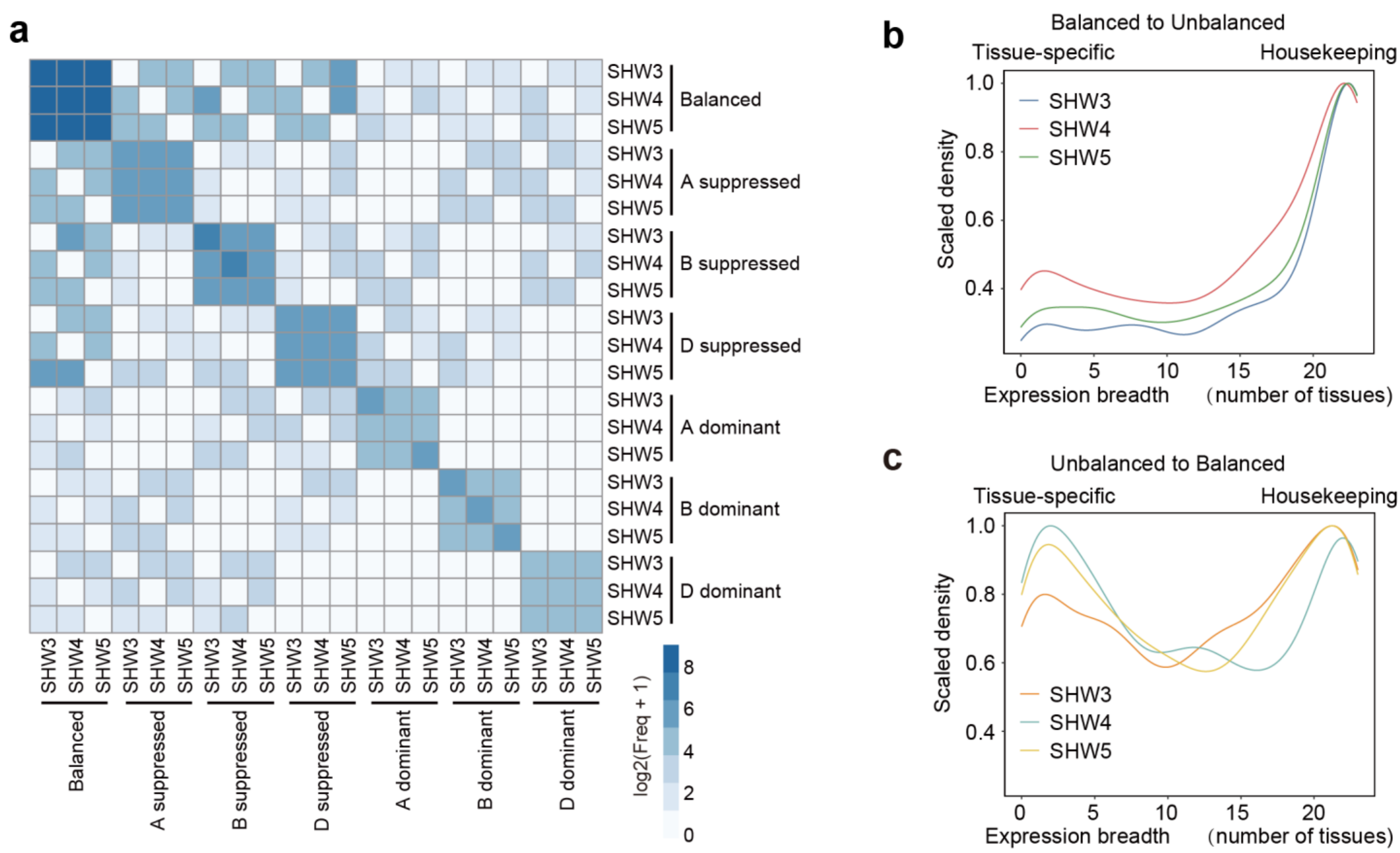

Figure S8. Dynamics of gene triads following hexaploidization events. a, Heatmaps showing relations of different triad categories of SHW3, SHW4 and SHW5. **b,c,** Expression breadth analysis of genes in triads showed balanced-to-unbalanced (**b**) or unbalanced-to-balanced (**c**) transition during hexaploidization. The more tissues in which a gene is expressed, the higher chance the gene is a housekeeping gene; while a gene is expressed in a few tissue types, it is a tissue-specific gene. Different color lines showing different SHWs.

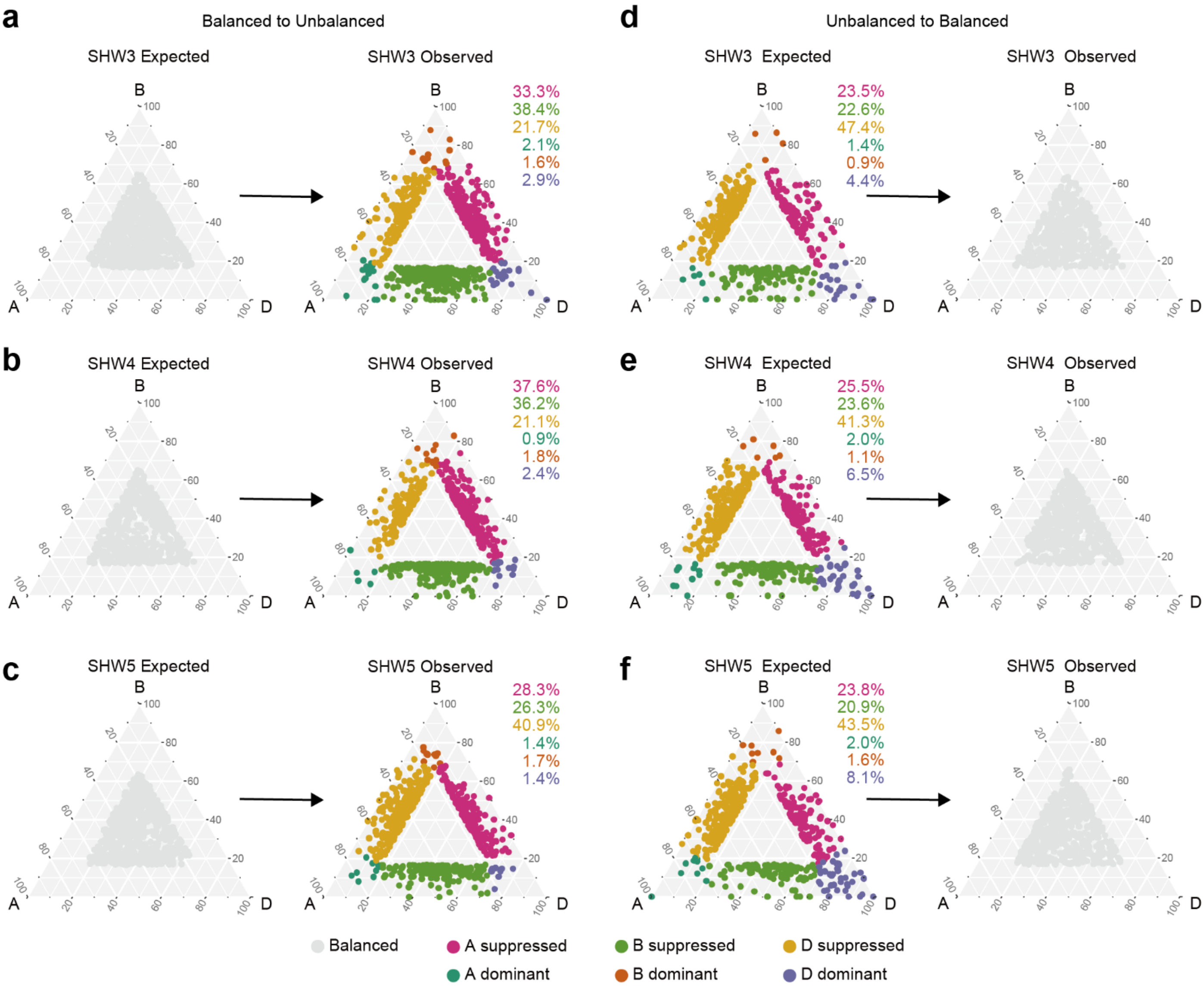

Figure S9. Ternary plots showing triads that altered their balancing state during polyploidizations. a-c, Points in ternary plots represent triads that showed expression bias state from balanced to unbalanced categories after polyploidizations in SHW3 (**a**), SHW4 (**b**) and SHW5 (**c**). **d-f,** Points in ternary plots indicate triads which showed expression bias state from unbalanced to balanced in SHW3 (**d**), SHW4 (**e**) and SHW5 (**f**). Points belong to different triad categories are shown with different colors. The proportion of unbalanced triad is given in the same color as the corresponding circles.

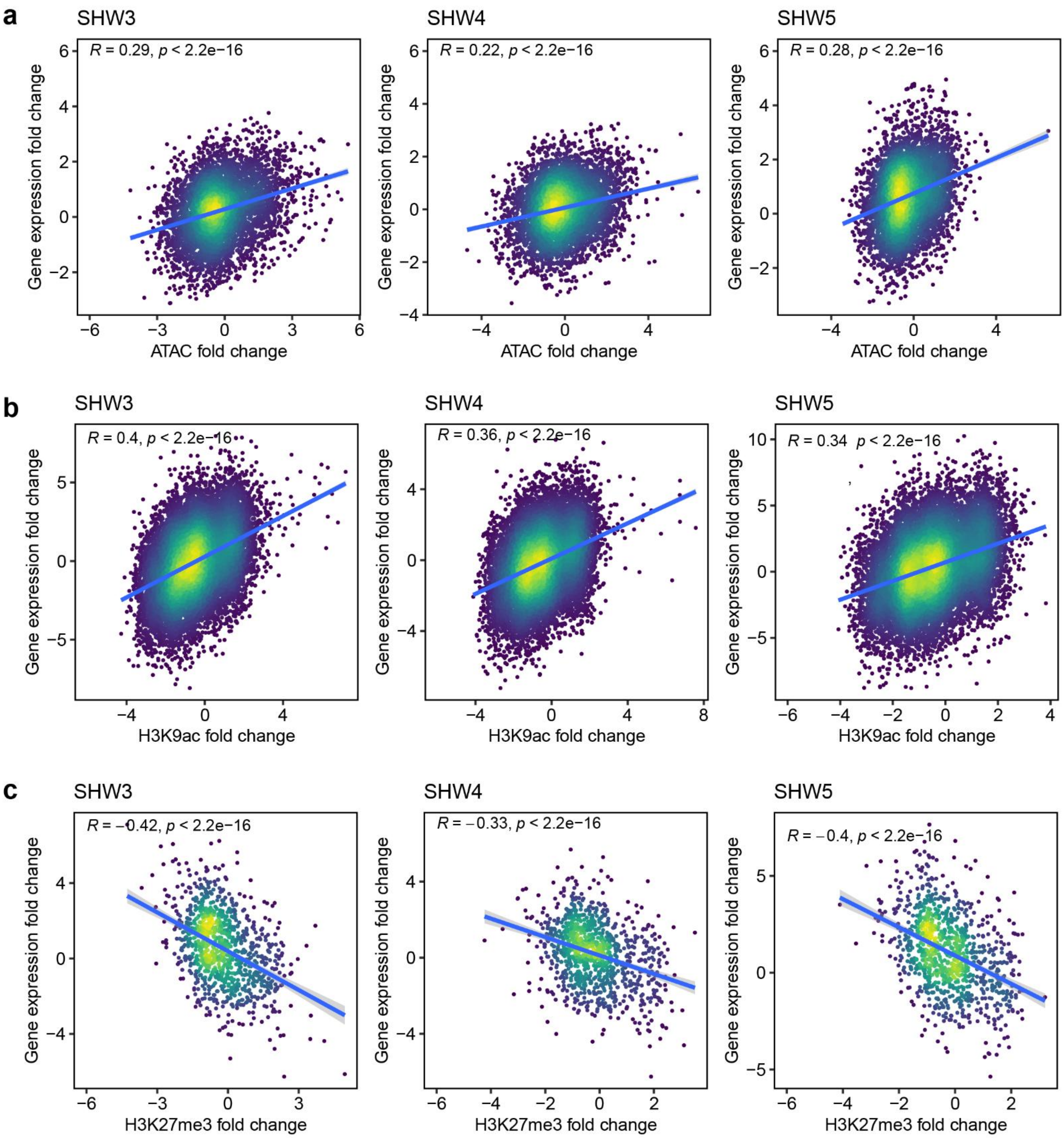

Figure S10. The relationship between gene expression fold change and peak fold change of ATAC-seq (a) and ChIP-seq of H3K9ac (b) and H3K27me3 (c) located in gene promoter and intragenic regions.

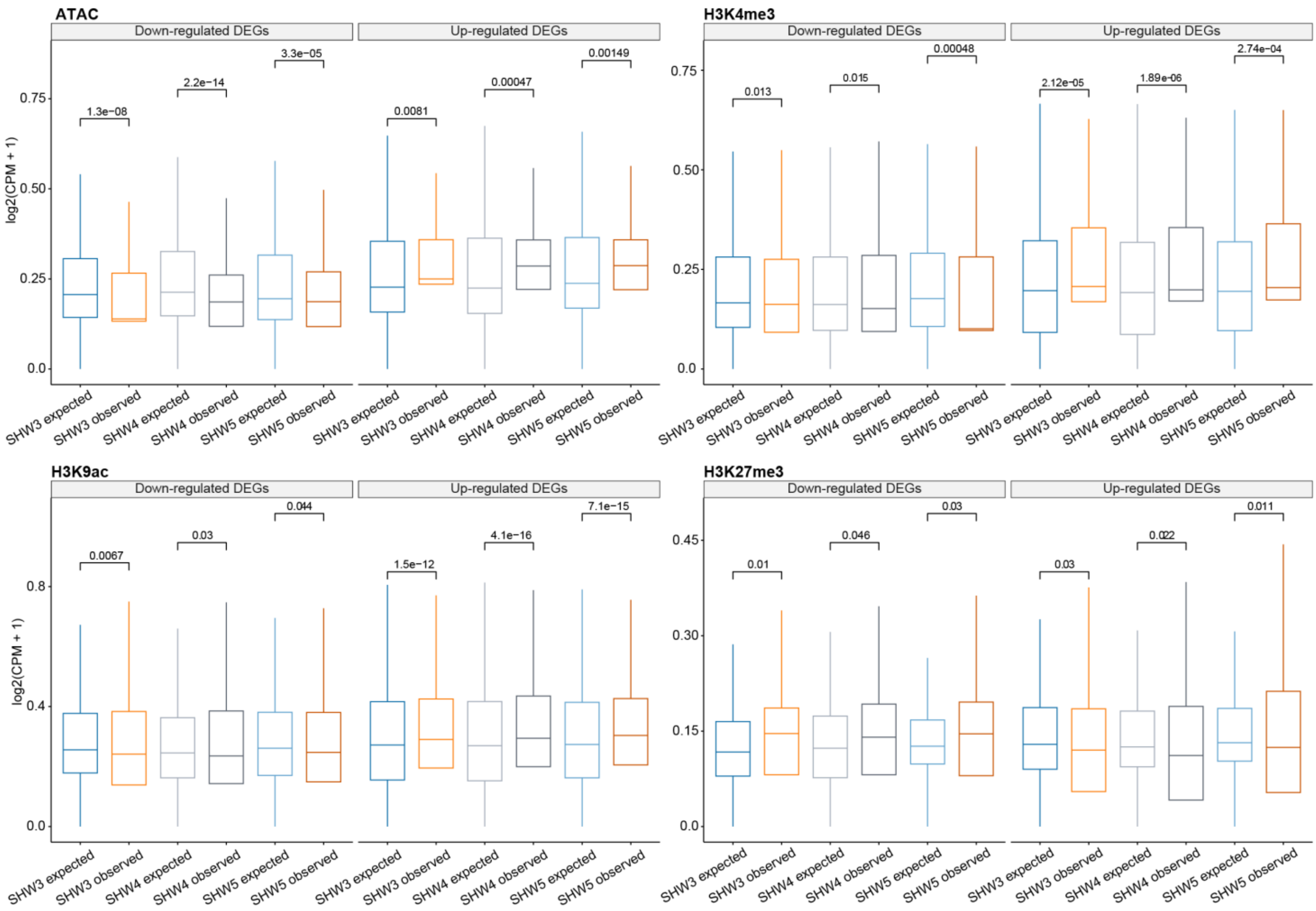

Figure S11. The enrichment of ATAC-seq peaks (representing chromatin accessibility) or H3K4me3, H3K9ac, and H3K27me3 modifications in the promoter and intragenic regions of up-regulated and down-regulated DEGs identified in this study. Comparisons were conducted between expected and observed data, and *P*-values (two-sided *t*-test) are shown above the boxes.

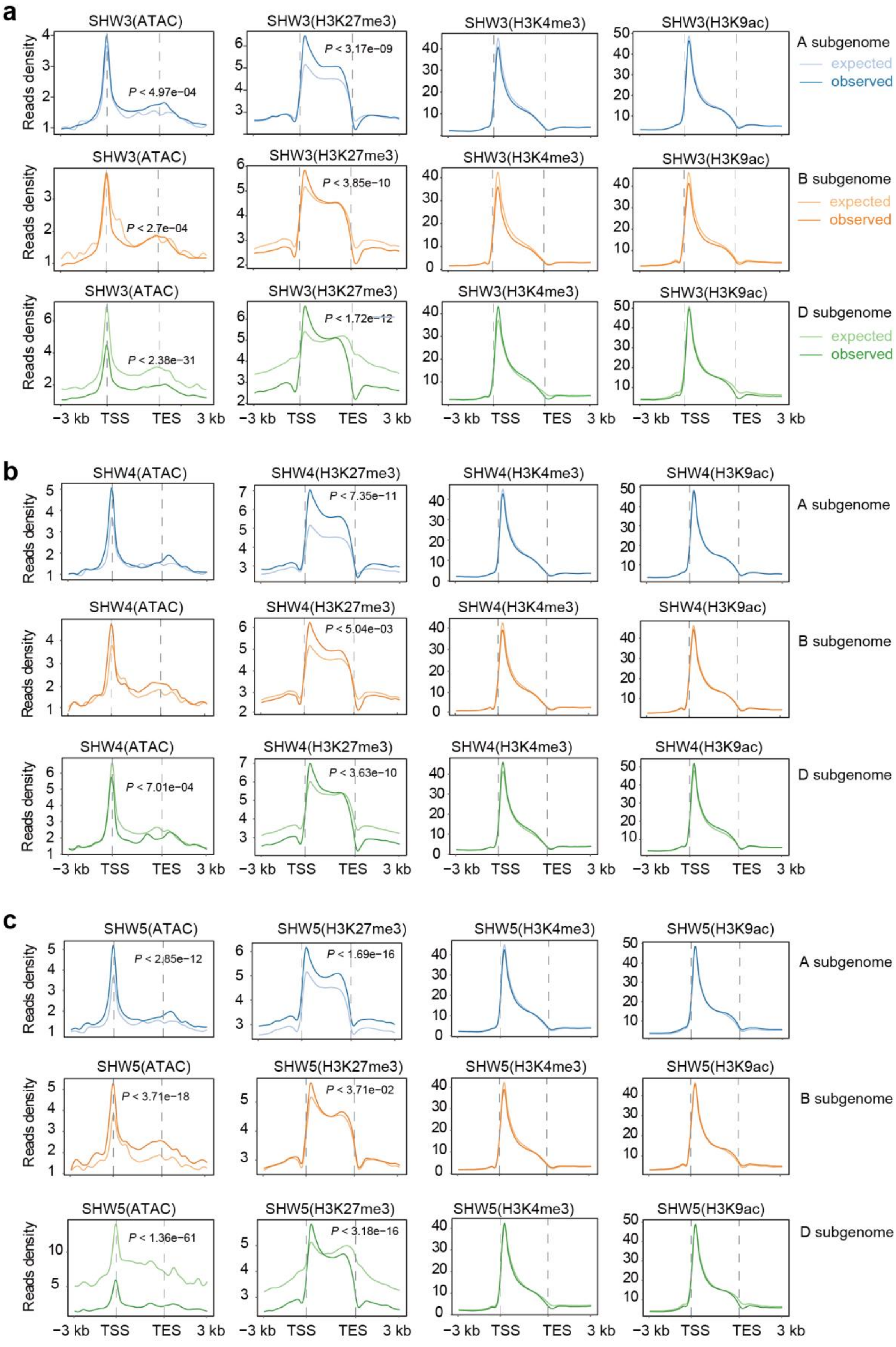

Figure S12. Comparing epigenomic changes in SHWs before and after hexaploidization. a-c, Chromatin accessibility (ATAC-seq) and histone modification levels of H3K27me3, H3K4me3, H3K9ac in A (top), B (middle) and D (bottom) subgenomes of SHW3 (**a**), SHW4 (**b**) and SHW5 (**c**). Light lines represent reads density before hexaploidization (expected); dark lines indicate reads density after hexaploidization (observed).

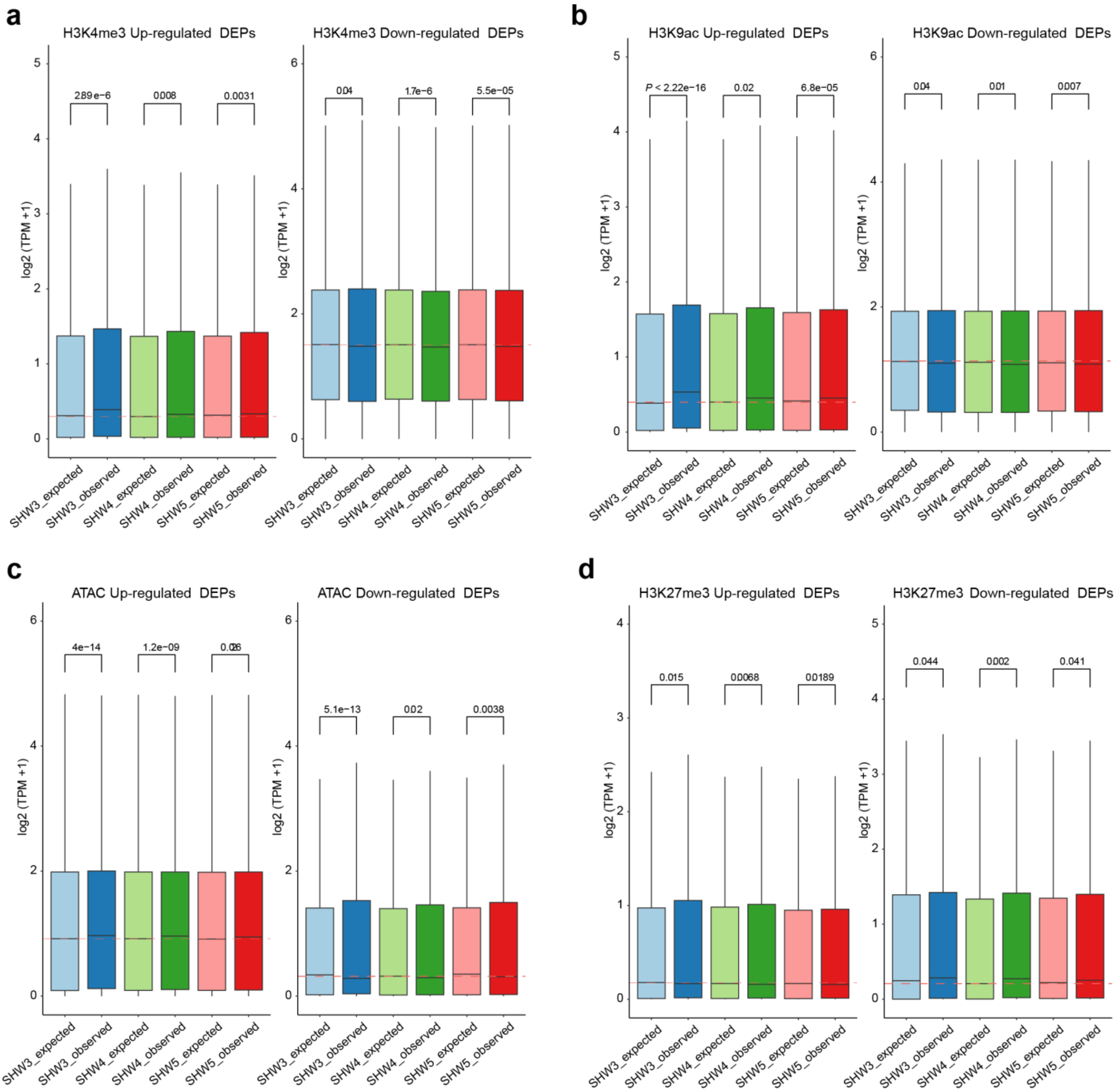

Figure S13. Expression level analysis of genes with up-regulated or down-regulated DEPs of H3K4me3 (a), H3K9ac (b), ATAC-seq (c), and H3K27me3 (d). Comparisons were conducted between expected and observed data, and *P*-values (two-sided *t*-test) are shown above the boxes.

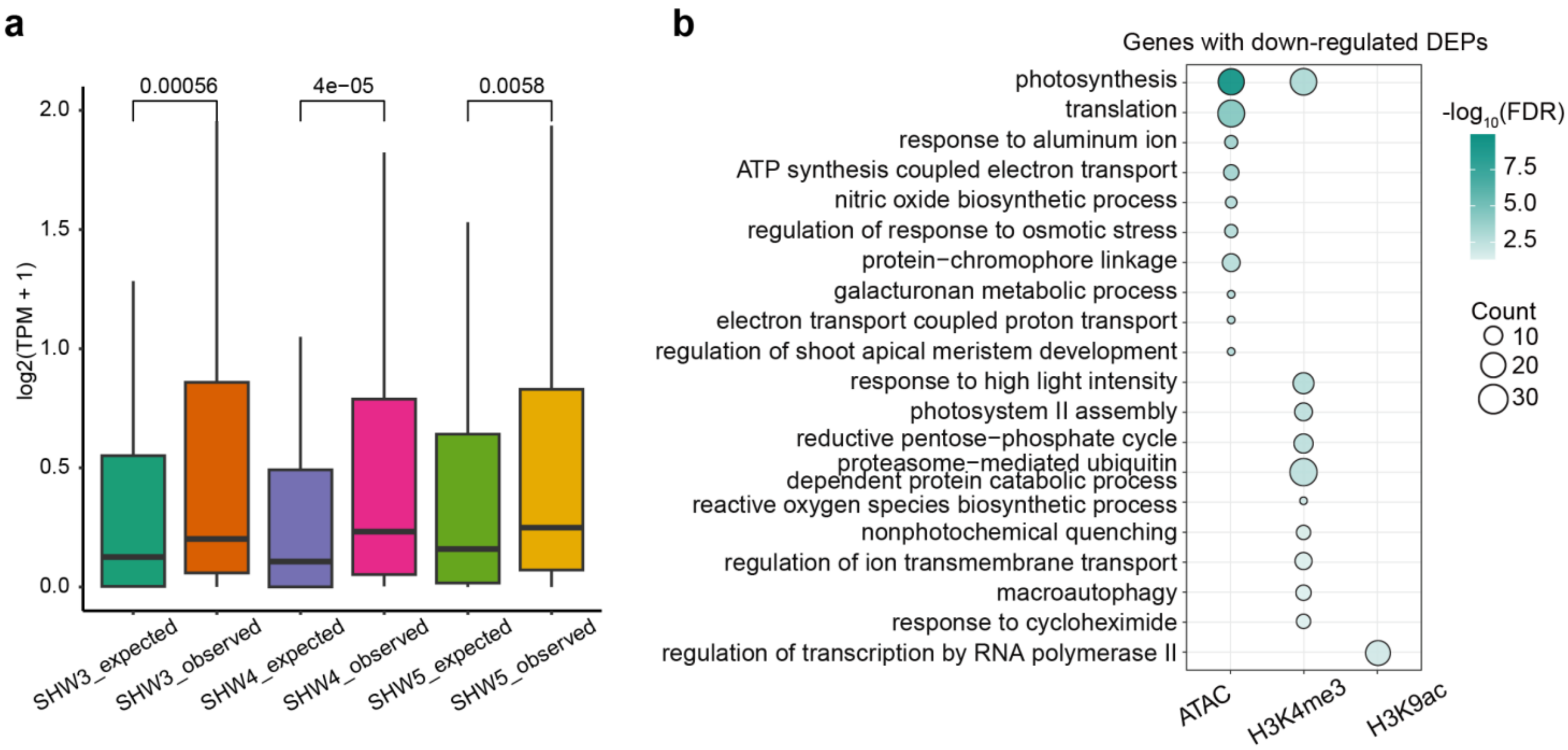

Figure S14. Analysis of genes with DEPs during wheat hexaploidization. a, Expression level of environmentally responsive genes identified in GO enrichment analysis of up-regulated DEPs. Comparisons were conducted between expected and observed data, and *P*-values (two-sided *t*-test) are shown above the boxes. **b,** GO enrichment analysis of genes with down-regulated DEPs. Color gradation indicates the significance as $-\log_{10}$(FDR). Dot size represents gene number enriched in that term.

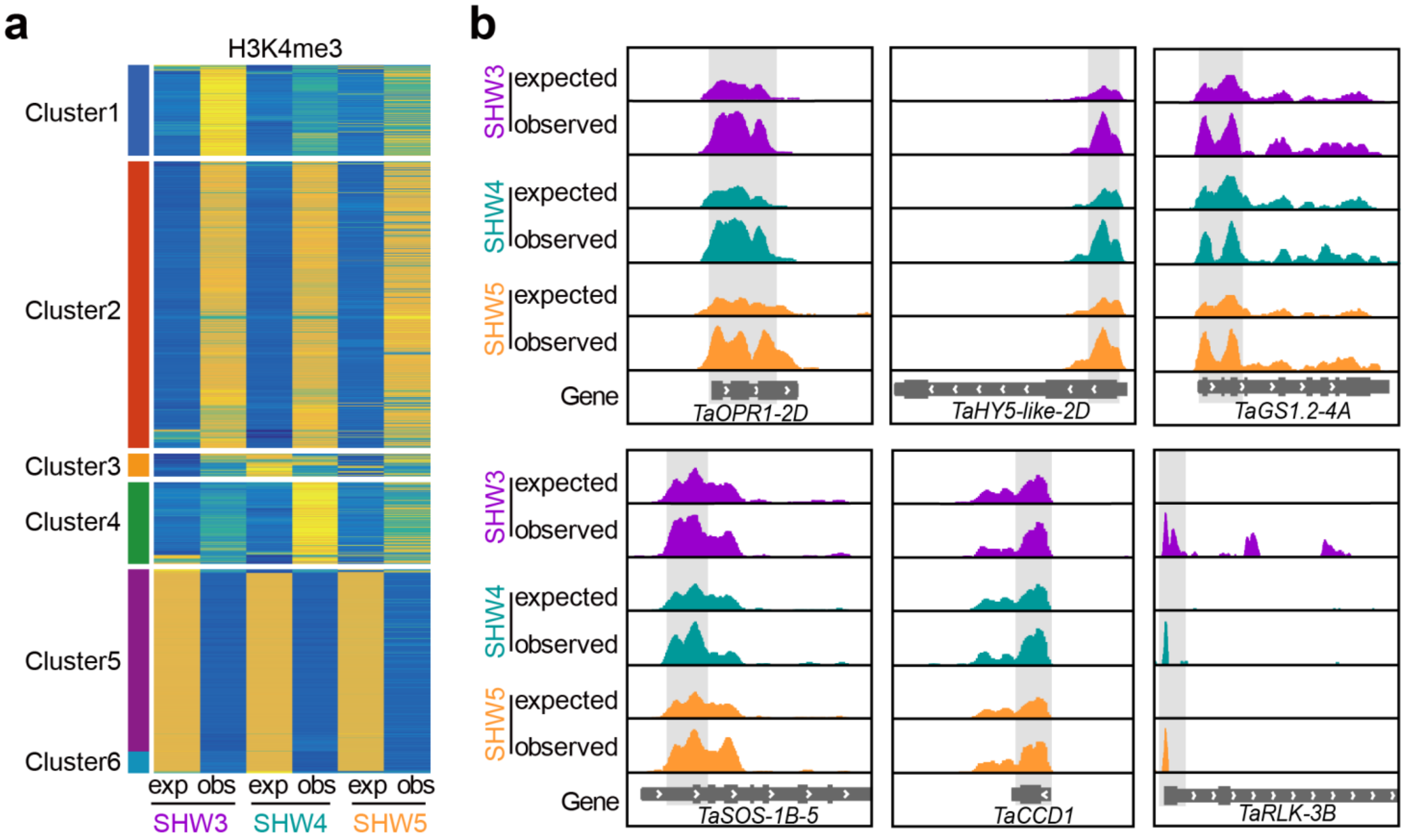

Figure S15. Analysis of H3K4me3 differential peaks during different hexaploidization events of SHW3, SHW4, and SHW5. a, Heatmap showing clusters of DEPs during wheat hexaploidization. **b**, H3K4me3 enrichment of different adaptive genes in SHW3, SHW4 and SHW5 before (expected) or after (observed) D-genome integration.

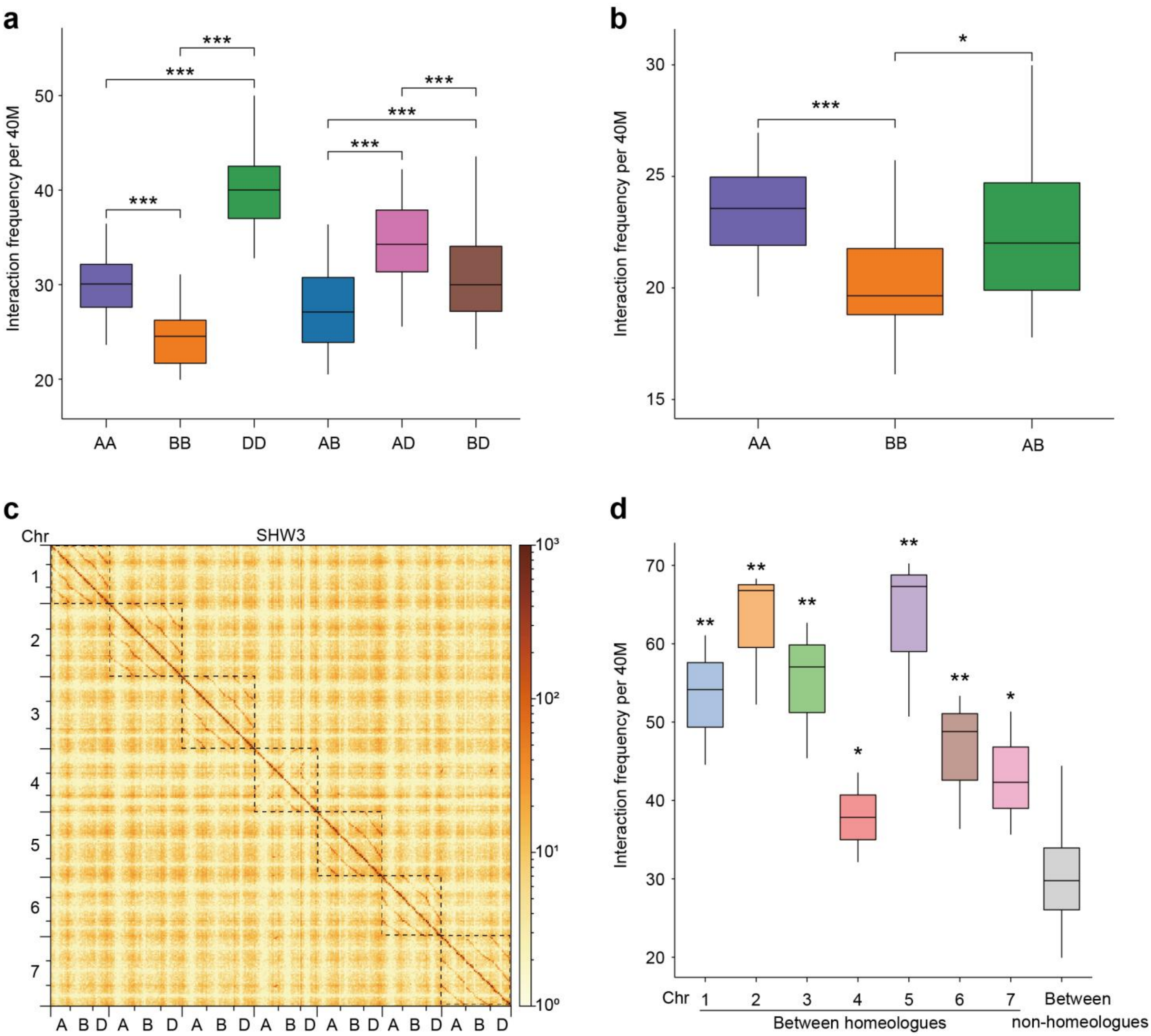

Figure S16. Analysis of H3K4me3-associated 3D chromatin interactions in SHW3 and its tetraploid parent. a,b, Boxplots representing interaction frequency within or between subgenomes in SHW3 (**a**) and its tetraploid parent (**b**). **c,** Heatmap showing chromatin interactions between homeologues associated with H3K4me3 modification in SHW3. **d,** Boxplots showing interaction frequency between homoeologous chromosomes compared with non-homoeologous chromosomes of SHW3. *$P < 0.05$, **$P < 0.01$, ***$P < 0.001$, two-sided *t*-test.

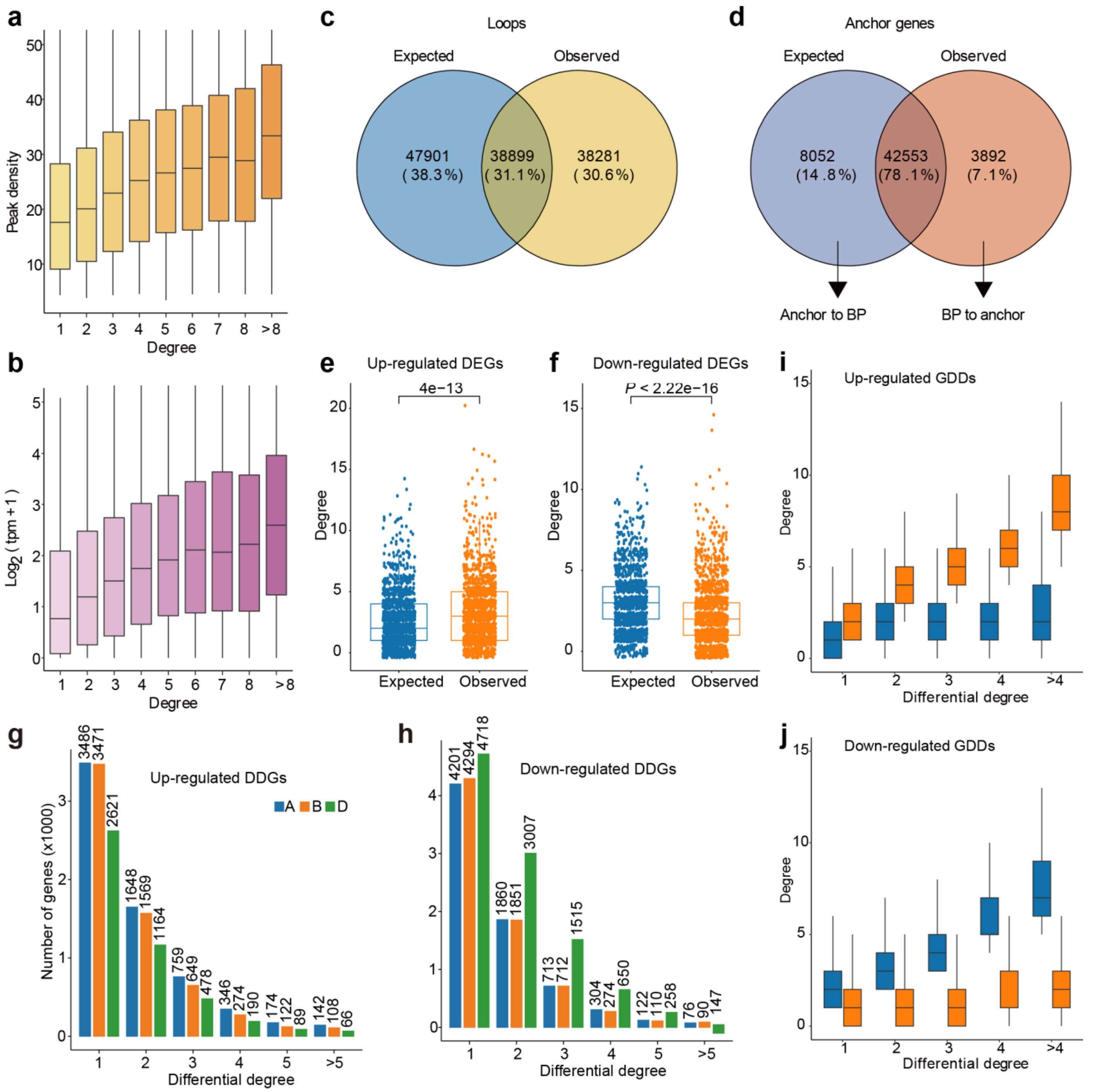

Figure S17. Characterization of H3K4me3-associated chromatin interactions and genes showed altered interaction degree during wheat hexaploidization. a,b, Boxplots showing the positive correlation of H3K4me3 peak intensity (**a**) and expression level (**b**) with interaction degree. Expression levels showing as $\log_2$ (tpm + 1).**c,d,** Venn diagram showing overlaps of interaction loops (**c**) and anchor genes (**d**) in expected and observed dataset. **e,f**, Analysis of chromatin interaction degrees of up-regulated DEGs (**e**) and down-regulated DEGs (**f**) before and after D-genome integration. Comparisons were conducted between expected and observed data, and *P*-values (two-sided *t*-test) are shown above the boxes. **g,h,** Statistics of up-regulated genes with different degree (GDDs) **(g)** and down-regulated GDDs **(h)** in different subgenomes during wheat hexaploidization. **i,j**, Interaction degree of up-regulated GDDs (**i**) and down-regulated GDDs (**j**) with differential degree.

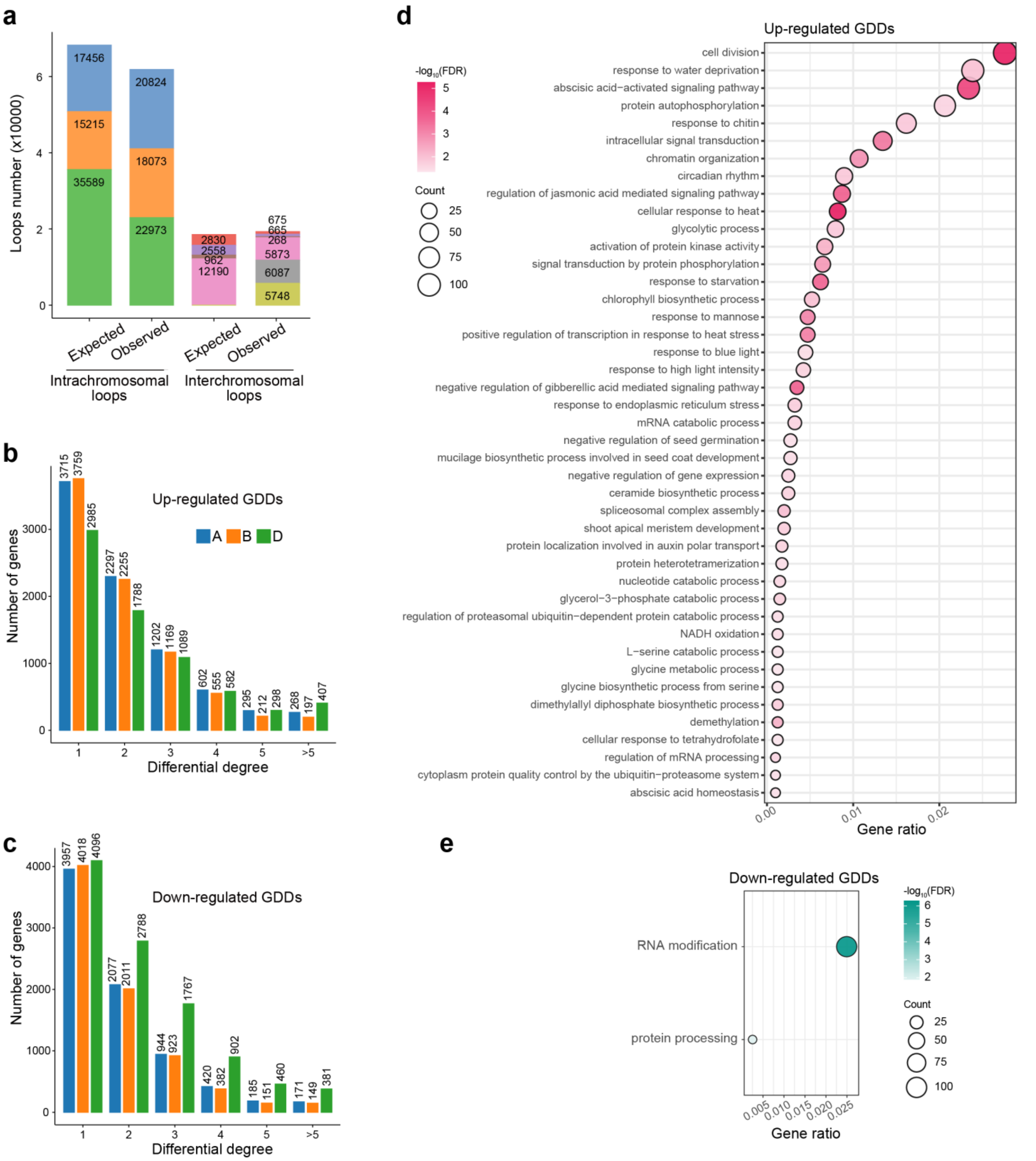

Figure S18. Characterization of H3K4me3-associated chromatin interactions in cultivated AK58 in comparison with expected dataset. a, Comparison of the intrachromosomal and interchromosomal loops between AK58 and expected dataset. **b,c,** Statistics of up-regulated GDDs (**b**) and down-regulated GDDs (**c**) in different subgenomes identified in comparison between AK58 and expected dataset. **d,e**, Bubble diagram showing enriched GO terms of up-regulated GDDs (**d**) and down-regulated GDDs (**e**) in comparison between AK58 and expected dataset. The dot size represents the number of genes in the corresponding GO term, and the gradation of color indicates the significance of terms as $-\log_{10}$ (FDR).

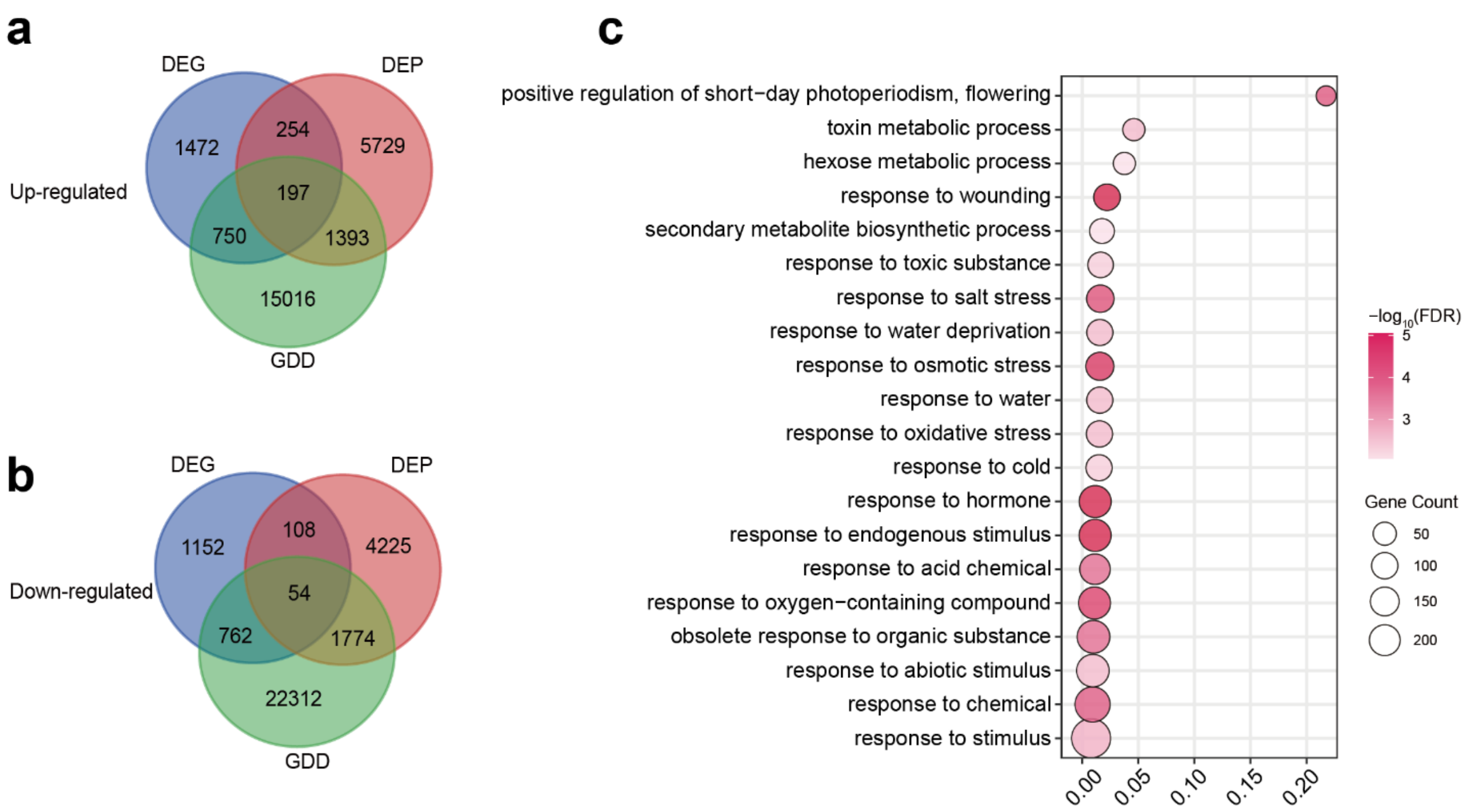

Figure S19. Overlapped genes that showed difference in transcriptome, epigenome, and 3D interactome before and after D-genome integration. a, Venn diagram showing the overlap of up-regulated DEGs, DEP (increased H3K4me3/H3K9ac or decreased H3K27me3 modification) related genes, and up-regulated GDDs in SHW3. DEP-related genes and GDDs were further merged and tested for enrichment in DEGs, revealing a significant overrepresentation of up-regulated DEGs in the combined DEP-related/GDD gene set (fold enrichment = 2.89, OR = 4.61, *P*-value = 2.94e-298, Fisher's exact test). **b**, Venn diagram showing the overlap of down-regulated DEGs, DEP (decreased H3K4me3/H3K9ac or increased H3K27me3 modification) related genes, and down-regulated GDDs in SHW3. DEP-related genes and GDDs were further merged and tested for enrichment in DEGs, revealing a significant overrepresentation of down-regulated DEGs in the combined DEP-related/GDD gene set (fold enrichment = 2.28, OR = 3.39, *P*-value =4.82e-150, Fisher's exact test). **c**, Bubble diagram showing top twenty GO terms enriched in up-regulated DEGs that showing epigenetic remodeling and increased chromatin interaction during hexaploidization. Dot size represents the gene number enriched in GO terms and color gradation represents the significance of enrichment as $-\log_{10}$ (FDR).

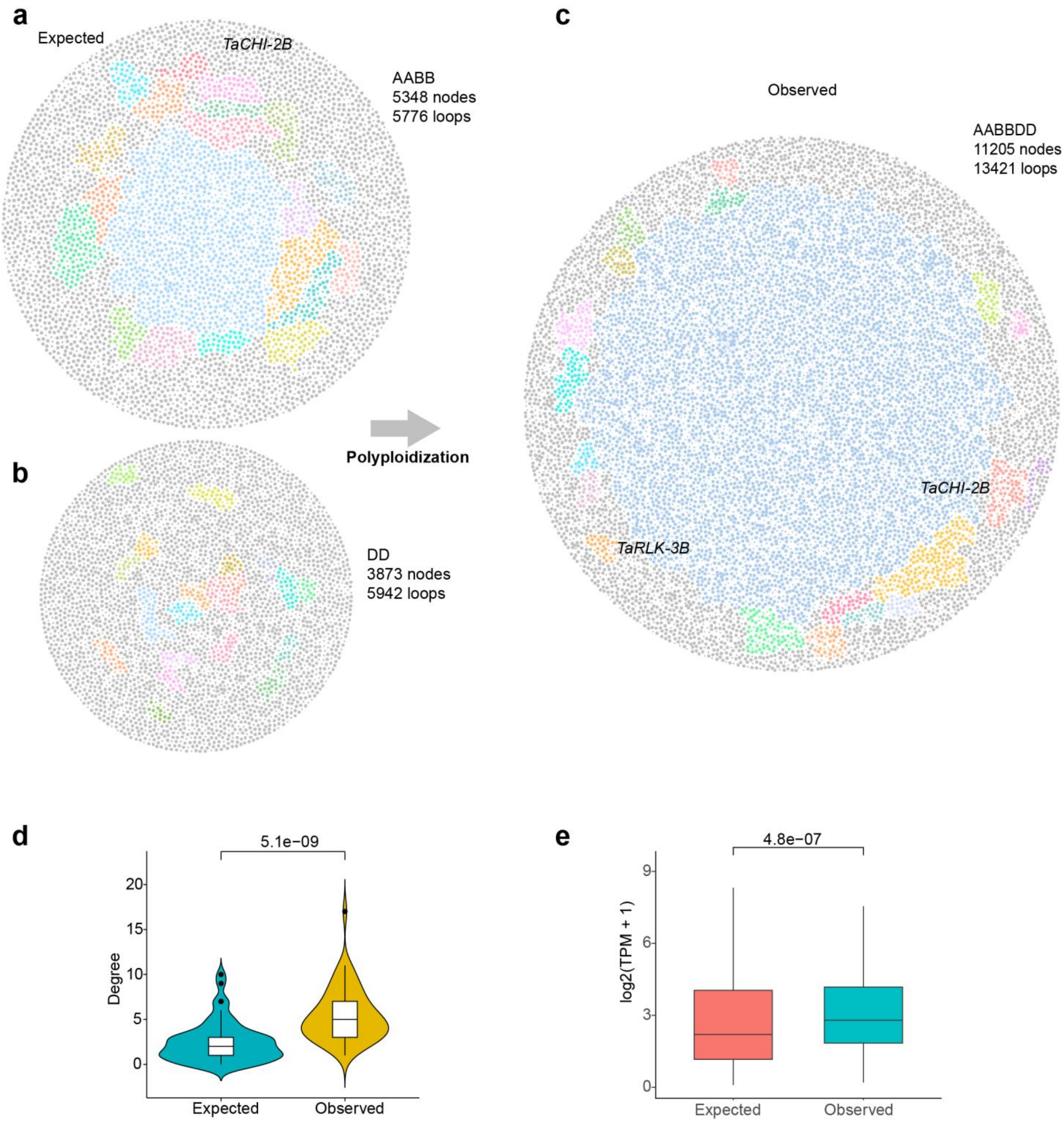

Figure S20. Dynamics of H3K4me3-associated chromatin architecture following wheat hexaploidization event. a-c, Chromatin interaction networks of the core 1545 DEGs in SHW3 (**c**) and its tetraploid (**a**) and diploid (**b**) parents. The top 20 modules are presented using different color. **d,e**, Comparison between expected and observed dataset of chromatin interaction degree (**d**) and expression level (**e**) of adaptative genes identified in Figure 3a-c. *P*-values (two-sided *t*-test) are shown above the boxes.

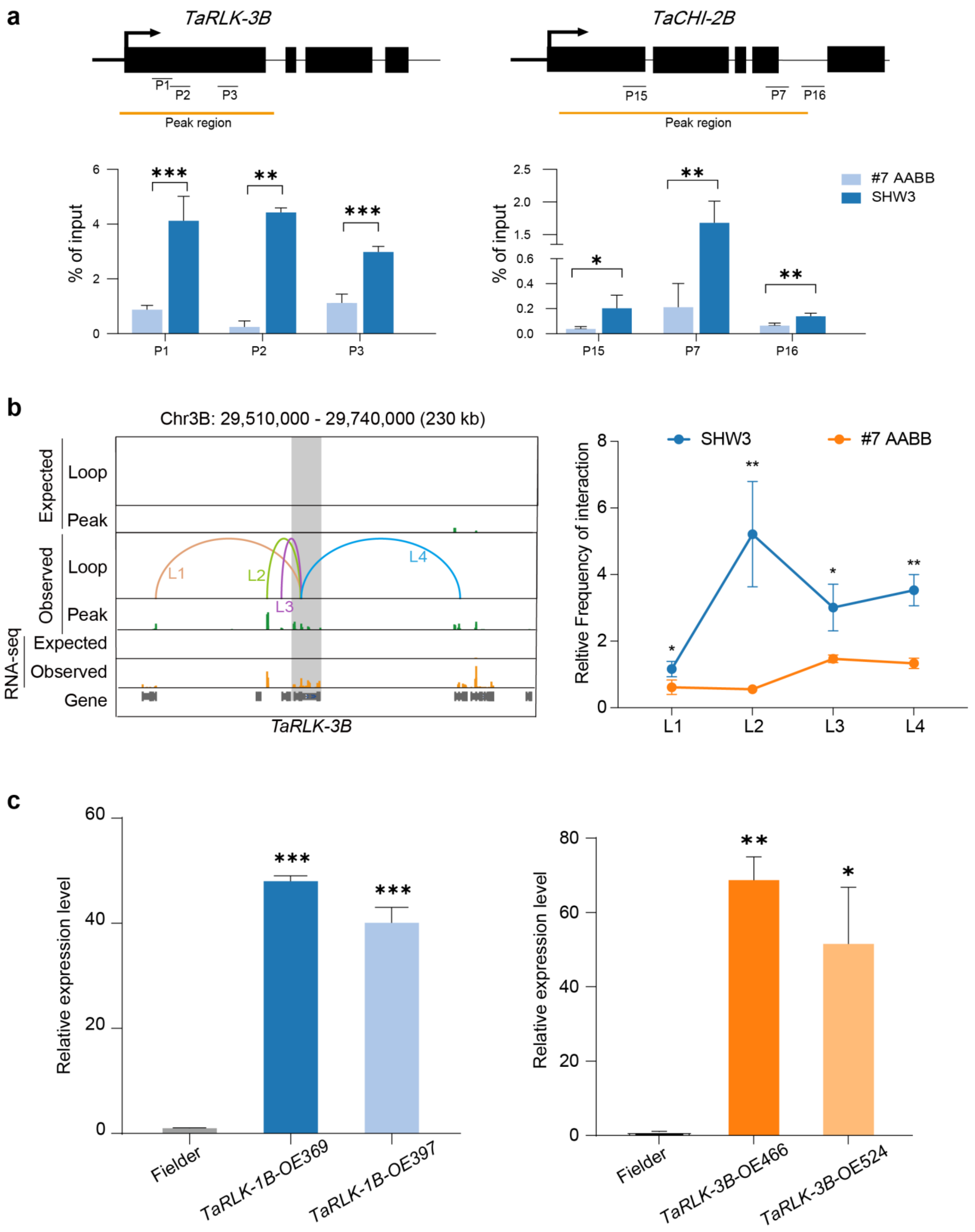

Figure S21. Verification of alteration identified in candidate genes during hexaploidization. a, ChIP-qPCR verified the H3K4me3 enrichment in *TaRLK-3B* and *TaCHI-2B* genomic regions. **b,** 3C-qPCR verified the newly-built chromatin loops connected *TaRLK-3B* genomic region. **c**, The expression level of *TaRLK-1B* (left) and *TaRLK-3B* (right) in their overexpression transgenic lines. $^{*}P < 0.05$, $^{**}P < 0.01$, $^{***}P < 0.001$, two-sided *t*-test.

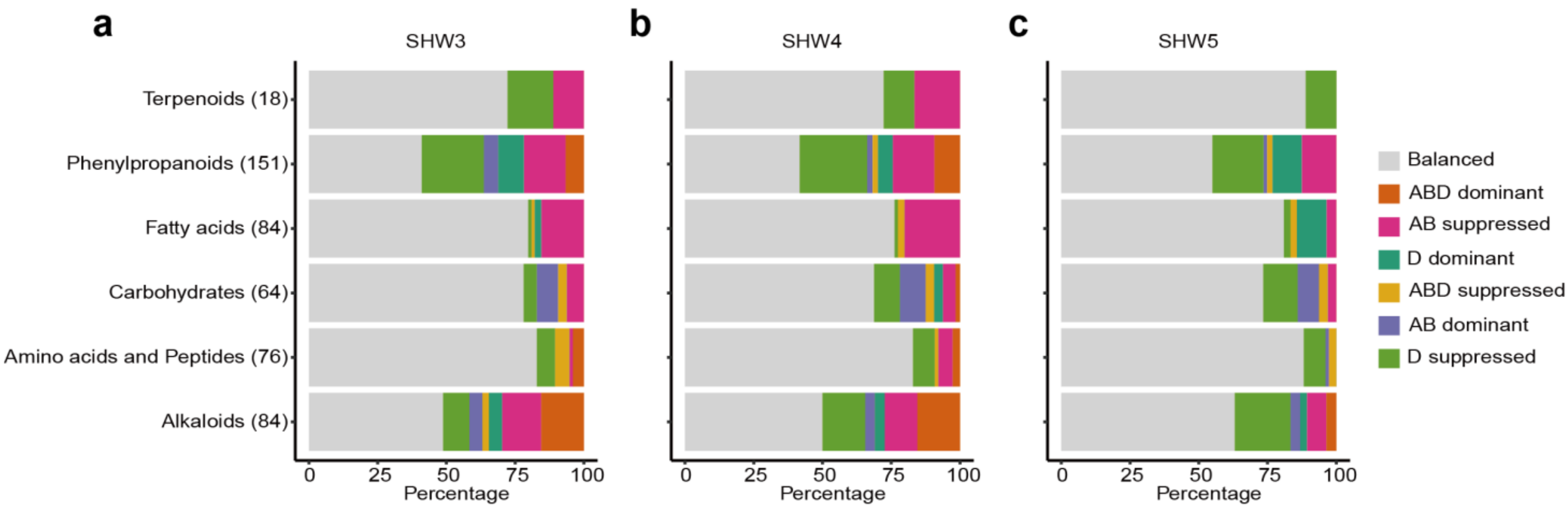

Figure S22. Comparison of metabolome in SHWs and its parents. a-c, The percentage of seven categories of content balance between SHWs and their tetraploid, diploid parents of SHW3 (**a**), SHW4 (**b**), SHW5 (**c**) in metabolite classes with annotated structure.

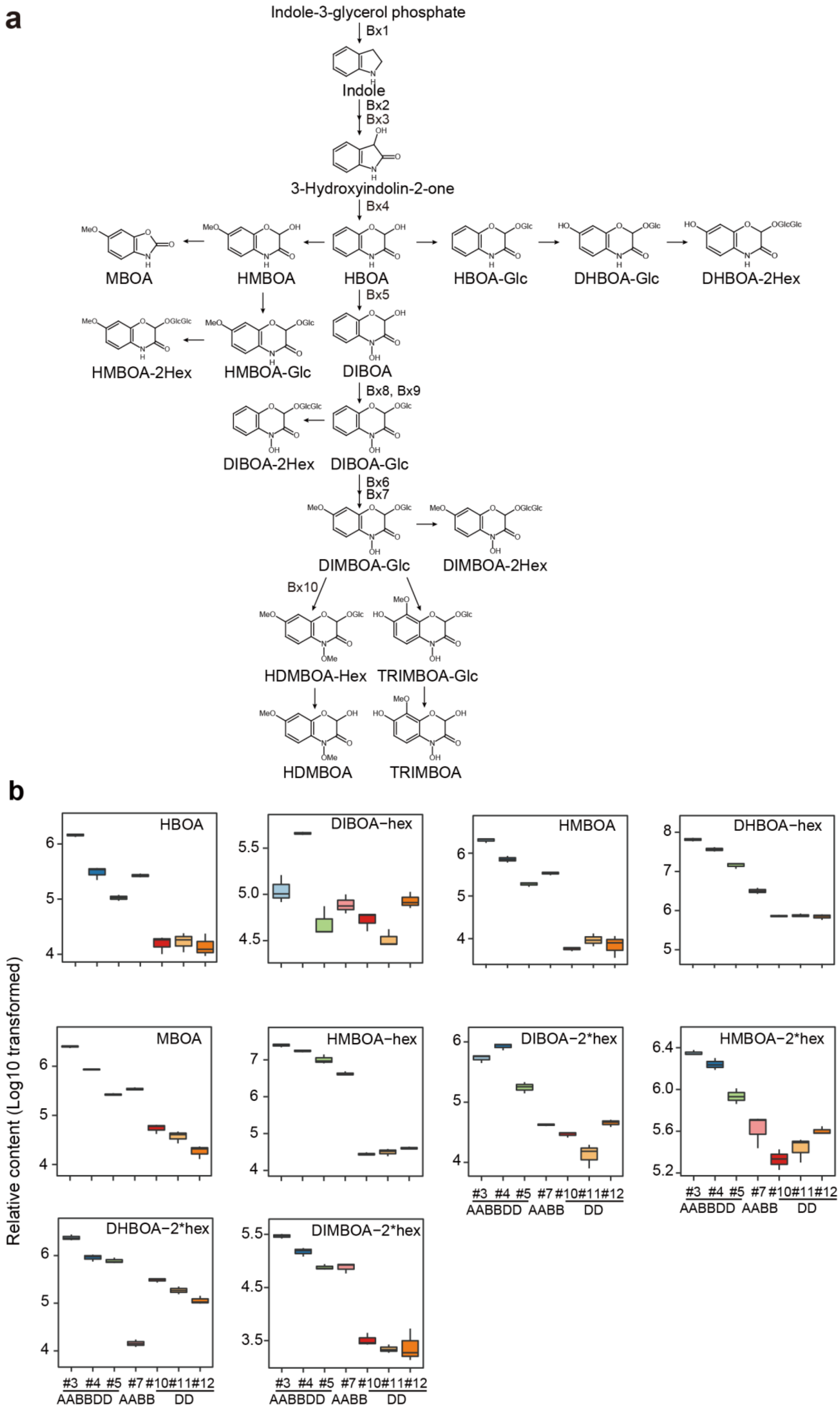

Figure S23. Metabolites in benzoxazinoid pathway detected in this study. a, The putative biosynthesis pathway of benzoxazinoids (BXs) and BX derivatives detected in this study. **b,** Relative contents of benzoxazinoid derivatives in hexaploid SHWs and its tetraploid and dipoid parents. Contents are given as log_{10} transformed.

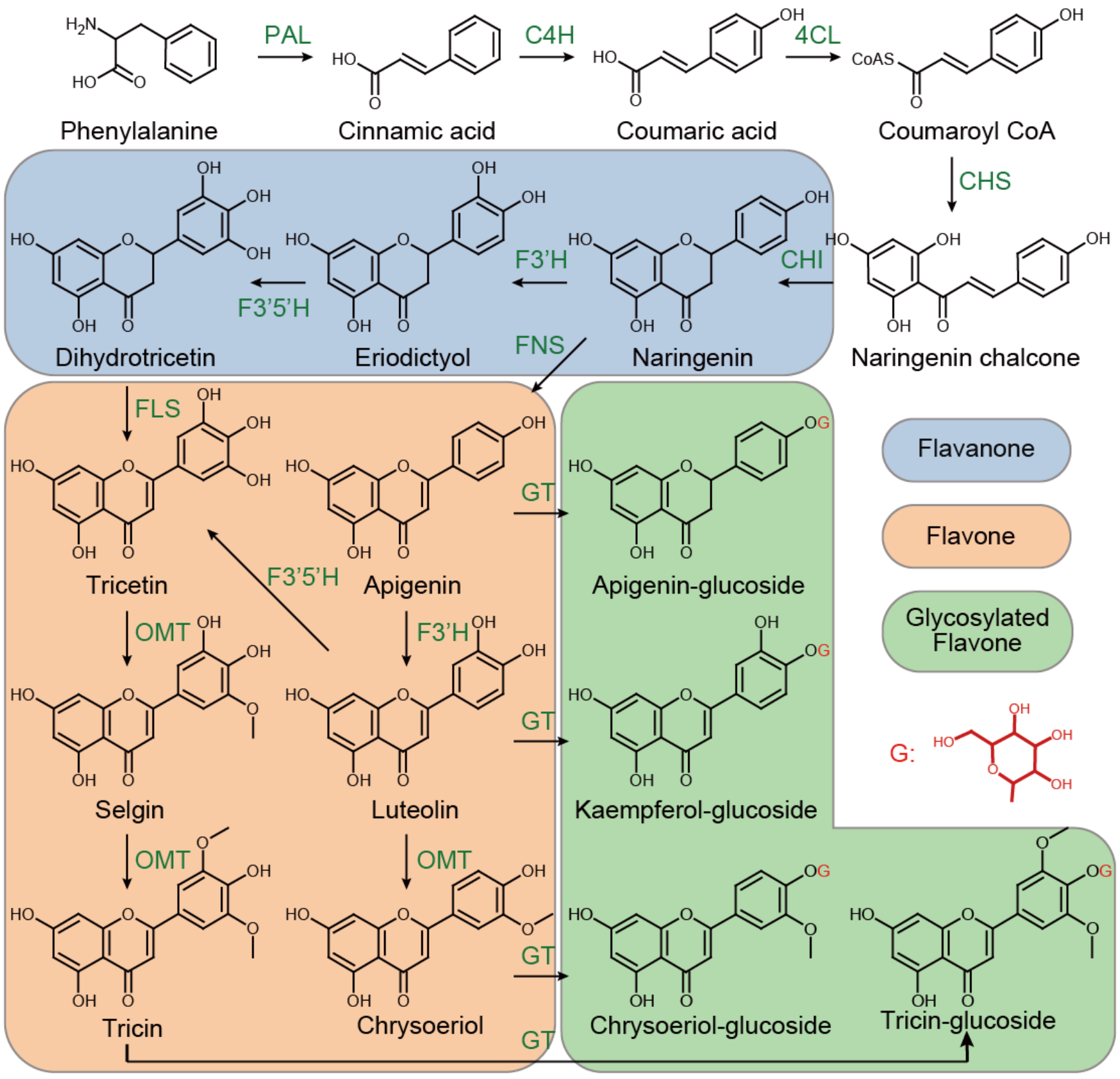

Figure S24. The core flavonoid pathway in plants. The different colored shadow indicates different classes of flavonoids.

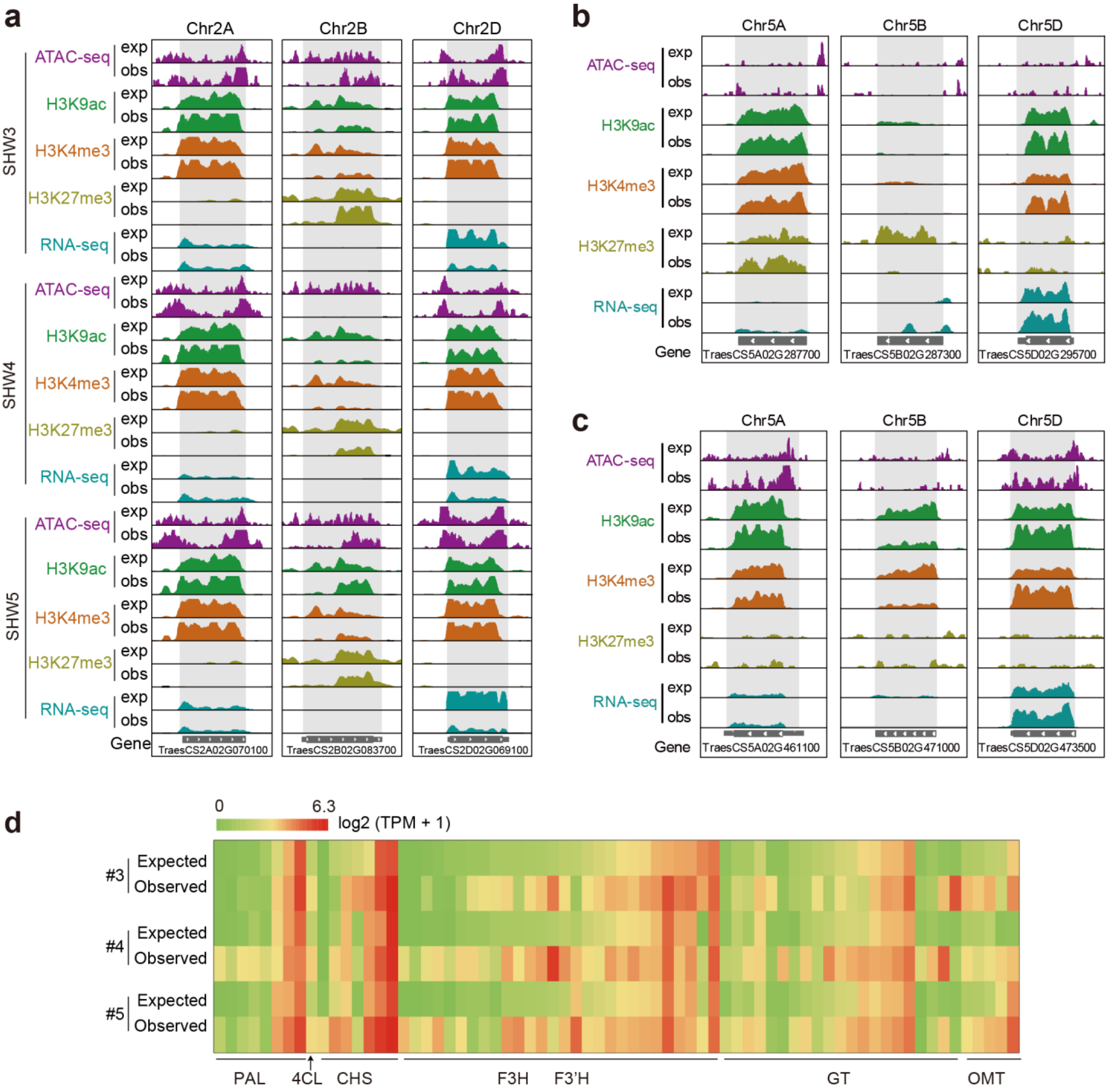

Figure S25. Epigenomic features of flavonoid pathway genes during D-genome integration-mediated hexaploidization. a, Epigenomic features of *TaGT-2D* (*TraesCS2D02G069100)* homoeologs in SHW3, SHW4 and SHW5 before (exp) or after (obs) D-genome integration. **b,c** Epigenomic features of subgenome homoeolog genes of *TraesCS5D02G295700* (**b**) and *TraesCS5D02G473500* (**c**) in SHW3 during polyploidization. The highlighted regions in grey representing the gene body. **d**, Heatmap showing putative flavonoid pathway genes with activated expression after D-genome integration.

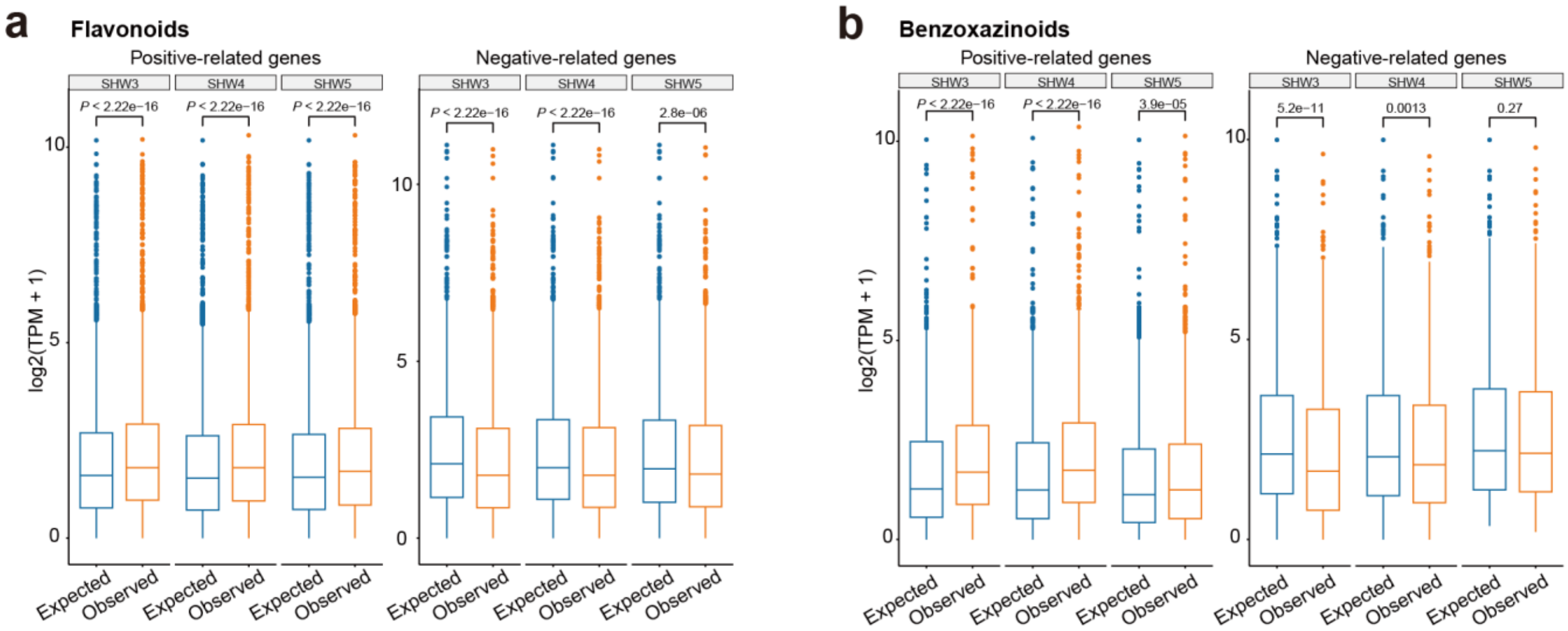

Figure S26. Expression level analysis of flavonoids (a) and benzoxazinoids (b) correlated genes during hexaploidization progress. Comparisons were conducted between expected and observed dataset, *P*-values (two-sided *t*-test) are shown above the boxes.

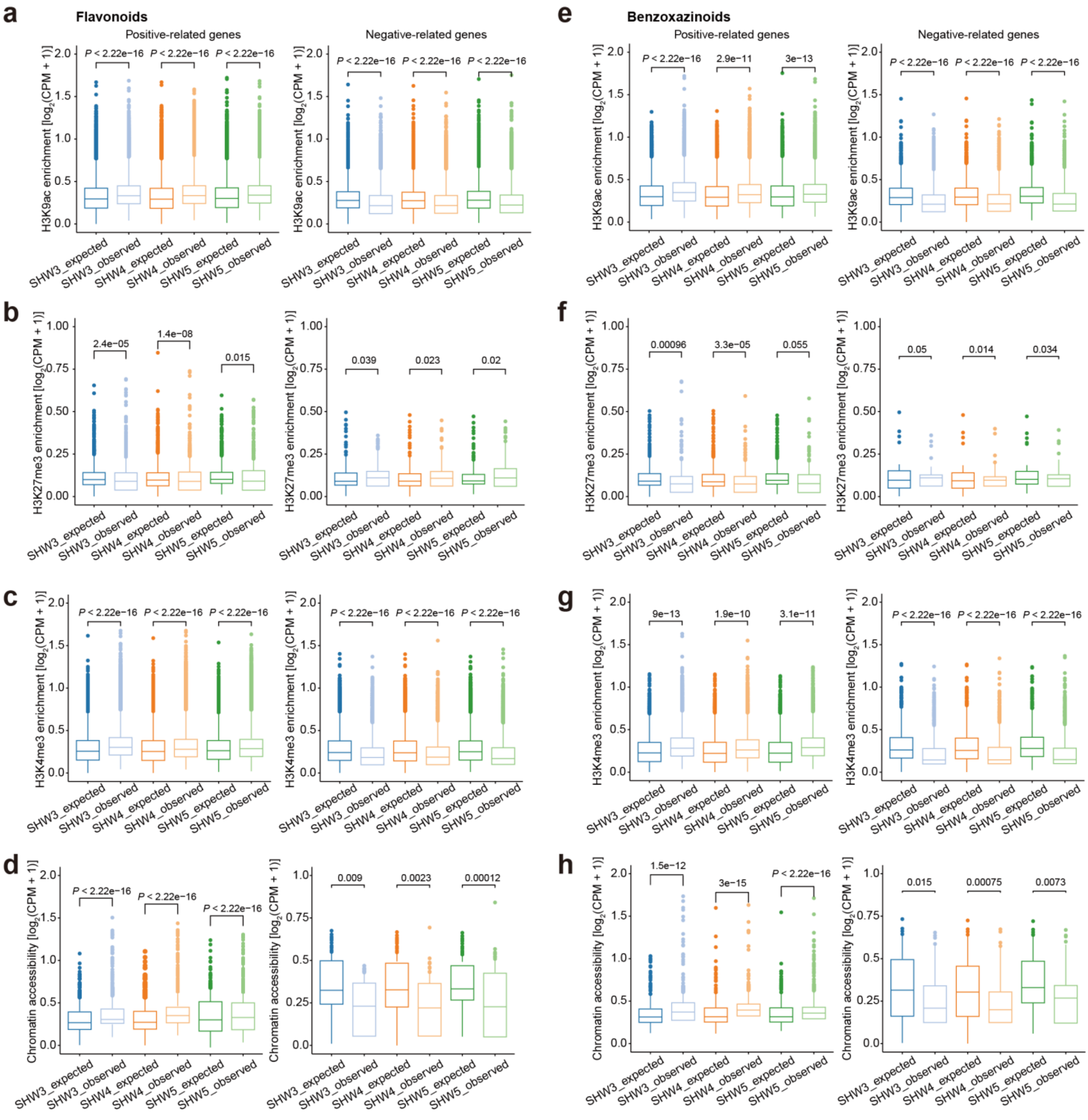

Figure S27. Dynamics of epigenomic status of flavonoids and benzoxazinoids correlated genes in expected and observed datasets. a-d, Analysis of H3K9ac (**a**), H3K27me3 (**b**), H3K4me3 (**c**) enrichment, and chromatin accessibility (**d**) of flavonoids positive-related and negative-related genes. **e-h**, Analysis of H3K9ac (**e**), H3K27me3 (**f**), H3K4me3 (**g**) enrichment, and chromatin accessibility (**h**) of benzoxazinoids positive-related and negative-related genes. Comparisons were conducted between expected and observed data, *P*-values (two-sided *t*-test) are shown above the boxes.

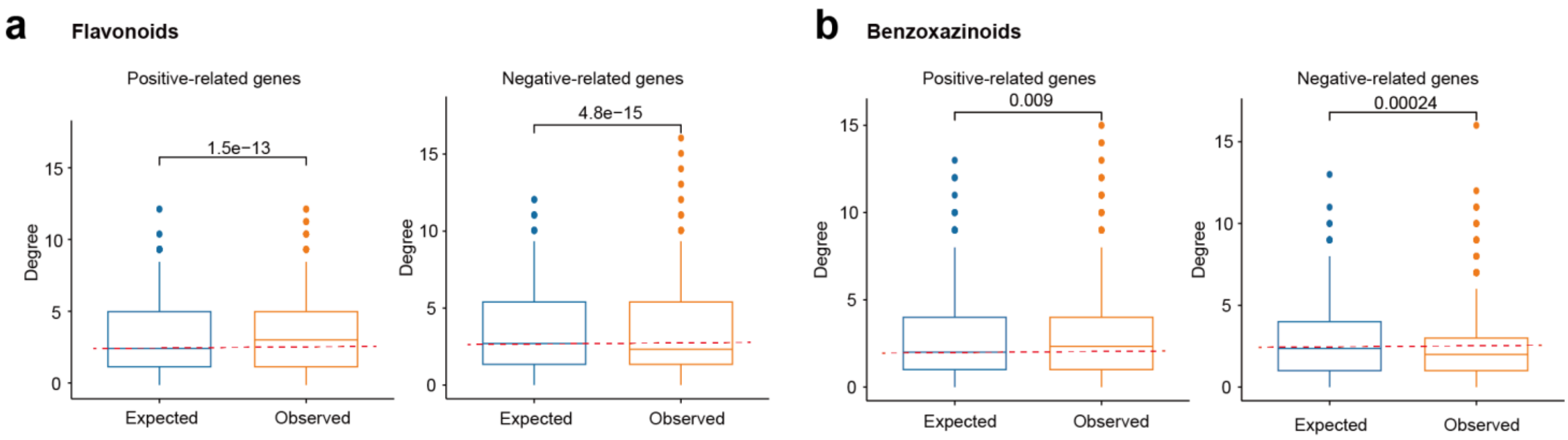

Figure S28. Analysis of chromatin interaction degree of flavonoids (a) and benzoxazinoids (b) correlated genes during hexaploidization progress. Comparisons were conducted between expected and observed data, *P*-values (two-sided *t*-test) are shown above the boxes.

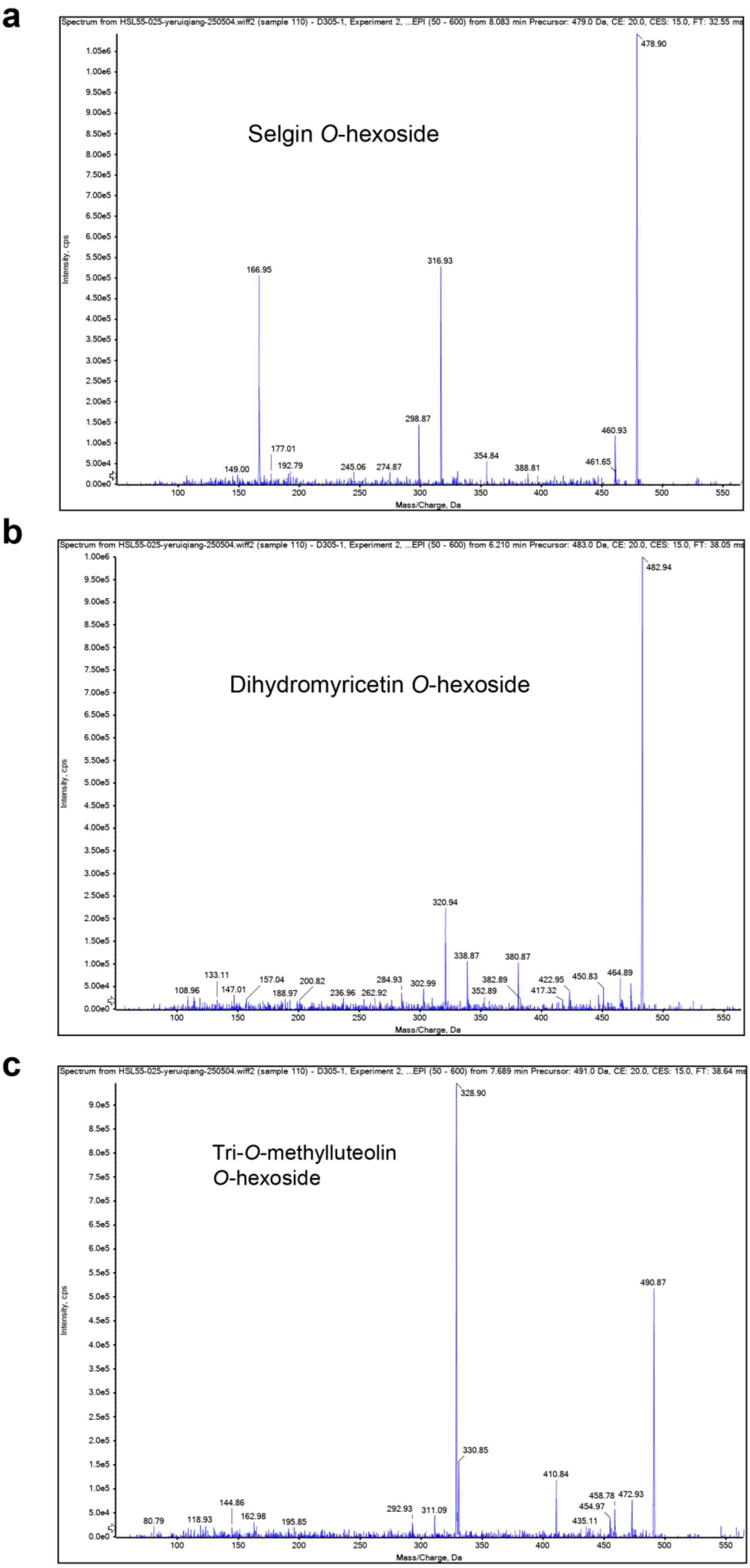

Figure S29. The MS/MS spectrum of Selgin *O*-hexoside (a), Dihydromyricetin *O*-hexoside (b), and Tri-*O*-methylluteolin *O*-hexoside (c).
